# Roblonski: A Material-Efficient Robo-Fluidic Toolbox for Rapid Photochemical Characterization

**DOI:** 10.1021/acscentsci.5c02027

**Published:** 2026-02-06

**Authors:** Azka Arshad, Richard B. Canty, Evgeny O. Danilov, Milad Abolhasani, Felix N. Castellano

**Affiliations:** † Department of Chemistry, 6798 North Carolina State University, Raleigh, North Carolina 27695-8204, United States; ‡ Department of Chemical and Biomedical Engineering, North Carolina State University, Raleigh, North Carolina 27695, United States

## Abstract

Reliable photochemical and photophysical characterization is essential for understanding and optimizing photocatalytic processes; however, traditional, manual spectroscopic methods for determining bimolecular photoreaction quenching constants, molar extinction coefficients, and photoluminescence quantum yields (PLQYs) are time-, cost-, material-, and labor-intensive and generate considerable chemical waste. Herein, we report Roblonski, a compact, material-efficient microfluidic robotic platform that automates these three foundational photochemical assays with high precision, reproducibility, and accuracy. Using Ru­(bpy)_3_(PF_6_)_2_ as a model photosensitizer and photocatalyst, we performed Stern–Volmer analyses with 11 excited state electron and triplet energy transfer quenchers, Beer–Lambert studies of five compounds spanning 3 orders of magnitude in their molar extinction coefficients across multiple solvents, and relative PLQY determinations for fluorophores/luminophores with PLQYs ranging over 3 orders of magnitude in efficiency. The machine-generated results matched manual experimental measurements and literature benchmarks across diverse spectral features, solvent environments, and signal intensity regimes. Roblonski reduces sample consumption (20-fold by solution volume, 1000-fold by reagent moles) and accelerates data collection (4-fold) compared to traditional, manual approaches. By integrating these photochemically relevant assays into a single, compact automated platform, Roblonski has the potential to lower experimental barriers, enable data-rich evaluation of photocatalysts and substrates, and augment autonomous photochemical discovery and characterization.

## Graphical abstract

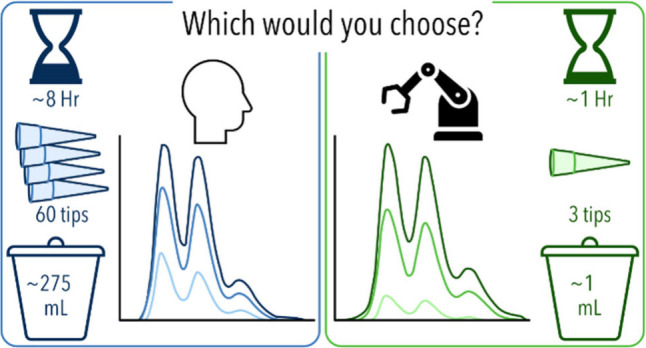

## Introduction

Photon-driven catalysis continues to offer new avenues to achieve challenging transformations with precise control, efficiency, and sustainability. Among its many branches, photoredox catalysis has emerged as a versatile strategy that utilizes visible light to initiate single-electron or energy-transfer events that access reactive intermediates under mild experimental conditions.^1–4^ This approach has expanded the synthetic toolbox for applications ranging across medicinal chemistry,^4,5^ sensing,^6,7^ energy conversion,^8,9^ and surface modification,^10,11^ among others. The compatibility of photoredox chemistry with green chemistry and process intensification principles—such as minimizing waste, avoiding the use of stoichiometric reagents, and operating at ambient conditions—has fueled its rapid adoption across disciplines.^12^ Quantitative photophysical characterization assays such as Stern–Volmer (SV) analysis, Beer–Lambert (BL) molar extinction coefficient determination, and photoluminescence quantum yield (PLQY) determinations are foundational to photocatalysis development. SV analysis elucidates quenching interactions between excited photocatalysts and reaction partners/substrates.^13–17^ BL measurements quantitatively determine light-absorption properties, yielding molar extinction coefficients that inform photocatalyst loading.^1^ PLQY measurements quantify the number of photons emitted relative to those absorbed, defining the photoluminescence (PL) signal intensities necessary to optimize the corresponding SV analysis and serving as a pertinent energy-efficiency metric for comparing photocatalysts. These assays provide mechanistic insights and reaction optimization yet require considerable time, effort, and material to perform—making them ideal for automation.^1,18,19^

Conventional workflows are labor-intensive, require extensive sample preparation, and consume substantial quantities of catalyst, quencher, and solvent, as well as other laboratory consumables, such as pipet tips. These burdens limit data throughput, can introduce considerable variability across data sets, and hinder the discovery of photocatalysts and photocatalytic reactions. While recent efforts have been made toward automating photophysical measurements, many existing systems require large experimental laboratory footprints and remain limited by material inefficiency, cost, or inflexibility.^20–24^

Here, we introduce Roblonski, a cost-, labor-, and resource-efficient, hybrid flow and batch (robo-fluidic) toolbox for the automated determination of photocatalyst–quencher SV quenching constants (*K*_SV_), photosensitizer BL molar extinction coefficients (*ε*), and PLQYs using the relative quantum yield method. Roblonski, named after A. Jabłoński, who first conceived the Jablonski diagram in 1935,^25^ combines automated access to a diverse photocatalyst and quencher library with multiassay capabilities within a compact microfluidic system (Figure 1). This system is optimized for minimal material consumption without sacrificing accuracy or precision. We first describe the platform’s design and performance, then highlight its efficacy by performing triplicate six-point SV analyses for the quenching of Ru­(bpy)_3_(PF_6_)_2_ photoluminescence by 11 different quenchers (operating under either electron or triplet energy transfer mechanisms) and a control sample. This study was completed within 25 h using only 4.3 mL of quencher solution (∼2 mM), 2.2 mL of catalyst (5 mM), 149 mL of solvent, and 100 mL of water. We further demonstrate the platform’s versatility by conducting five molar extinction and five PLQY campaigns across a library of established photocatalysts, chromophores, and fluorophores, yielding values that closely agree with those reported in the literature. Finally, we discuss the limitations of Roblonski in its current form and outline future directions.

**Figure 1:**
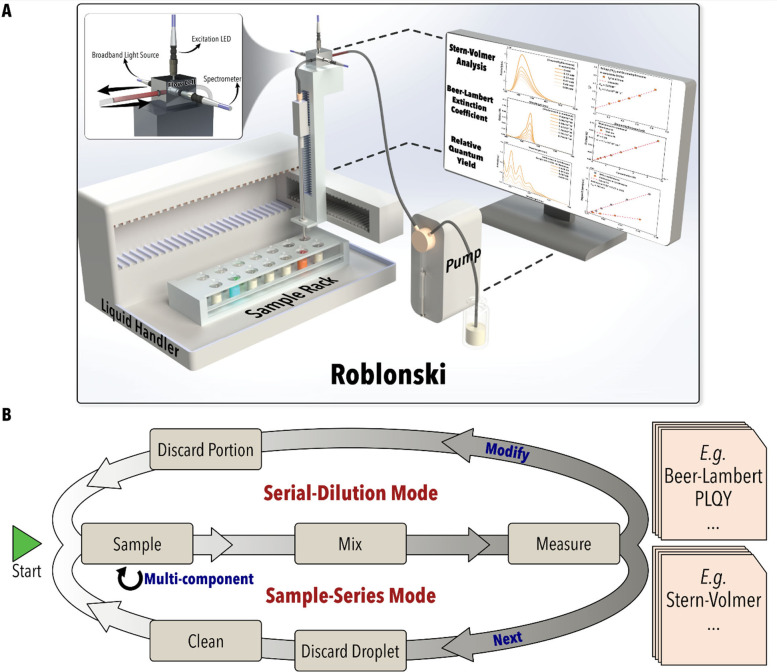
(A) Schematic illustration of the developed robo-fluidic platform (Roblonski) and (B) the general workflows implemented in this work.

## Results and Discussion

### Platform Instrumentation

To perform SV, BL, and PLQY studies on a single automated platform, we developed Roblonski around a Gilson GX-241 pipet-based robotic liquid handler (LH) and an OceanOptics QE-Pro photodiode array spectrometer (350–950 nm, effective). To act as a flow cell, we fabricated an aluminum support to align fiberoptics with a segment of the LH’s tubing just above the sampling pipet (Figure 1A, inset); these fiberoptics couple the spectrometer with a broadband UV–vis deuterium-halogen light source (OceanOptics) and a narrow-band UV LED light source (ThorLabs). The fluorinated ethylene propylene (FEP) tubing of the LH threaded through the flow cell was determined to have an effective optical path length of 0.10 cm. Integrating the flow cell module into the LH reduces the overall platform’s footprint, minimizes sample transport time (i.e., dead volume), and eliminates the need for additional pumps and injection ports for sample acquisition. The developed robo-fluidic platform ultimately requires 0.27 m^3^ of space (87 cm wide, 47 cm deep, ∼ 66 cm tall, excluding the computer and its peripherals), which is small enough to fit into a glovebox (Figure S1). As-is, Roblonski costs less than $30,000 USD (Section 2.2 of the Supporting Information); however, a replication using more current equipment on the market is estimated at around $70,000, with the dominant costs being the spectrometer and LH. The full platform layout, including a general photograph, an exhaustive parts list with model numbers, materials/dimensions for homemade elements, and distinctions between fabricated and commercial components, is detailed in the Supporting Information.

### Operation

Operators begin by loading 1.5 mL vials onto the LH deck along with a waste and wash vial/bottle. The operator then specifies vial parameters such as vial location, concentrations, spectroscopic acquisition parameters (such as a wavelength or wavelength range of interest, integration times and counts, and signal-processing parameters such as smoothing, peak finding/integration technique, *etc*.), sample names, and any assay-specific details (such as dilution factor and optical absorbance/PL signal targets for the BL and PLQY assays or quality-of-fit thresholds for the SV assay). Upon starting a run, the operator will be prompted for a system-priming volume, the volume of system backing fluid available, and for filename reconciliation if the proposed experiment names could result in overwritten data. The platform then performs liquid transfers, samplings, mixing, measurement, and cleaning operations automatically, saving raw and processed data as it goes. If signal target values are provided (BL and PLQY only), the platform will use the first measurements to determine if the stock solution needs to be diluted, and if feasible, will automatically dilute the stock solution before restarting the assay. The inclusion of signal targets affords the user some leeway with stock preparation. It is essential for reliable spectra, as deviations from the BL law occur at high optical density (sc., high concentration and high path length). If quality-of-fit thresholds are provided (SV only), the platform will perform a regression on the collected data and, if the thresholds are not met, automatically prepare two replicate samples to augment the data (Section 4.4 of the Supporting Information). Spectra for each sample are processed in accordance with the initial user input and are summarized (including transformed data and regressions) on a per-assay basis as specified by the workflow’s underlying Python file. After the automated assay is completed, the user may manually review, reprocess, and plot the data as desired.

In this work, a zero-faith operator is assumed, and throughput reports include the time required for the operator to inspect all data (as in Table 2) manually. Given that the analytes in this work have known spectral properties, an operator with no *a priori* knowledge of the analyte would either need to rely on the automatic peak-finding tools provided in the literature or perform a pilot, single-point experiment on each analyte. The time required to measure and review a single absorbance or PL spectrum of the raw stock solution is typically around 5 min.

### Calibration and Preliminary Characterization

To ensure the accuracy and precision of the intended assays, the platform’s fundamental performance was first characterized. The volumetric pipetting accuracy was measured gravimetrically (actual = nominal × 0.976 – 0.244 μL), and the calibration curve was precise (R^2^ = 0.9998) over the 0.5–50 μL aliquot range (Figure S2). No-transfer measurements permitted corrections for evaporation (∼0.25 μL/min). The effective optical path length of the 0.04 in. (nominal inner diameter) FEP tube was determined to be 0.102 ± 0.004 cm (0.039 in.) using a cuvette-measured zinc­(II) meso-tetraphenylporphyrin in acetonitrile calibration (Section 3.2 of the Supporting Information). Cross-contamination was measured via a mock SV workflow in which the catalyst and quencher vials were replaced with methylene blue stock solutions (contaminant), and the diluent vial (pure solvent) was periodically measured to determine its contaminant concentration. Different cross-contamination mitigation strategies were tested, ultimately resulting in a workflow with an estimated <0.15 μL contaminant per interaction rate of cross-contamination where vials are capped with a thin septum and no external washing of the LH’s needle is performed between sampling vials. Interestingly, washing the needle exterior with water or an absorbent tissue appeared to worsen cross-contamination. We hypothesize that external washing captures some of the wash at the needle tip, which cannot be properly expunged, and that the absorbent quickly becomes saturated (For a comparison of all four mitigation strategies, see Section 3.3 of the Supporting Information). Given the motion of the flow cell, to identify any artifacts from the fiberoptics or FEP tube moving and bending, incident light and PL signals were measured at random locations on the platform. No appreciable variation was found with coefficients of variation (CV; sample standard deviation normalized by the mean) less than 0.65% and 1.5%, respectively (Section 3.4 of the Supporting Information).

### Decoding Quenching with Stern–Volmer Investigations

In the first experimental demonstration by Roblonski, SV analysis was automatically performed on 11 quenchers and 1 control sample using a ruthenium­(II) photocatalyst (Figure 2) and subsequently benchmarked against both literature references and manual batch study measurements (Table 1). The automatic and manual studies are performed in open-air environments, where samples are exposed to atmospheric ground state triplet oxygen (O_2_), which can decrease the observed quenching constants. The literature references were selected to match these conditions whenever possible (see notes in Table 1).

**Figure 2:**
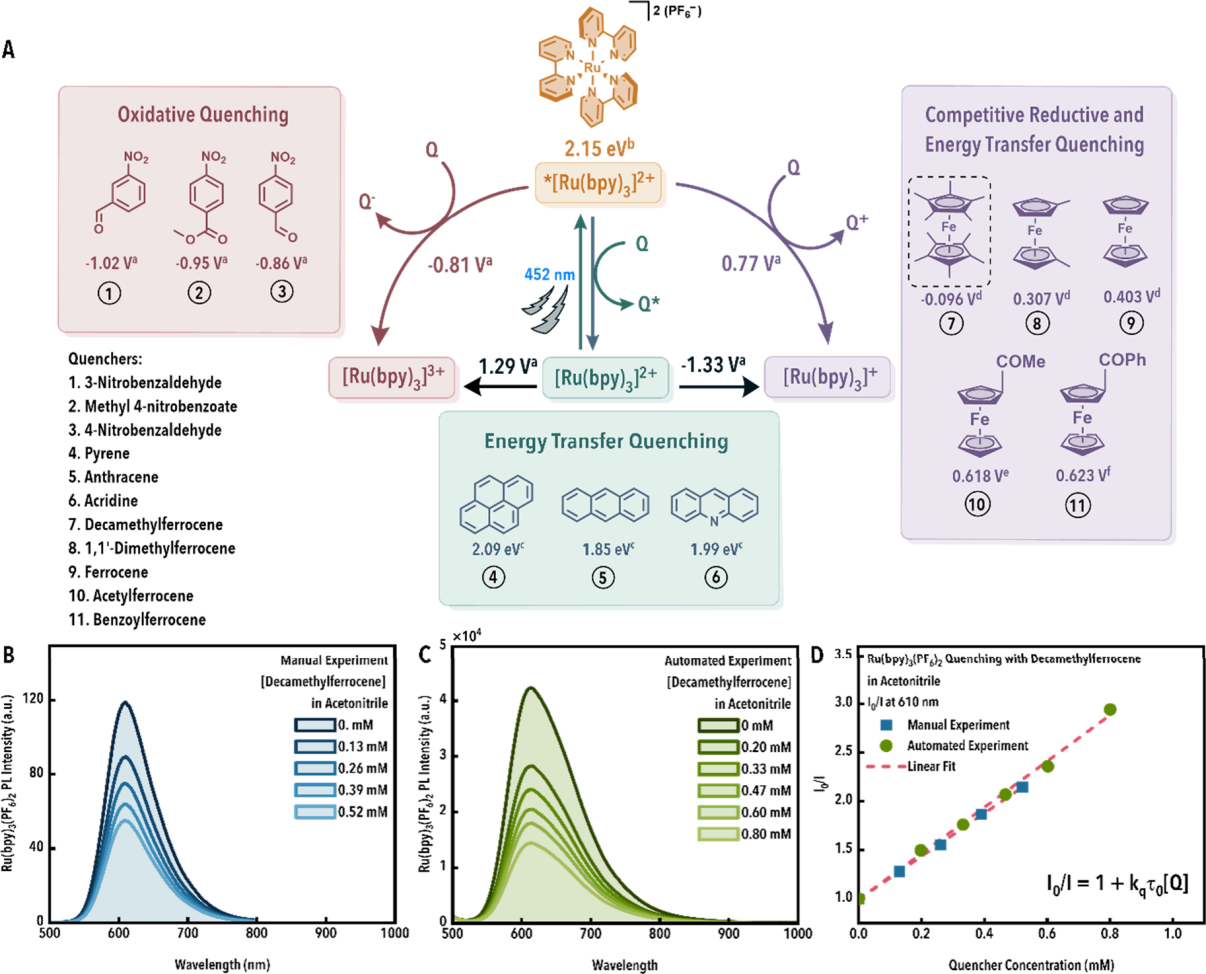
(A) Overview of the excited state quenching pathways for all quenchers (values reported in V refer to the reduction potentials vs SCE, while values reported in eV refer to triplet energies), (B, C) representative spectra (B: manual; C: automated) and (D) quenching (manual and automated) data exemplified using Ru­(bpy)_3_^2+^ as the catalyst and decamethylferrocene (dotted box in panel A) as the quencher. Referenced values: *a*,^28^ *b*,^26^, *c*,^29^ *d*,^30^ *e*,^31,32^ and *f*.^33^

**Table 1:** Comparison of Ru­(bpy)_3_(PF_6_)_2_ Quenching Rate Constants (*k*_*q*_, M^–1^s^–1^) Measured in both Automated and Manual Protocols^a^

	Modus				
	Automated			Manual	Literature
Quencher	K_SV_ (mM^–1^)	R^2^	*k*_q_ (M^–1^ s^–1^)	*k*_q_ (M^–1^ s^–1^)	*k*_q_ (M^–1^ s^–1^)
Acetonitrile (control)^b^	–0.001 ± 0.002	0.031	N.A.		
					
3-Nitrobenzaldehyde	6.0 ± 0.2	0.986	3.5 × 10^7^	2.8 × 10^7^	4.9 × 10^7^ Deaerated MeCN^28^
Methyl 4-nitrobenzoate	90 ± 5	0.974	5.6 × 10^8^	6.1 × 10^8^	6.8 × 10^8^ Deaerated MeCN^28^
4-Nitrobenzaldehyde	339 ± 6	0.997	2.1 × 10^9^	2.2 × 10^9^	2.2 × 10^9^ Deaerated MeCN^28^
					
Pyrene	212 ± 8	0.988	1.3 × 10^9^	1.5 × 10^9^	1.3 × 10^9^ Air MeCN^29^
Anthracene	140 ± 10	0.926	8.6 × 10^8^	50 × 10^8^	57 × 10^8^ Air MeCN^29^
Acridine	660 ± 20	0.995	4.1 × 10^9^	4.1 × 10^9^	3.8 × 10^9^ Air MeCN^29^
					
Decamethylferrocene	2370 ± 80	0.991	1.5 × 10^10^	1.4 × 10^10^	1.1 × 10^10^ Deaerated MeCN^26^
1,1′-Dimethylferrocene	1570 ± 70	0.984	9.8 × 10^9^	12 × 10^9^	11 × 10^9^ Deaerated MeCN^26^
Ferrocene	1270 ± 50	0.988	7.9 × 10^9^	9.1 × 10^9^	11 × 10^9^ Deaerated MeCN^26^
Acetylferrocene	1290 ± 40	0.991	8.1 × 10^9^	8.4 × 10^9^	3.4 × 10^9^ Deaerated EtOH^34^
Benzoylferrocene	840 ± 20	0.993	5.3 × 10^9^	13 × 10^9^	7.4 × 10^9^ Deaerated EtOH^34^

a Literature values are also provided. Full details of the measured *K*_SV_ values and their conversion to bimolecular rate constants are provided in Section 4 of the Supporting Information. Abbreviations: tris­(2,2′-bipyridine)­ruthenium­(II) bis­(hexafluorophosphate) (Ru­(bpy)_3_(PF_6_)_2_), Not applicable (N.A.), acetonitrile (MeCN), ethanol (EtOH).

b Analysis of the control treated the acetonitrile solution as a 19.15 M stock of quencher. Controls indicate a coefficient of variation in the catalyst signal of 0.2% (corresponding to a coefficient of variation in the *I*_0_/*I* signal of 0.2%) and that the expected concentration of the catalyst should vary less than 0.4%.

**Table 2:** Comparison of Temporal and Material Costs of Operation for Stern–Volmer Analysis between Various Automated Approaches and a Manual Approach, Assuming Comparable Stock Solution and System Warmup Times^a^

				Per intensity measurement (1 data point)		
	Catalyst Domain	Footprint	Per *K*_SV_ (*N* data points) Waste	Time^b^	Volume	Moles
Manual	Ru(bpy)_3_^2+^	N.A.	3 + 4*N* pipettes, 3 × ∼20 mL vessels, Wipes (as needed)	∼12 min Act., ∼18 min Auto.	3 mL (Total), ∼15 mL Wash	∼30 mM Q.^c^
Auto. Batch^d^ ^,^ ^38^	Cyanoarenes, Acridinium salts, Ruthenium complex, Iridium complexes	Liquid handle (152 × 87 × 112 cm)^e^ Plate reader (N.R.)	3 pipettes *N*/M, *M*-well plates, *N*/*M* silicon mats, Priming fluid 3× ∼1.5 mL vessel	<1/*N* min Act., 5 s Auto.	100 μL Cat., 48 μL Q., 52 μL Solv., ∼134 μL Wash	10 μM Cat., 20 mM Q. or ∼1.4 M Q
Auto. RF^39^	Ru(bpy)_3_^2+^, Iridium complexes, Polycarboazole phthalonitriles, Methylacridiniums	∼1/2 fume hood (if compacted)	3 pipettes, 1 × 20 mL vessel, 2 × 10 mL vessel, Priming fluid	∼0 min Act., 4.2 min Auto.	0.7 mL Cat. 1.3 mL Q. 1.7 mL Solv. 0.5 mL Wash	N.R. Cat., ∼33 mM Q.
Auto. Flow^f^ ^,^ ^40^	Rhodamine B, Fluorescein, Quinine	HPLC (N.R.) Computer (N.R.)Spectrometer (N.R.)	3 pipettes, 1 × ∼10 mL vessel, 2 × ∼30 mL vessel, 3–4 mL Wash	1–2 s Act., 5–8 s Auto.	13–19 μL Cat., 58–88 μL Q., 58–88 μL Solv.N.A. Wash	∼3 μM Cat., ∼100 mM Q.
This Work (RF)	(Same as Manual)	87 × 47 × ∼66 cm (excluding computer)	3 pipettes, 3 × 1.5 mL capped vials, 0.4–1.4 mL priming fluid	∼0 min Act., 8 min Auto.	10 μL Cat., ∼20 μL Q., ∼20 μL Solv., ∼0.6–1.1 mL Wash	5 mM Cat., ∼2 mM Q.^g^

a The methodology is described in Section 2.3 of the Supporting Information. Abbreviations: Automated (Auto.), Preparation (Prep.), Active (Act.), Not applicable (N.A.), Catalyst (Cat.), Quencher (Q.), Solvent (Solv.), Robo-fluidic (RF), Not reported (N.R.).

b Time is split into time where the operator must actively perform an action (Act.) and when an automated device is working (Auto.).

c Except for 3-nitrobenzaldehyde, which was 1960 mM.

d When comparing to batch (*sc*., well plate-based) approaches, the cost of an entire *M*-well plate for one *N*-experiment campaign should be amortized, as a well plate can be reused until every well has been contaminated.

e A Tecan Evo 100 liquid handler can be made to fit into a space of 107 × 78 × 87 cm; however, this instrument would no longer be easily serviceable.

f This study uses dynamic experiments. The method generates variable quantities of data due to signal thresholds; as a result, the ranges reported above represent the material cost of one experiment divided by the number of valid data points (which range from 360 to 580 in the four experimental demonstrations in the manuscript).

g Except for 3-nitrobenzaldehyde, which was 500 mM.

The photocatalyst Ru­(bpy)_3_(PF_6_)_2_ was selected as it is well-characterized in the literature; the quenchers were selected to span several orders of magnitude in their quenching rate constants (*k*_q_; *K*_SV_ divided by the PL lifetime measured in the absence of quencher) and to represent a diverse set of common quencher families. The selected panel includes nitroaromatic compounds, polycyclic aromatic hydrocarbons, and ferrocene derivatives, all of which quench Ru­(bpy)_3_^2+*^ via dynamic (collisional) quenching mechanisms and are expected to be resistant to measurement artifacts in the presence of air.^26,27^ Using photoexcited Ru­(bpy)_3_^2+^* in the presence of a quencher (Q), three distinct bimolecular reaction pathways are plausible: energy transfer (eq 1), oxidative electron transfer (eq 2), and reductive electron transfer (eq 3).

$$Ru{\left(bpy\right)}_{3}^{2+*}+Q\to Ru{\left(bpy\right)}_{3}^{3+}+Q^{*}$$ 1

2 $$Ru{\left(bpy\right)}_{3}^{2+*}+Q\to Ru{\left(bpy\right)}_{3}^{2+}+Q^{\text{−}}$$

$$Ru{\left(bpy\right)}_{3}^{2+*}+Q\to Ru{\left(bpy\right)}_{3}^{+}+Q^{+}$$ 3

Roblonski was programmed to prepare six quencher concentrations in triplicate and measure their relative PL intensities at a constant photocatalyst concentration for each photocatalyst–quencher pair. This study was completed within 25 h for the entire quencher library with no human intervention. In parallel, traditional cuvette-based SV experiments were manually performed under identical conditions to establish benchmarks for each quenching constant, requiring about 2 weeks to complete. Figure 2 presents the full series of molecular quenchers utilized, illustrating the quenching pathways from eqs 1–3, and provides representative quenching data, which will be discussed below.

Automated and manual *k*_q_ values (calculated using an experimentally determined τ_0_ of 160 ns for Ru­(bpy)_3_^2+^ in air-equilibrated acetonitrile) agree closely across all quenchers (Table 1). For ferrocene derivatives, quenching of the metal-to-ligand charge-transfer (MLCT) excited state of Ru­(bpy)_3_^2+^ proceeds primarily via reductive electron-transfer with *k*_q_ increasing with methylation degree (ferrocene <1,1′-dimethylferrocene < decamethylferrocene) as electron-donated methyl groups progressively lower ferrocene’s oxidation potential—enhancing the thermodynamic driving force for electron transfer consistent with Marcus theory.^26^ Conversely, acetylferrocene and benzoylferrocene display lower *k*_q_ values because their electron-withdrawing substituents raise the ferrocene’s oxidation potential, diminishing the driving force for electron transfer. In line with diffusion-controlled literature benchmarks, the deaerated ethanol *k*_q_ value for the Ru­(bpy)_3_^2+^–ferrocene system (∼5.9 × 10^9^ M^–1^ s^–1^)^34^ is roughly half that of deaerated acetonitrile (∼1.1 × 10^10^ M^–1^ s^–1^)^26^ owing to ethanol’s higher viscosity, slowing bimolecular encounters.^34^ The nitroaromatic quenchers all proceed primarily through oxidative electron transfer, but the extent and efficiency depend strongly on positional effects and substituent resonance. We recover the expected quenching behaviors where conjugation correlates positively with quenching^28^ (3-nitrobenzaldehyde < methyl-4-nitrobenzoate < 4-nitrobenzaldehyde).

All three aromatics (anthracene, acridine, and pyrene) quench the MLCT excited state of Ru­(bpy)_3_^2+^ through triplet–triplet (Dexter) energy transfer rather than electron transfer. Our manual and automated measurements reproduce literature values^29^ despite being measured under ambient conditions. Molecular oxygen itself is a strong triplet quencher of Ru­(bpy)_3_^2+*^, shortening the excited-state lifetime by nearly an order of magnitude under air-saturated conditions.^35^ For potent quenchers like 4-nitrobenzaldehyde, the effect is modest—our in-air, acetonitrile *k*_q_ values closely match deaerated literature benchmarks, since the intrinsic quenching is already approaching diffusional limits. However, for weaker quenchers such as methyl 4-nitrobenzoate and particularly 3-nitrobenzaldehyde, oxygen’s parallel quenching pathway disproportionately suppresses the observed quenching rate constants, leading to the apparent underestimation of *k*_q_. This explains why the manually and automatically measured air-saturated values, while mutually consistent, progressively deviate from deaerated literature values as the intrinsic quenching rate decreases. By integrating a near-infrared (NIR) detector (via substitution of the spectrometer and the fiber optics or incorporating a fiber optic splitter), the platform could capture oxygen’s phosphorescence signature (∼1270 nm), enabling quantitative separation of intrinsic and oxygen-induced quenching even under ambient conditions and turning a common experimental artifact into a mechanistic parameter.^36^ Nevertheless, the overall agreement between automated and literature data illustrates that Roblonski is suitable for evaluating triplet energy-transfer quenching processes.

Taken together, these results demonstrate the nuanced interplay between donor substituents, solvent properties, and mechanistic competition in dictating quenching dynamics. These results also highlight the importance of reporting experimental conditions—such as solvent and whether the samples are air-equilibrated or deaerated. Minor variations between automated, manual, and literature values fall within the expected experimental uncertainties, arising from subtle differences in excitation bandwidth, detector response, and sample handling. It is therefore important to consider that these discrepancies can become more apparent for quenchers with low solubility or overlapping absorption–emission features, where small spectral interferences can affect PL-based intensity measurements. In such cases, it becomes imperative to add necessary spectral corrections (*e.g.,* inner-filter correction) in the data processing routine. Nonetheless, the overall agreement across all quenchers and mechanisms confirms that Roblonski can rapidly and reproducibly determine *k*_q_ values over wide dynamic ranges using microliter-scale samples without sacrificing accuracy.

### Efficiency Characterization

To quantify the temporal and material costs of operation, Roblonski is compared against a manual and three automated SV assays, the latter of which were previously established by other investigators (Table 2).^38–40^ This comparison spans three modalities: parallel batch in well plates,^38^ serial robo-fluidic,^39^ and dynamic experimentation in flow.^40^ Per each SV campaign (the set of *N* intensity measurements required to determine a single SV quenching constant for a catalyst–quencher pair), Roblonski offers competitive advantages. Although our method is not as rapid in terms of data acquisition as other automated approaches, it uses far less material by volume (and only one implementation uses less material in terms of moles^40^), requires fewer or smaller consumables (glass vials, well plates, *etc*.), generates substantially less chemical waste (particularly compared to well-plate implementations), and is comparatively small and inexpensive. Overall, Roblonski demonstrates a marked reduction in time and human effort: achieving a 4-fold reduction in total time and a 2-fold reduction in active operator time (sample preparation and/or data analysis) for a SV study compared to manual operation.

The literature batch^38^ and flow platforms^40^ were demonstrated in only a single assay (SV analysis). In contrast, the literature reported a robo-fluidic platform^39^ that was shown in two modalities: a rapid screen for quenching activity and a complete SV analysis. Roblonski is designed for three independent photochemical assays without the need for experimental modifications and can be readily expanded to incorporate additional assays, such as dynamic PL experiments (lifetime determination).^41^ Notably, batch-like sample handling enables a wide dynamic range of attainable concentrations (via serial dilution), which can be challenging to achieve in purely flow-based platforms due to physical limitations on volumetric flow rate ratios at junctions. The costs of operation in the BL extinction coefficient and PLQY studies presented below are reported in Section 2.3 of the Supporting Information.

### Surveying Light Absorption Properties with Beer–Lambert Studies

To benchmark the accuracy and versatility of the platform for UV–vis absorption spectroscopy, we utilized Roblonski to automatically determine the BL molar extinction coefficients (*ε*) for a representative set of five well-characterized compounds having distinct classes of electronic transitions: zinc­(II) phthalocyanine, ferrocene, Ru­(bpy)_3_(PF_6_)_2_, rhodamine B, and perylene. These compounds were selected to span a broad dynamic range of *ε* values (96 M^–1^ s^–1^ at 442 nm for ligand-field d-d transitions in ferrocene to 277,000 M^–1^ cm^–1^ at 674 nm for π-π* transitions in Zn­(II) phthalocyanine)^42^ and to capture both simple and complex spectral profiles. Notably, perylene, an aromatic hydrocarbon, exhibits multiple absorption maxima due to the exhibition of vibrational fine structure in its absorption spectrum, enabling the evaluation of Roblonski’s ability to extract extinction coefficients at multiple wavelengths simultaneously. Solvents were chosen to ensure adequate solubility and spectral fidelity (Table 3).

**Table 3:** Comparison of Molar Extinction Coefficients (*ε*) Values between the Automated and Manual Protocols with Respect to Tabulated Values^a^

			Automated		Manual^42^		Literature
Compound	Solvent	λ (nm)	ε_λ_ (M^–1^ cm^–1^)	R^2^	ε_λ_ (M^–1^ cm^–1^)	R^2^	^42^ *ε*_λ_ (M^–1^ cm^–1^)
Ru(bpy)_3_(PF_6_)_2_	Acetonitrile	450	12,400 ± 500	1.000	13,300 ± 150	0.999	13,000
Zinc(II) phthalocyanine	Pyridine	674	235,000 ± 9,500	1.000	243,000 ± 980	1.000	277,000
Rhodamine B	Methanol	546	109,000 ± 4,400	1.000	121,000 ± 800	1.000	107,500
Ferrocene	Cyclohexane	440	91 ± 5	0.994	91.00 ± 0.78	1.000	96
Perylene	Cyclohexane	435.5	28,400 ± 1,200	0.999	32,500 ± 670	0.998	32,000
		408	21,900 ± 930	0.999	23,700 ± 500	0.998	23,500

a Full details of the measured ε values can be found in Section 5 of the Supporting Information. Abbreviations: tris­(2,2′-bipyridine)­ruthenium­(II) bis­(hexafluorophosphate) (Ru­(bpy)_3_(PF_6_)_2_).

In the automated workflow, each BL study was performed using 7 measured concentration–absorbance points, supplemented by an additional, origin-centered point for linear regression (a more diagnostic approach than linear regression with a fixed intercept). To maximize measurement precision, we implemented a working vial in line with the LH rather than a microfluidic droplet, a design choice that increased sample use per replicate (250 μL in vial vs 50 μL in droplet) but reduced the need for frequent replacement of flow-cell tubing and ensured stable signal performance over repeated runs (see Section 5.5 of the Supporting Information for a discussion of the rationale for this trade-off). For comparison, manual measurements were performed using the conventional cuvette-based approach, which involves preparing multiple dilutions and recording absorbance in standard 1 cm^2^ quartz cuvettes; see details in Section 5.2 of the Supporting Information.

The automated, manual, and literature values for BL extinction coefficients are presented in Figure 3 and Table 3. Roblonski demonstrates the capability to deliver highly linear absorbance–concentration relationships (R^2^ ≥ 0.998) with excellent reproducibility across all compounds investigated. Molar extinction coefficients obtained through the automated method closely matched both manually determined values and literature benchmarks, validating accuracy across a three-order-of-magnitude range of *ε* values and for both unimodal and multimodal spectra. Minor differences between the automated and manual results and literature benchmarks fall within the expected experimental and literature uncertainties, which for routine molar extinction coefficients are typically on the order of ∼5–10%, and arise from concentration determination, optical path length calibration, instrumental factors. In the case of perylene at 435.5 nm, the slightly lower *ε* value observed with our automated apparatus can be attributed to a subtle spectral feature: a small shoulder on the blue side of the main peak (Figure S8I,J). With the lower spectral resolution of Roblonski’s fiber-coupled spectrometer’s detector (Ocean Optics QE Pro) compared to the benchtop spectrophotometer used for manual measurements (Agilent Cary 60), the recorded λ_max_ falls between the shoulder and the true peak maximum, yielding a marginally reduced value. Importantly, this effect was isolated to just perylene’s first vibronic absorption peak and does not impact overall BL agreement trends.

**Figure 3:**
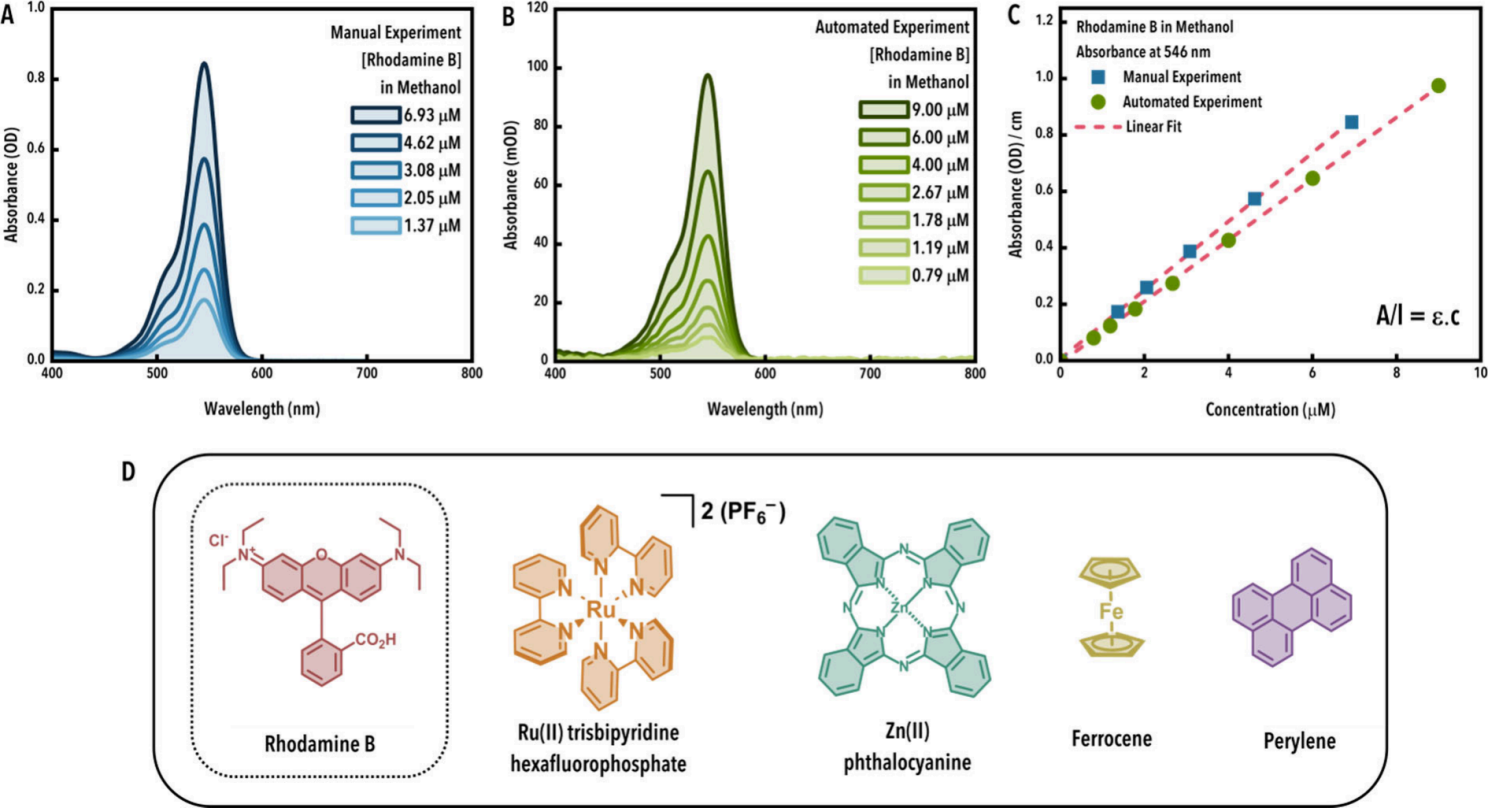
(A, B) Representative spectral (A: manual; B: automated) and (C) extinction (manual and automated) data exemplified using rhodamine B in methanol. (D) Overview of the compounds used in the Beer–Lambert study.

### Benchmarking Efficiency with Relative PL Quantum Yield Determinations

Next, to assess Roblonski’s ability for joint emission-absorption–based measurements, relative PLQY values were measured for a set of representative fluorophores (Figure 4A).^43^ For fluorophores that do not exhibit phosphorescence, PL measurements are effectively fluorescence measurements—PL will be used throughout the following text, as the detector was not set up to differentiate between the two. In this relative QY approach, both the reference fluorophore and the sample are measured in a dilution series, then the integrated PL intensity is plotted against 1–10^–A^ (where *A* is the absorbance at the excitation wavelength; Figure 4). The slope of the linear fit is proportional to the PLQY. With corrections for the solvent refractive index (*n*_D_), the relative slopes (sample vs standard) determine the absolute PLQY values. For this study, 9,10-diphenylanthracene (DPA) in cyclohexane excited at 365 nm served as the reference (fluorescence QY = 0.93 ± 0.03).^44^ Importantly, no optical cells are required for these quantitative determinations using Roblonski.

**Figure 4:**
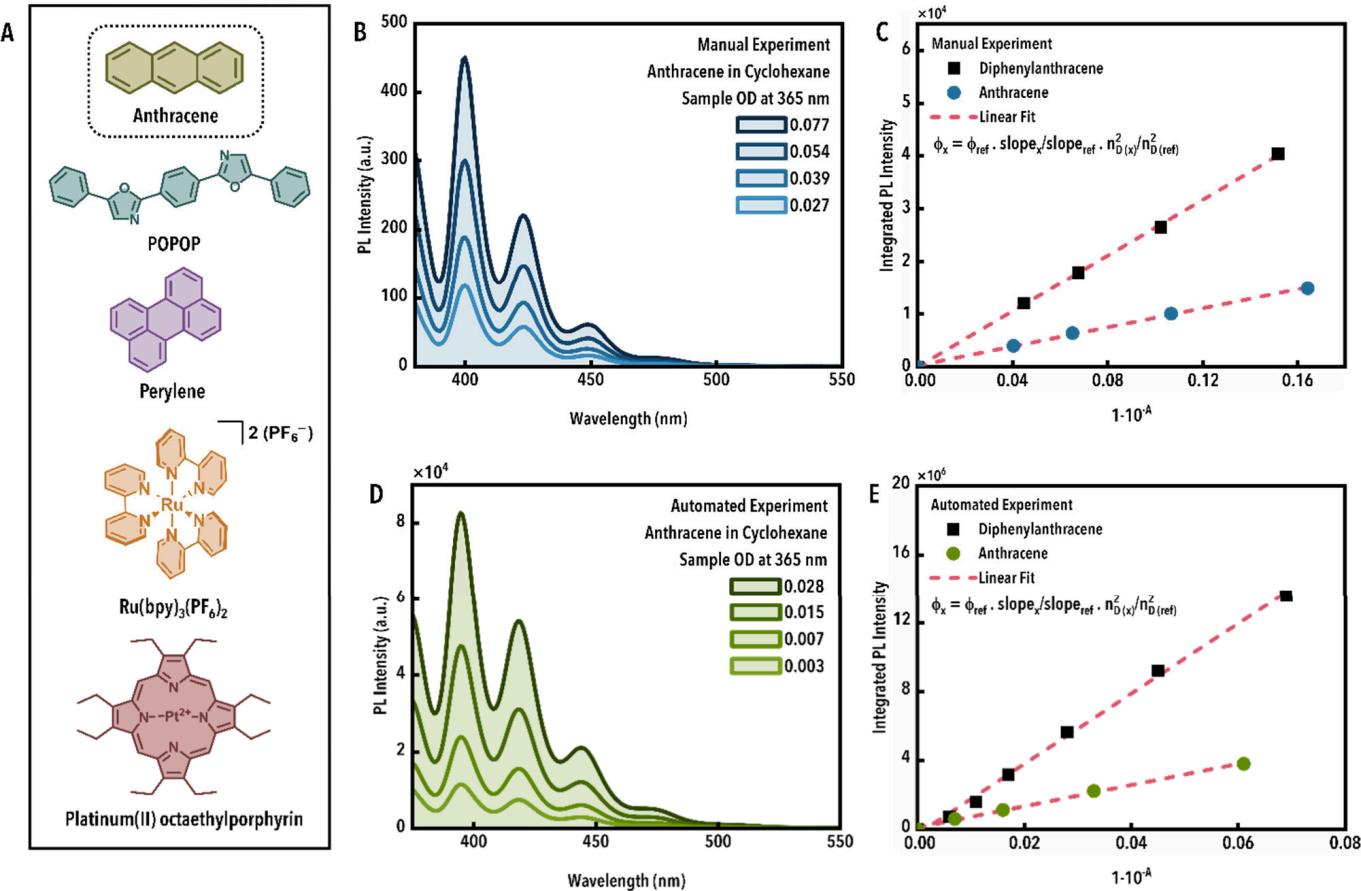
(A) Overview of the compounds used in the photoluminescence quantum yield (PLQY) study, (B) representative spectra, and (C) relative quantum yield plots exemplified using anthracene (manual), (D) representative spectra, and (E) relative quantum yield plots exemplified using anthracene (automated). Plots C and E use diphenylanthracene as the standard. Abbreviations: 2,2′-(1,4-phenylene)*bis*(5-phenyl-1,3-oxazole) (POPOP), tris­(2,2′-bipyridine)­ruthenium­(II) bis­(hexafluorophosphate) (Ru­(bpy)_3_(PF_6_)_2_), photoluminescence (PL), PLQY (ϕ), solvent index of refraction (*n*_D_), analyte (*x*), reference (*ref*), absorbance (*A*).

This slope-based implementation of the relative PLQY method offers several advantages, particularly within an automated context. By working with a series of dilutions and integrating the entire emission band, it minimizes errors from baseline offsets, wavelength-dependent detector sensitivity, and small fluctuations in excitation intensity. Plotting integrated PL intensities against 1–10^–A^ inherently accounts for concentration variations and ensures that each point on the calibration curve is physically meaningful. Furthermore, this approach is robust to small spectral shifts between samples and references. It can be performed without requiring absolute irradiance calibration of the detector, making it ideally suited for high-throughput measurements in a compact microfluidic apparatus. Minor differences between automated, manual, and literature values are within the expected experimental error ranges, reflecting variations in optical geometry, instrument response, material purity, and solvent microenvironments (Table 4). The inclusion of manual benchmarks under identical conditions provided a critical in-lab validation, confirming that Roblonski can accurately determine absolute PLQYs spanning roughly 0.1–100% across chemically diverse fluorophores and solvents.

**Table 4:** Comparison of Photoluminescence Quantum Yields (PLQYs) Values between the Automated and Manual Protocols and a Literature Reference^a^

Fluorophore	Solvent	*n*_D_ ^42^	PLQY (Automated)	PLQY (Manual)	PLQY (Literature)
DPA	Cyclohexane	1.426	Reference	Reference	0.93 ± 0.03 ^44^
Anthracene			0.27 ± 0.02	0.30 ± 0.01	0.28 ± 0.02 ^45^
POPOP			0.95 ± 0.04	1.00 ± 0.04	0.98 ± 0.03 ^46^
Perylene			0.90 ± 0.03	0.84 ± 0.03	0.94 ± 0.05 ^47,48^
Ru(bpy)_3_(PF_6_)_2_	Acetonitrile	1.344	0.020 ± 0.001	0.010 ± 0.0002	0.018 ± 0.002 ^45^
PtOEP	Toluene	1.497	0.0030 ± 0.0001	0.00030 ± 0.00002	<0.001 ^49^

a Excitation at 365 nm; full details of the measured PLQY values can be found in Section 6 of the Supporting Information. Abbreviations: solvent refractive index (*n*_D_), 9,10-diphenylanthracene (DPA), 2,2′-(1,4-phenylene)­bis­(5-phenyl-1,3-oxazole) (POPOP), tris­(2,2′-bipyridine)­ruthenium­(II) bis­(hexafluorophosphate) (Ru­(bpy)_3_(PF_6_)_2_), platinum­(II) octaethylporphyrin (PtOEP).

The automated PLQY measurement method, as deployed on this platform, reproduced literature and manual PLQY values across a wide range of emitters, solvents, and PLQY values. For example, disagreement between the automated, manual, and literature PLQY values for anthracene (literature value: 28%) and perylene (literature value: 94%) in cyclohexane does not exceed 6%p (z-score 1.4). At the lower end of the PLQY scale, the machine-determined PLQY for Ru­(bpy_3_)­(PF_6_)_2_ in acetonitrile fell within 0.2%p (z-score 0.9) of the literature value of 1.8%—demonstrating accurate performance even with low quantum efficiencies of photoluminescence emanating from the photosensitizer.

## Conclusions

Across the three case studies—SV quenching analysis, BL molar extinction coefficient determination, and PLQY measurements—Roblonski consistently reproduced manual and literature values with high fidelity despite diverse chemical systems, photophysical processes, and large dynamic ranges. We attribute this flexibility and precision, in part, to the synergy between batch-like sample preparation and microfluidic detector integration. The ability to perform serial dilutions to access a wide range of concentrations and the ability to automatically prepare new stock solutions on the platform facilitate the deployment of multiple assays on the same platform. Similarly, the microfluidic aspects of the platform enhance sample droplet mixing, minimize sample volumes, and mitigate inner-filter effects apparent in measured spectra. Overall, Roblonski can serve as a versatile multiassay photochemistry hub, tailoring trade-offs in efficiency, operational stability, and throughput.

### Limitations

In SV quenching experiments, the platform’s performance is subject to solubility and phase. While most quenchers exhibited in this study provided a good response at concentrations of around 2 mM, 3-nitrobenzaldehyde required a 500-mM stock to fall within the limits of detection. During preliminary testing of the platform, neat nitrobenzene was trialed; however, the variable solvent concentration across droplets challenged automated data processing. Furthermore, Roblonski is sensitive to precipitation—both in its ability to perform liquid-handling tasks as well as its ability to accurately measure spectra.

It is important to note that Roblonski is not autonomous. The operator must provide acceptable signal thresholds, provide estimates for where peaks are expected (or the means to locate said peaks automatically), determine satisfactory spectral measurement parameters (number of scans, scan spacing, and integration time), and prepare initial stock solutions at approximately satisfactory concentrations (Roblonski can only perform one attempt at diluting a user-provided stock solution to an acceptable concentration).

Extending Roblonski to new chemical systems or assays will likely require some user reconfiguration. Fixed platform properties such as the LH backing solvent, spectrometer slit size, flow cell tube diameter (i.e., optical path length), and fiber optic diameters may need to be adjusted between assays. Challenges such as changing light sources or extending the observable range beyond UV–vis are feasible using optical splitters and multiple light sources/detectors; these solutions, however, would greatly increase the overall cost of the platform.

### Future Directions

Looking forward, Roblonski’s solvent compatibility and adaptability position it for quantitative studies in photochemical kinetics,^50,51^ photochromism,^52^ photochemical upconversion,^53–55^ and dynamic photoluminescence measurements. Similarly, Roblonski’s flexibility makes it amenable for the spectroscopic evaluation of both soft and hard, organic and inorganic materials, semiconductor nanocrystals, metal–organic frameworks, and other valuable photonic materials platforms.

The SV results highlight a key conceptual sight for future work: quenching rate constants alone cannot fully reveal mechanisms, as **c**omparable *k*_q_ values may arise from electron transfer, energy transfer, or a combination of both. Disentangling these pathways requires complementary time-resolved techniques, such as nanosecond or femtosecond transient absorption spectroscopy, which directly probe charge-separated or triplet intermediates. Beyond NIR-based singlet oxygen correction, an interesting avenue for advanced SV data processing would be to automate the selection of the underlying quenching model (e.g., the Lehrer-adjusted quenching mode^37^).

Near-term goals for the Roblonski platform include integrating with laboratory-assistance software (such as Co-Scientist)^56^ to provide a more nonprogrammer-friendly interface and incorporating lifetime measurement assays via time-correlated single-photon counting or gated detection. Additional potential specializations of the Roblonski platform include incorporating a heater into the LH bed to enable online synthesis or a heating jacket on the fluidic line to provide temperature and solubility control. Furthermore, a microreactor element could, in principle, be added above the flow cell with modifications to the pumping backend to accommodate the added system line volume. The ultimate integration of Roblonski with a synthesis platform and machine learning could facilitate autonomous photochemical research.

## Supplementary Material





## Data Availability

The code for this demonstration of Roblonski is available free of charge as a public Zenodo-archived GitHub repository (https://github.com/RBCanty/Roblonski).^57^

## Notes

Detailed descriptions of Roblonski’s hardware and their associated costs, experimental consumable estimates, descriptions of all three spectroscopic assays, calibration and characterization details, and their error estimations, methods, data analysis, and all quantitative details regarding automated and manual experiments executed in the development of this manuscript (PDF)
Raw and processed spectral data (manual and automated) (ZIP)
