# Supplementary material for: Roblonski: A Material-Efficient Robo-Fluidic Toolbox for Rapid Photochemical Characterization
Source: ACS Cent Sci. 2026 Feb 6;12(3):333–44. doi: 10.1021/acscentsci.5c02027 (PMC13022720; doi:10.1021/acscentsci.5c02027)
Supplement: Supplementary file 1 [file oc5c02027_si_001.pdf]

Supporting Information for:

Roblonski: A material-efficient robo-fluidic toolbox for rapid photochemical characterization

Azka Arshad[a‡], Richard B. Canty[b‡], Evgeny O. Danilov[a], Milad Abolhassani*[b], Felix N. Castellano*[a]

[a]Department of Chemistry, North Carolina State University, Raleigh, North Carolina 27695-8204, United States

[b]Department of Chemical and Biomedical Engineering, North Carolina State University, Raleigh, North Carolina 27695, United States

[‡]Azka Arshad and Richard B. Canty both serve as co-first authors for this work.

*Corresponding author email: abolhasani@ncsu.edu

*Corresponding author email: fncastel@ncsu.edu

1. Abbreviations

Abbreviation	Definition
Abs	Absorbance/Absorption
A.U.	Arbitrary units
Av.	Average
CV	Coefficient of variation
DPA	9,10-Diphenylanthracene
Ea.	Each
FEP	Fluorinated ethylene propylene
λ_{max}	Wavelength at maximum absorbance
LED	Light-emitting diode
MAE	Mean absolute error
N.A.	Not applicable
Norm.	Normalized
OD	Optical density
PL	Photoluminescence
PLQY	Photoluminescence quantum yield
POPOP	2,2'-(1,4-Phenylene)bis(5-phenyl-1,3-oxazole)
PtOEP	Platinum(II) octaethylporphyrin
RMSE	Root mean square error
$Ru(bpy)_3(PF_6)_2$	Tris(2,2'-bipyridine)ruthenium(II)
SV	Stern–Volmer
USB	Universal serial bus
USD	United States dollars
UV	Ultraviolet (light)
UV–Vis	Ultraviolet–visible (light)
ZnPc	Zinc phthalocyanine
ZnTPP	Zinc(II) meso-tetraphenylporphyrin

2. Platform cost and footprint

2.1. Platform footprint estimation

The platform comprises two light sources (ultraviolet–visible, UV–Vis, and single-wavelength UV), a pump, a liquid handler, a bespoke flow cell, and a spectrometer. These components can be arranged in an easily serviceable configuration within a roughly 2-by-3-foot area (**Figure S1**). The reported height of the platform at ~66 cm is based on the maximum height of the excitation fiber optic as the arm moves—which should not be obstructed lest the cable be bent beyond its operational limit.

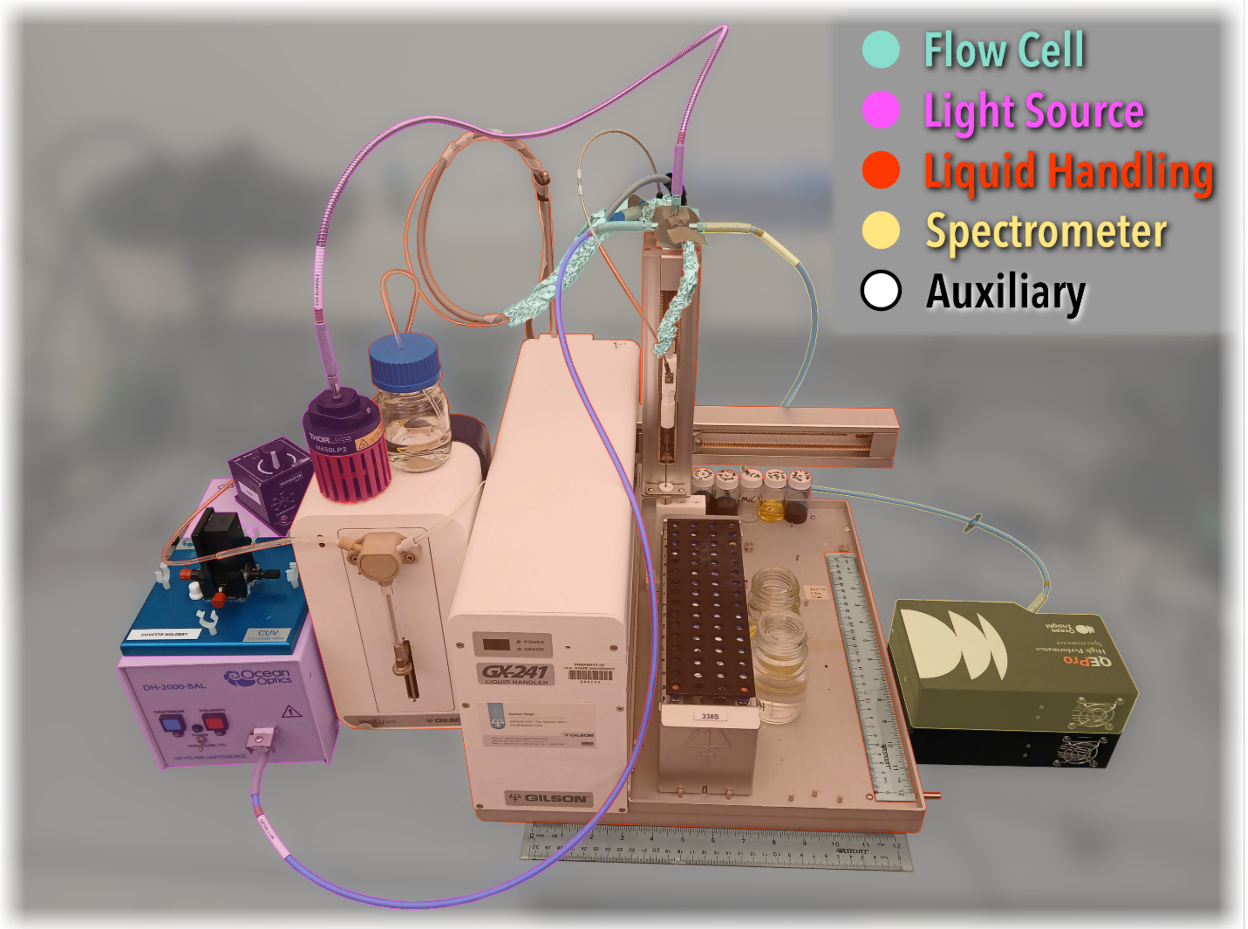

Figure S1. Color-annotated photograph of the Roblonski platform in a compacted orientation, 1-foot rulers for scale. (Cyan) Bespoke flow cell and surrounding light shroud; (Magenta) Broadband UV-Vis and UV LED light sources, fiber optic cables, and UV LED controller dial; (Red) Liquid handling robot with pump, fluidic tubing, and sample vials; (Yellow) Spectrometer and fiber optic cable; (Unshaded) Auxiliary devices used during platform development—a cuvette holder, left, and second spectrometer, right—and rulers for scale.

2.2. Platform cost estimation

The cost of this platform is around 30,000 USD. This estimation is itemized in **Table S1** below. In both the automated and manual cases, the gross cost is dominated by the spectrometer(s).

Table S1. Cost breakdown based on quotes with academic discount and listings on public marketplaces in March 2025.

**Item**	**Approximate Unit Cost (USD)**	**Count**
Liquid Handler [a]	~2,000 (as-is)<br>30,00–50,000 (new)	1
UV–Vis Light Source	3,000–5,000	1
UV LED Light Source	500–1,000	1
Fiber Optic Cables	100–200	3
Additional tubing and fluidic connections<br>(Not part of original Liquid handler)	<100	N.A.
Spectrometer	15,000–25,000	1
Flow Cell<br>*Aluminum prism*<br>*Three holes*<br>*Three threaded holes*	<1,000	1
Computer and Software	~1,000[b]	1
Software[c]<br>PyCharm Community Edition<br>Python 3.12<br>*Pyserial, Nidaqmx, Numpy,*<br>*Pyseabreeze, Scipy, Libsub,*<br>*Peakutils, Pandas, Pyusb*<br>Zadig USB Driver for Spectrometer	0	1 ea.
**Gross**	~29,000 or 22,000–34,000[d] (as-is)<br>~69,000 or 56,000–80,000[d] (new)	
**Manual (Reference)**<br>UV–Vis spectrometer<br>Fluorimeter<br>Cuvette<br>Computer and Software	 14,000<br>33,000<br>~300<br>~1,000	
**Gross**	~48,000	

[a] The GX-241 liquid handler (the unit used in this platform) is a comparatively older model and is available for under 2,000 USD aftermarket; modern Gilson liquid handlers are available for 10,000–30,000 USD (used) or 30,00–50,000 USD (new).
[b] The computational requirements for running python would permit an economy computer; however, most facilities would likely use an existing laboratory computer.

[c] All software—Python 3.12, Python packages (italics), and USB drivers—used for this work are free.
[d] The gross range is estimated by treating the half-range of each item as a standard error.

2.3. Assay cost estimations
The method for estimating the time, waste, and material costs of the Stern–Volmer (SV) assay across the five approaches presented in **Table 1,** as well as the estimated costs for the automated Beer–Lambert and photoluminescence quantum yield (PLQY) studies, is presented below. The metric of material use (average molarity of samples in quencher and catalyst) is provided not as a definitive metric of material use, but as a touchstone. In practice, the concentrations utilized will be a function of the solubility of the solute as well as the necessary concentration to achieve a desired signal-to-noise ratio in the spectral data. Nevertheless, stratification into micro-, milli-, or uni-molar consumption gives a general sense of how material-intensive a protocol is. Furthermore, additional optimizations can be made when measuring multiple catalyst-quencher quenching constants, such as using a single stock for the common catalyst or common solvents—which reduces the number of vessels discarded for each measured quenching constant—or using the same pipette between identical stock vessels.

Manual
The method for estimating the time, waste, and material costs of the Stern–Volmer assay in our manual experiments is described below. The following calculations are for the complete set of 12 quenchers tested in triplicate, with each replicate consisting of 5 quencher concentrations, yielding 180 total measurements. For each measurement, a 3 mL sample solution was prepared from catalyst, quencher, and solvent stocks—yielding a total sample consumption of 540 mL (180 measurements × 3 mL/measurement). The cuvette was washed ~5 times after every measurement until there was no absorption or PL signal was detected. One washing cycle consumed ~15 mL, resulting in ~2.7 L (180 measurements × 15 mL/measurement) of the washing solvent. In total, bringing the total solvent usage to ~3.3 L across the whole campaign.

After the creation of the photosensitizer, quencher, and solvent stocks (3 pipettes), an average of ~4 tips per measurement (or ~20 tips per replicate experiment) were used over the course of the 180 measurements, corresponding to ~720 pipette tips in total. In practice, these tips were distributed across catalyst, quencher, and solvent additions; the pipette tip used for solvent addition at the end was also used for mixing, and a fourth pipette tip was used for the washing steps.

The total duration of the Stern–Volmer experiments was estimated by accounting for daily instrument warm-up, stock solution preparation, sample handling, data acquisition, and post-processing. Approximately 15–20 minutes per day were devoted to instrument startup and warmup of the Cary UV–Vis spectrophotometer and the Eclipse spectrofluorometer. In addition, ~30 minutes were required for preparing stock solutions, which included weighing solids and dissolving them in solvent; in some cases, additional time was needed to ensure homogeneity, depending on solubility constraints. For each measurement, ~5–7 minutes were required for sample preparation and ~15–20 minutes for spectrometer operation, resulting in an average of ~23.5 minutes per data point. Across the complete data set of 180 measurements, this corresponds to ~70 hours of experimental runtime. Data analysis also represented a significant time investment: for each quencher (tested in triplicate, 15 measurements), ~2 hours were required for cleaning, plotting, and fitting of the Stern–Volmer data in Origin 2023b—bringing the estimated active time

dedicated to each data point from 6 to 12 minutes. When combined with daily warm-up overheads (~3.5 hours across the campaign) and stock solution preparation (~0.5 hours), the total estimated duration of the manual Stern–Volmer study was ~95–100 hours. This comprehensive time commitment underscores the resource- and labor-intensive nature of manual Stern–Volmer analysis, even before accounting for further repetitions or troubleshooting.

Stock preparation (**Table S2**) dramatically affects the total material usage. In practice, chemists routinely prepare stock solutions in volumes larger than the immediate need to ensure precisely measurable quantities (*sc*. electronic balance precision), reproducible pipetting, adequate volumes for replicates and mistakes, and minimal errors from solubility or evaporation. In this work, the stock solutions used in the automated studies were prepared as samples/dilutions of the manual studies' stocks.

Table S2. Stock solution parameters for the manual and automated studies.

Compound	Manual conc. (mM)	Manual vol. (mL)	Automated conc. (mM)	Automated vol. (mL)
$Ru(bpy)_3(PF_6)_2$	5.00	20.00	5.00	5.00
Ferrocene	10.0	10.0	1.50	4.00
Acetylferrocene	10.0	5.00	1.50	4.00
Decamethylferrocene	3.91	4.00	1.00	4.00
Dimethylferrocene	3.70	4.00	1.00	4.00
Benzoylferrocene	10.0	7.98	1.00	4.00
3-Nitrobenzaldehyde	1960	4.946	500	4.00
4-Nitrobenzaldehyde	50.0	13.49	5.00	4.00
Methyl 4-nitrobenzoate	145	10.42	20.0	4.00
Anthracene	12.226	5.42	2.50	4.00
Pyrene	65.73	8.09	10.0	4.00
Acridine	15.0	10.68	3.00	4.00

Concentration (Conc.), Volume (Vol.).

Automated Batch

As reported by Motz *et al.*[1], this high-throughput method for determining Stern–Volmer quenching constants operates in 96-well plates (M=96). In their method, eight quencher concentrations (N=8) are used against a single photocatalyst. Because an entire well plate is not consumed upon the use of a subset of its wells, the well plate is only discarded after all wells have been used. Similarly, the silicon mats covering the plates can be used until all access points have been contaminated. In general, this approach consumed N/M well plates and mats for each quenching constant measured.

As this platform uses a liquid handler, some priming fluid will be required to ensure the pumps and liquid lines are free of bubbles. This value is not reported and would vary with the specific liquid handler setup and plumbing. Furthermore, the platform operates by transferring liquid from a reservoir to a destination. To dispense 100 µL of catalyst into all 96 wells (*Microplate Preparation*[1]), there must be a reservoir of at least 9.6 mL of catalyst. Similarly, for a full plate, the volumes of quencher and acetonitrile listed must yield at least 4.62 mL of quenchers and 4.98 mL of acetonitrile on the platform. On a per-quenching constant basis, this would correspond to 1.2 mL of catalyst, 385 µL of quencher, and 415 µL of acetonitrile. These requirements were generalized to three 1.5-mL vessels (a typical vial size for small volumes).

The dimensions for a Tecan EVO 100 platform are reported by Tecan in their operating manual (Tecan document number 392886, English, version V10.0) in section 3.2.1, which is available from Tecan upon request.

The example presented in *Microplate Preparation*[1] specifies that for wells A–H, 100 μL of catalyst is used per well, that the quencher volume varies (0, 10, 25, 40, 55, 70, 85, 100 μL), and that the diluent volume varies (100, 90, 75, 60, 45, 30, 15, 0 μL). Thus, the average material used per well (per data point) is 100 μL of catalyst, 48 μL of quencher, and 52 μL of diluent.

The example liquid-handling protocol presented in the *liquid-handling robot protocol*[1] specifies multiple washes during handling of a full plate. By amortizing the total volume of wash solvent used over the whole campaign of 11 plates (142 mL) by well (1056 wells), the estimated washing cost per data point is determined to be approximately 134 μL.

In their *Results and Discussion*, it is stated that "one photocatalyst could be analyzed on a plate in less than one minute" and "to prepare plates for each of 20 common photocatalysts in <2 h […] for a total of 1920 samples to generate 220 quenching constants"[1]. Given the nature of the study, which measures a single photosensitizer against multiple quenchers, and that each quencher was evaluated at 8 concentrations, these statements are unfortunately ambiguous. It is assumed that the reported system can measure one well plate in one minute. Furthermore, 20 photosensitizers, 12 quenchers, and eight quenching concentrations would require 1920 samples and yield 240 quenching constants. Using 1920 as the number of data points prepared in 2 hours (4 seconds per sample preparation), and one 96-well plate measured in 1 minute (~1 second per data point), this approach would take ~5 seconds per data point. Finally, the manuscript does not specify, but the automated workflow presented in *Microplate Preparation*[1] does not include any plate transfer steps, it is assumed that <1 minute is required for a user to transfer each plate from the liquid handler to the plate reader for measurement.

The example in *Microplate Preparation*[1] specifies that the quenchers are at 20 mM and the catalyst at 10 μM. In contrast, Table S5 reports the quencher concentrations on an example plate, and the maximum concentrations range from 587 to 728 mM, suggesting that the stock quencher solution concentrations should be higher. The prior figures of 100 μL of catalyst, a minimum diluent volume of 0 μL, and a total volume of 200 μL per well would imply that these stocks would be 1.2–1.5 M. It could be possible that this plate was prepared differently (wherein the platform solved for the necessary volume of catalyst, quencher, and diluent to achieve a set of target concentrations), in which case a 20 mM stock of quencher could yield 587–728 μM conditions in the well. A typo in units would be an understandable error and is precedented by their Figures 3 and 6 ($Ir(ppy)_3$ validation studies), with abscissa ranging up to around 3 mM, whereas their Tables S1 and S3 report quencher concentrations to around 4 μM.

Automated Flow 1

As reported by Kuijpers *et al.*[2], the high-throughput method for determining Stern–Volmer quenching constants operates in a flow configuration and is intended for both quick screening and full Stern–Volmer assay modes. In the present comparison, we base our analysis exclusively on the Stern-Volmer assay reported in their work. This analysis further assumes a correct mapping between the Stern–Volmer plots present in Scheme 4 of the Kuijpers et al. publication[2] and the experiment timestamped "2017-11-23 14:31:38,571" in their associated log file hosted on GitHub (SternVolmerLog.txt).

For this experiment, the catalyst flow rate was held constant at 0.2 mL/min and the solvent and quencher flow rates were varied inversely to maintain a total flow rate of 1 mL/min. The set

of flow rates used was: 0, 0.16, 0.32, 0.48, 0.64, and 0.80 mL/min. Timing each data point from the timestamp when the solvent pump was set, the protocol used a 3-minute purge with pure solvent, followed by a 6-minute quencher-free measurement, then six 3.2-minute measurements of the remaining five data points. The campaign reported used 13 mL solvent, 4.4 mL catalyst, and 7.7 mL quencher over 25 minutes—thus, a campaign would expect two 10-mL vessels and one 20-mL vessel to hold all the required material. Per data point, this averages to 2.2 mL solvent (23% allocated to washing), 0.7 mL catalyst, 1.3 mL quencher, and 4.2 minutes. Finally, based on the logs, the most concentrated quencher condition was 26.56 mM (quencher flow rate 0.8 mL/min, catalyst flow rate 0.2 mL/min), thus implying a quencher stock solution of 33.2 mM.

The rough estimation of the footprint is derived from their Figure S2[2], which shows the system occupying half of a fume hood with some equipment outside the hood. There appears to be space to move the external equipment into the fume hood without increasing the footprint.

Automated Flow 2

As reported by Desilets *et al.*[3], this high-throughput method for determine Stern–Volmer quenching constants operates via a dynamic experiment approach wherein the flow rates of catalyst, quencher, and ionic solvent were co-varied and on-line spectral measurements measured the entire trace.

As stated in the *Methods* and *Gradient Linearity and Instrument Stability* sections of that manuscript[3], the assay comprised a 10-minute hold at 10% catalyst, 90% solvent, 0% quencher, followed by a 15-minute ramp from these conditions to 10% catalyst, 0% solvent, 90% quencher, followed by a 10-minute hold at these conditions. The total flow rate is stated as 2 mL/min (*Solvent Delivery and Detection* section[3]). From these figures, the whole campaign requires 7 mL of catalyst, and 32 mL, each, of solvent and quencher—thus a ~10-mL vessel and two ~30-mL vessels would be required per run. Based on Figure 1, the approximately 2-minute lag time would imply a dead volume of 3–4 mL; heuristically, between campaigns (*i.e.*, between k_q measurements) there should be a wash of at least 3–4 mL.

The material use per data point is very low for this platform due to its nature as a continuous/dynamic experiment. As reported in their Table 1, the number of useful data points varied from 360 to 580. Dividing the material used by the number of data points generated yields estimates of 13–19 μL of catalyst, 58–88 μL of solvent, and 58–88 μL of quencher per data point.

The authors state that the gradient is 35 minutes and that the estimated time for a quenching constant was 45 minutes (*Sources of Error*[3]); using the estimated 45 minutes and normalizing by the number of data points generated yield an estimated 5–8 seconds per data point. Finally, the discrepancy between the 35-minute gradient and the estimated 45-minute duration would imply a roughly 10-minute window when the user is needed (amortized to 1–2 seconds per data point).

The physical platform consists of a high-performance liquid chromatography unit (used for its timed, metered pumping), a computer, and a spectrometer. The sizes of these units were not listed, and while they could in principle be determined, the work of Desilets *et al.* [3] dates to an early generation of laboratory instrumentation (1987), and the physical footprints of comparable components have since been substantially reduced with modern hardware.

Finally, the concentrations of the stock solutions are reported in their *Solvents and Reagents* section: 2.5–5.1 μM catalyst and 50–200 mM quencher, with the exception of the iodine quencher, which was prepared at 100 μM.

This work

In our work, the material consumption was known. For each quenching constant, three 1.5-mL vials were used (one each for the catalyst, quencher, and solvent). In practice, we used the same catalyst vial for three quenchers—a total of 4 catalyst vials for 12 quenching constants. Similarly, a pipette tip was used for each species. Again, in practice, the same pipette tip can be reused for common species.

The platform's footprint could be directly measured. We note that shelving would further reduce the footprint, and that the spectrometers could be set on their sides to reduce it marginally as well. The configuration photographed in **Figure S1** shows the system in a compact, but still easily serviceable configuration, and was used for the 87×47 cm footprint. The height was estimated at ~66 cm due to the flexibility of the fiberoptic cable coming out of the top of the flow cell (excitation optical path) and its shifting curvature/height as the liquid handler arm moves around the deck.

Priming fluid consumption is reported as 400–1400 μL. The total volume of the flow cell and needle was 213 μL, so a 400-μL prime was used when immediately restarting the system. Conversely, the volume from pump to needle was near 1400 μL, so a large prime was used when the system had been idle or when air bubbles were observed in the fluid line.

The duration for a single data point to be measured was estimated at 8 minutes based on the difference in timestamps in the data files generated during operation. The ~0-minute active time is calculated from the short amount of time required to place the vials onto the deck and specify the vial components (name, concentration, target wavelengths for analysis, *etc.*) during the initial setup.

The material used is similarly known: 10 μL was taken for the catalyst component of each droplet, and the volumes of solvent and quencher per droplet average to about 20 μL each (1 droplet = 1 data point). Furthermore, the unoptimized cleaning protocol uses 600 μL of solvent for washing. In addition, a ~100 mL wash vessel is provided on the platform; it is replaced as needed. Given one 100-mL wash vessel was used during the triplicate study of 12 quenchers using 6 data points (*sc.* some used 8, but 6 is used for all quenching constants in this estimate), on average ~500 μL of wash was used per data point. Combined, the cleaning and washing protocols use 600–1100 μL of wash per data point. This could be improved further by minimizing the volume used in the cleaning protocol (by measuring contamination) and by reducing the frequency of replacing the wash bottle (by measuring contamination). Finally, the concentrations of the catalyst and the quenchers are known and reported in this work: 5 mM for the catalyst and ~2 mM for the quenchers (except for 3-nitrobenzaldehyde, which was 500 mM).

Automated BL and PLQY Assays (This work)

The material use of the BL and PLQY assays is identical. Both use three 1.5-mL vials per compound: a source vial containing ~0.5 mL of analyte, an empty vial, and a diluent vial containing ~1.5 mL of solvent. A total of 2 pipette tips are required to transfer the stock solutions into the source and diluent vials. The startup priming and inter-sample cleaning volumes are identical to the SV assay: 400–1400 μL of system fluid for priming and 600 μL of solvent for cleaning. In addition, these assays use a 100-mL water bath to clean the outside of the needle.

Material use is variable. If the concentration of the source vial meets the target initial optical density, then 300 µL of sample, 500 µL of diluent, and 600 µL of washing fluid are consumed. If the optical density is such that a diluted stock is automatically prepared, then at most 300 µL of sample, 1390 µL of diluent, and 1200 µL of washing fluid are consumed. The average concentration of the sample for the BL assay was 0.09 mM (excluding Ferrocene, which was 7 mM).

For the BL assay, based on the difference in timestamps of the spectra files, the average time between measurements was 6 minutes—for a total duration just under an hour for a seven-point assay. For the PLQY assay, the average time between measurements was 6.4 minutes—for a total duration just under an hour for a seven-point assay.

2.4. Parts list

- Gilson GX-241 liquid handler with Verity 4020 syringe pump included
- Gilson 221×1.5×0.4MM probe (27067383)
- Gilson 1 mL syringe (25025343)
- Gilson 338S vial rack
- Fiber optic cables:
  - Broadband UV-Vis to flow cell: OceanOptics QP600-1-SR
  - UV LED to flow cell: OceanOptics QP600-1-XSR
  - Flow cell to spectrometer: OceanOptics QP600-1-SR
- OceanOptics QEPro (QEP04792)
- OceanOptics 5- and 10-micron slits (INTSMA-005 and INTSMA-010)
- OceanOptics deuterium-tungsten halogen light source (DH-2000-BAL)
- OceanOptics 1-cm cuvette holder (No part number; modern equivalent: SQ1-ALL)
  - Only used during calibration
- National Instruments data acquisition device (NIDAQ USB-6001)
- ThorLabs 450 nm LED light source (M450LP2)
- ThorLabs 365 nm LED light source (M365LP1)
- ThorLabs LED driver (M00550118)
- Kimax Kimble 100 mL bottles (14395)
- FisherBrand 1.5 mL, 9mm screw thread vials
- Lenovo ThinkStation (i7-9700T processor, 16 GM RAM, Windows 11 v23H2 64-bit)
- Custom Flow cell (see below)
  - ThorLabs HASMA SMA Bulkhead adapter (×3)
- Custom Vial rack cover (see below)

2.5. Flow cell

The flow cell (**Schematic 1**) is an aluminum prism (approximately a 1-in cube). The flow cell comprises a 1.68-mm diameter through-hole for the tubing and three 5.6-mm holes (roughly 7-mm deep) which are tapped for ¼”-36 threads (to receive a ThorLabs HASMA SMA Bulkhead adapter which in turn binds the SMA 905-terminated fiber optic cables). Centered on these holes, 500-micron holes are machined through the center of the flow cell. An option partial hole can be machined on the bottom of the flow cell (and tapped) to mount the flow cell on a stand. Roblonski’s flow cell is held in place with double-sided adhesive foam tape. A *.STEP file of the part can be found in the GitHub repository in the “Custom Parts” folder: “RoblonskiFlowcell.step”. To shield

the flow cell from ambient light, the tubing on either end of the flow cell was manually covered in aluminum foil. The broadband UV-Vis light source and spectrometer are connected colinearly whereas the excitation light source is connected perpendicularly to the spectrometer.

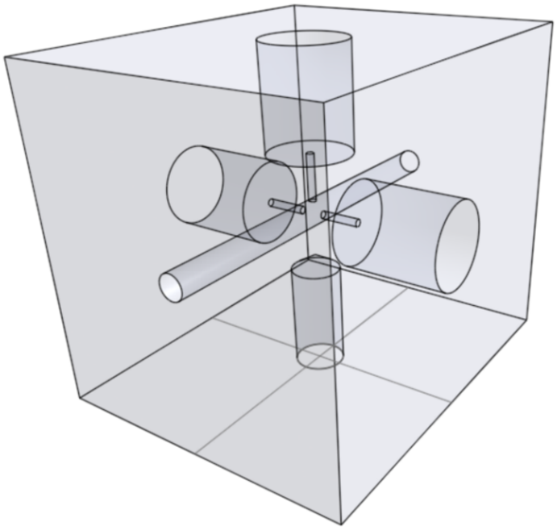

Schematic 1. Translucent, perspective view of the flow cell.

2.6. Vial rack cover

The vial rack cover (**Schematic 2**) was 3D printed using PCTG (1.23 specific gravity) with a 0.8-mm nozzle and comprises 7.3-mm diameter holes spaced 18 mm apart (across the four columns) and 13.73 mm apart (across the 16 rows). The part overall measures 184.53 × 60.42 × 5.72 mm. The corner holes are located with a 10-mm offset in the x and y axes from the corners of the cover. The holes are sunk 1.63 mm into 12.13-mm square recesses to locate vials. A 2.45-mm deep and 2-mm thick lip runs along the perimeter of the cover to further assist in locating the cover on top of the vial rack. A *.STEP file of the part can be found in the GitHub repository in the “Custom Parts” folder: “RoblonskiVialRackCover.step”.

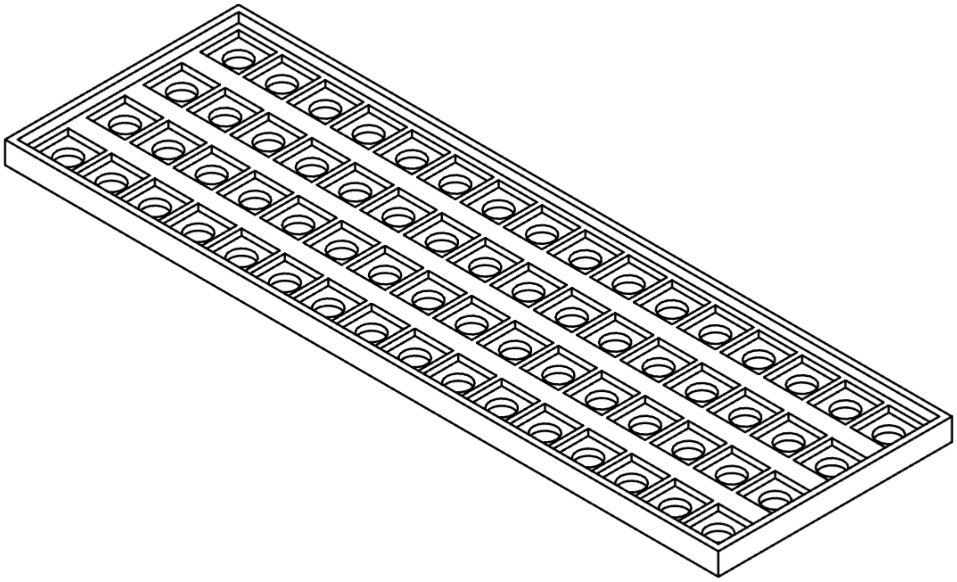

Schematic 2. Upside-down, isometric view of the vial rack cover used to weigh down vials so that the liquid handler’s needle would not pick up vials with foil septa.

3. Platform characterization

3.1. Pipetting

To assess pipetting accuracy and precision, a gravimetric study compared the specified (nominal) volumes aspirated and dispensed by the platform with the realized (actual) volumes. In this study, various volumes of solvent (acetonitrile) were transferred from one vial to another, and the user then massed the latter vial (see below for the full procedure). Using the density of acetonitrile, a calibration curve from nominal to actual volumes was created (**Figure S2**). The uncertainty of this joint aspirate-dispense operation was conservatively used as the estimate for individual aspiration and dispensing operations in the study. Based on the relative uncertainty of actual volumes, the minimum volume required for 5% uncertainty for the sum of three aliquots is 7 µL, which enforces a minimum droplet volume of 21 µL for a three-point SV analysis. In practice, a larger volume (50 µL) is used to reduce uncertainty from needle-liquid-air interactions when sampling multiple vials. This study also found that acetonitrile evaporates at roughly 0.25 µL/min from the 1.5-mL vial used.

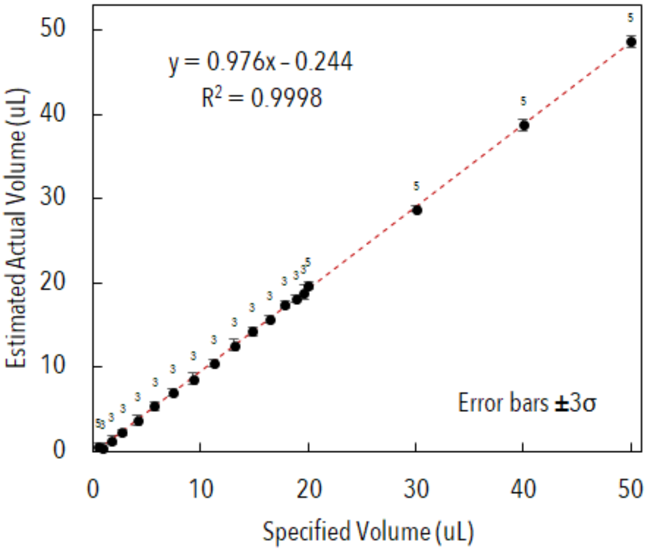

Figure S2. Calibration curve relating the actual to nominal volumes of acetonitrile. The number of replicate measurements at each volume is reported above each data point; replicate values are reported as the average with error bars proportional to the estimated sample standard deviation. Least-squares fit the calibration curve (dotted red line) to all 67 data points.

This pipetting accuracy and precision study used two vials, a source vial and a test vial and assumed a density of 0.784 mg/µL for acetonitrile. Four batches of experiments were performed. The "small volume" study used 3 replicates each for 19.6, 18.8, 17.8, 16.4, 14.8, 13.1, 11.2, 9.3, 7.4, 5.7, 4.1, 2.7, 1.7, and 0.9 µL. The "large volume" study used 5 replicates each for 20, 30, 40, and 50 µL. The "minimal volume" study used 5 replicates of 0.5 µL. Finally, a "losses" study used 7 replicates of a 0 µL transfer. The losses study was used to estimate evaporation and was used to correct the values of the previous 67 measurements. The time between massings was about 4 minutes, and on average, 0.9 ±0.2 µL of acetonitrile was lost. Using the volume and replication parameters of these four studies, the following steps were performed:

1. A source and test vial were loaded onto the liquid handler
2. The source vial was filled to about 1.5 mL, the test vial to about 0.5 mL
3. The test vial was massed
4. A specified volume was transferred from the source vial to the test vial using the liquid handler
    a. Transfers used a wet dispense (needle contacts the fluid) with a tip-touch (when exiting, the tip of the needle is tapped against the lip of the vial)
5. The test vial was massed
6. This was repeated a specific number of times for each volume
    a. When the volume of the test vial exceeded 1.5 mL, 1 mL was manually transferred to the source vial and the test vial was reweighed

3.2. Effective pathlength

To determine the effective pathlengths for a cylindrical tube with a nominal inner diameter of 0.040 inches (1/16" outer diameter), an indirect measurement using Methylene blue as a standard was performed. The flow cell was replaced with a cuvette holder, and three measurements of three independently prepared standard solutions were performed to determine the extinction coefficient of Methylene blue (3.44×10^4 OD cm^{-1} M^{-1} at 652 nm). The flow cell was reconnected and five samples of known concentration were sampled. Using the measured absorbance and empirical extinction coefficient, the pathlength was determined to be either 0.045 ±0.004 inches (using the wavelength at max absorbance: 652 ±2 nm) or 0.039 ±0.002 inches (inverse square uncertainty–weighted average over the entire spectrum). Given the importance of pathlength, a second study was performed to verify the effective pathlength of the flow cell.

To determine the effective optical pathlength of the fluorinated ethylene propylene (FEP) tubing in the automated liquid handling platform, we employed a modified Beer–Lambert approach using zinc(II) meso-tetraphenylporphyrin (ZnTPP) in acetonitrile as the chromophore. Six ZnTPP solutions were independently prepared: three at 4 μM and three at 7 μM. Each solution was measured using quartz cuvettes with 1- and 2-mm pathlengths.

Absorbance spectra were recorded using a DH-2000 BAL deuterium-halogen light source (Ocean Optics) and a QE Pro spectrometer (Ocean Optics), with both components fiber-coupled to a cuvette holder. For each solution, absorbance at the Soret band maximum of ZnTPP (λ_{max} = 420 nm) was recorded in both cuvettes. These measurements provided the basis for determining absorbance as a function of known pathlength.

The same six ZnTPP solutions were then introduced into the FEP tuning in the automated platform and measured under identical optical conditions. The absorbance at 420 nm was again recorded, now using the FEP tubing as the optical path. All data analysis, baseline correction, and plotting were performed using Origin 2023b.

To determine the effective optical pathlength of the FEP tubing, absorbance data obtained from the cuvette measurements were plotted against the known pathlengths (1 and 2 mm) for each solution. The prototypical Beer–Lambert law,

$$A_\lambda = \varepsilon_\lambda \, C \, l \qquad (1)$$

(where A_λ is the absorbance at wavelength λ, ε_λ is the molar extinction coefficient at wavelength λ, C is concentration, and l is the pathlength), was rearranged into a linear form with respect to pathlength:

$$l = (\varepsilon_{420} C)^{-1} A_{420} \ . \tag{2}$$

Using this linear form, a linear regression was performed on the absorbance *vs.* pathlength data for each of the six solutions, yielding a slope equivalent to the inverse of $\varepsilon_{420}C$. This relation was then applied to the measurements taken in the FEP tubing to yield experimental values for the FEP tube's effective pathlength. The effective pathlength and its uncertainty are reported as the average and sample standard deviation of these six measurements. **Figure S3** presents the absorbance spectra recorded in both the cuvettes and FEP tubing, along with representative linear regressions of absorbance vs. cuvette pathlength.

Table S3 summarizes the calibration and measurement of the flow cell pathlength study. This approach yielded an estimated pathlength of 0.102 ±0.004 cm (0.039 inches). Given the agreement between this approach and the previous inverse square uncertainty–weighted average approach, a value of 0.1 cm is used for the effective pathlength of the FEP tube in the flow cell.

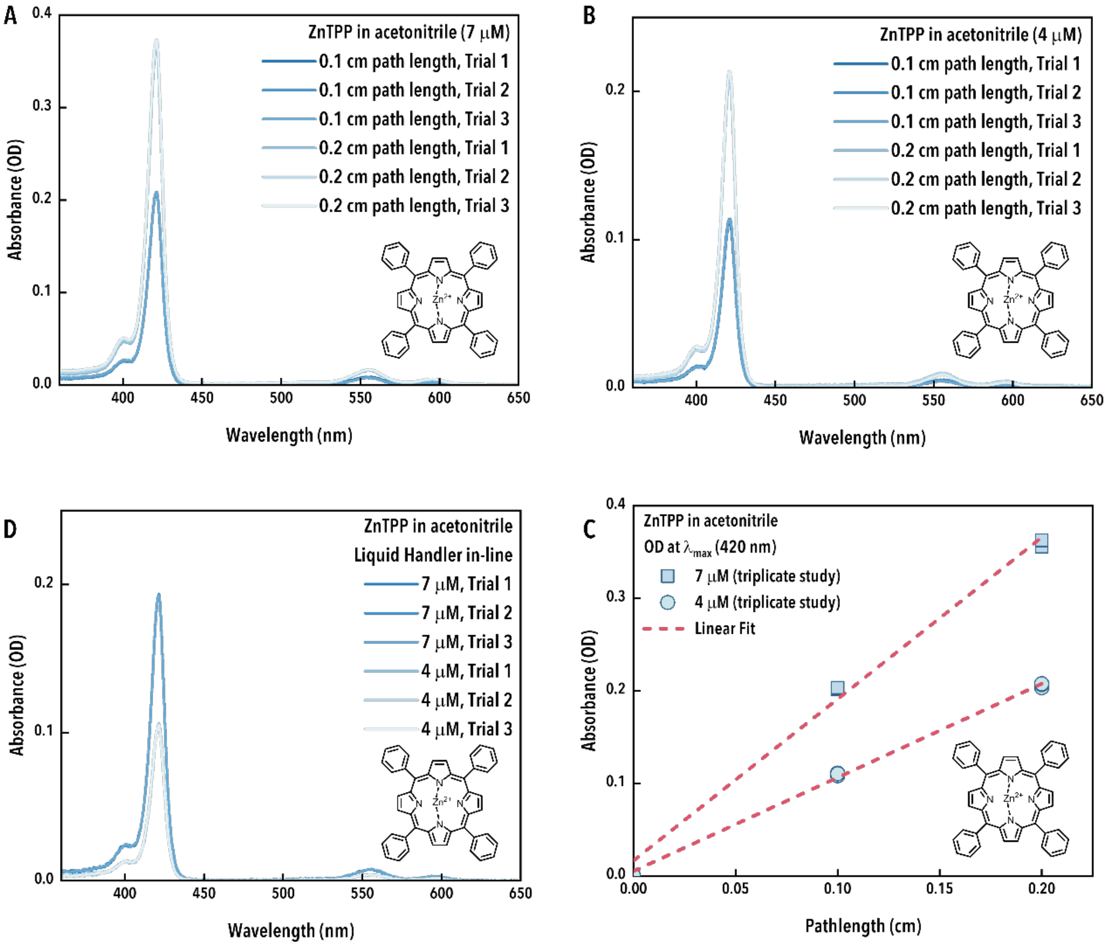

Figure S3. Determination of optical pathlength using zinc(II) meso-tetraphenylporphyrin (ZnTPP) in acetonitrile. Absorbance spectra of ZnTPP at 7 μM (**A**) and 4 μM (**B**) recorded in quartz cuvettes with 1 mm and 2 mm pathlengths across three independently prepared replicates. (**C**) Absorbance spectra of the same six solutions measured through the FEP tubing in the automated liquid handling platform. (**D**) Linear plots of absorbance at 420 nm vs. cuvette pathlength for each solution; slopes from these fits (εC) were used to calculate the effective optical pathlength of the FEP tubing using the Beer–Lambert Law.

Table S3. Summary pathlength calibration and measurement studies.

		Calibration				Measurement	
**Trial**	***C*** [a] **(μM)**	**Cuvette *l* (cm)**	**Cuvette A_{420} (mOD)**	**ε*C*** [b] **(mOD/cm)**	**R^2**	**Tube A_{420} (mOD)**	**Tube *l* (cm)**
1	7	0.1	202	1,747	0.996	183.9	0.1053
		0.2	356				
2		0.1	202			184.2	0.1055
		0.2	362				
3		0.1	204			183.5	0.1050
		0.2	363				
4	4	0.1	108	1,013	0.999	100.0	0.0988
		0.2	204				
5		0.1	111			99.7	0.0984
		0.2	207				
6		0.1	110			100.4	0.0991
		0.2	208				

Concentration, *C*; pathlength, *l*; absorbance at wavelength, A_λ; molar extinction coefficient, *ε*; Square Pearson correlation, R^2.

[a] Zinc(II) meso-tetraphenylporphyrin in acetonitrile.

[b] Slopes are fit with an additional hypothetical data point at the origin (no absorbance at no pathlength).

3.3. Cross-contamination

With these calibrations completed, the cross-contamination of sampling multiple vials in series was investigated. Contamination was measured by having the platform perform a mock SV analysis, in which the sensitizer and quencher vials were filled with Methylene blue dye, and the diluent with acetonitrile. Periodically, the diluent vial would be sampled directly and the dye concentration measured. The gradual increase in absorbance was then fit against the number of interactions (the number of times a contaminated needle entered the vial).

For this study, several contamination mitigation strategies were investigated. In the first strategy, the needle is washed between each sample (an air gap is taken, the needle is moved through a wash bath of water, and then the air gap is ejected). This method ensures the needle is clean before entering the next vial. In the second approach, the needle is poked into a wad of tissue (Kimwipe) between each sample. This method avoids additional wetting of the needle tip during washing. In the third method, the vials are equipped with thin septa (tinfoil), and no washing protocol is used. This method assumes the foil can prevent any droplets from forming on the outside of the needle. The fourth method is the same as the third except that the washing protocol from the first method is also used between vials.

After applying corrections for the changing volume (since volume is taken for the samples and as evaporation is not negligible over the duration of the study), the optimal strategy is the third method (septa and no washing) (**Figure S4**). This is likely due to water infiltrating the needle and not being sufficiently expelled when clearing the airgap. The optimal strategy may also depend on the wettability of the solvent on the needle, so this result may not generalize to different solvent systems.

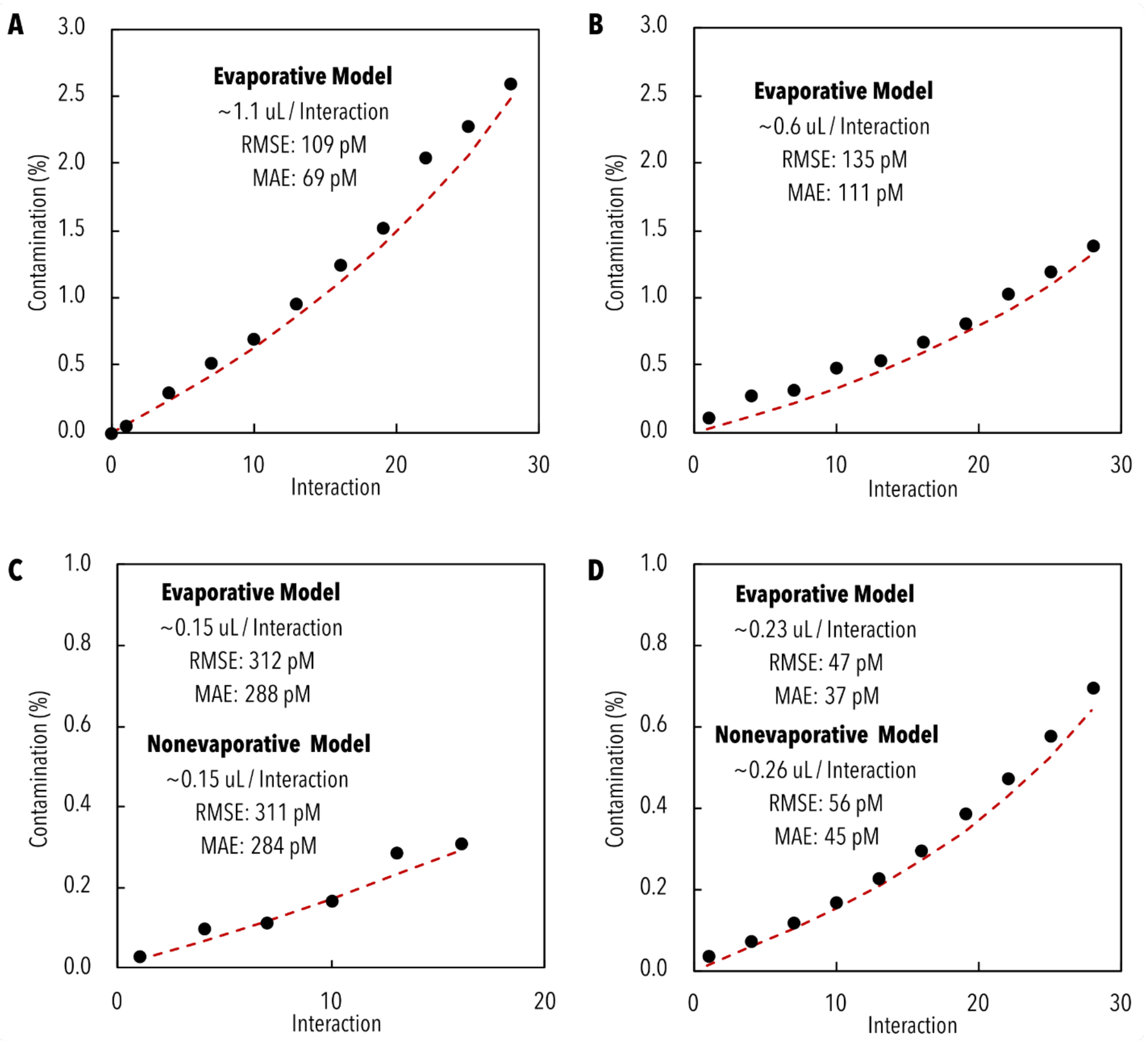

Figure S4. Estimated contamination for Methods 1–4. Percent contamination determined as the concentration of dye in the test vial normalized by the concentration of the source vial. (**A**) Needle washed between each sample, (**B**) Needle treated with absorbent material between each sample, (**C**) Thin septa and no washing, and (**D**) Thin septa and washing between each sample.

3.4. Locality

As the flow cell moves with the arm, the fiber optics and fluid line are moved as well. To ensure this did not affect the results, the coefficient of variation (CV) for the excitation peak (in pure acetonitrile with the light source intensity reduced to not saturate the detector) and fluorescence peak (for a $Ru(bpy)_3(PF_6)_2$ sample) were measured after having the liquid handler jump between random XY coordinates (where X ∈ [5, 160] and Y ∈ [5, 240]; the platform bounds are 1–162 for X and 1–249 for Y) and perform the needle washing motion (a large Z-motion). The CVs for excitation consistency and fluorescence consistency were less than 0.65% (425–475 nm) and 1.5% (550–750 nm), respectively (**Figure S5**).

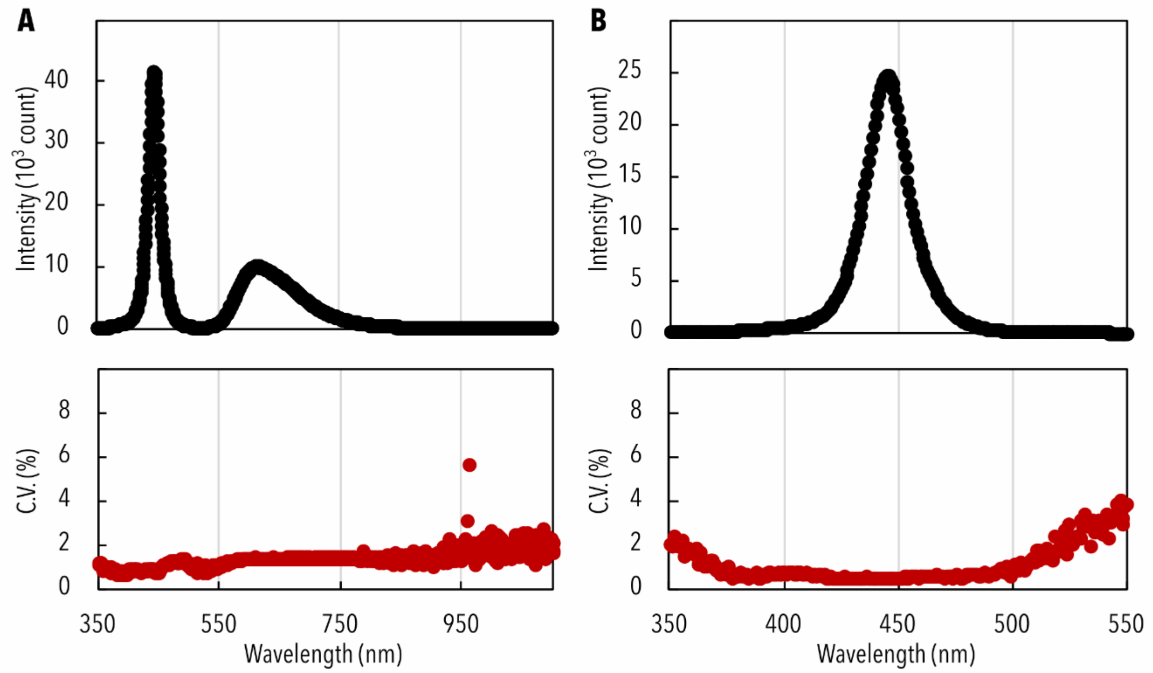

Figure S5. The consistency of spectrometer signal as location is varied. (**A**) The average PL signal and observed coefficient of variation (CV) over 28 samples. (**B**) The average incident light intensity and observed CV over 27 samples.

3.5. Mixing

Proper mixing is necessary to ensure that each droplet is homogenous and that the measured signal corresponds to the assumed composition of the droplet. Mixing is achieved in the fluid line by repeatedly aspirating and dispensing.

Numerous factors can influence mixing (number of iterations, displacement and rate of mixing, the size of the droplet). Of these parameters, it was found that the size of the droplet has the greatest influence on the performance of mixing—this creates a tradeoff between larger droplets minimizing pipetting error but reducing the quality of mixing. The remaining parameters showed little influence on mixing for this system. Given the limited effect of the number of iterations on mixing but its great effect on the duration (and thus throughput) of the assay, the number of mixing iterations was fixed at three iterations. Displacement was set to 300% (*e.g.*, a 10-µL droplet would be moved 30 µL back and forth) based on the heuristic that 200% displacement is required for inversion of the droplet in small droplets[4,5]. The rate of mixing was chosen to be 4 mL/min for the Stern–Volmer study and 1 mL/min for the Beer–Lambert and relative PLQY studies.

4. Stern–Volmer study

4.1. Materials and chemicals

Ferrocene (Sigma-Aldrich) was purified by bulk sublimation before use. Decamethylferrocene and methyl 4-nitrobenzoate (Thermo Scientific), 1,1-dimethylferrocene and acetylferrocene (Strem Chemicals), benzoylferrocene, 3-nitrobenzaldehyde, 4-nitrobenzaldehyde, and acridine (TCI), anthracene (Sigma-Aldrich), and pyrene (Acros Organics) were all used as received. Tris(2,2′-bipyridine)ruthenium(II) hexafluorophosphate ($Ru(bpy)_3(PF_6)_2$) was synthesized in-house following a reported procedure[6]. Spectroscopic grade acetonitrile was obtained from Macron Fine Chemicals and used without further purification.

4.2. Fluorescence lifetime measurement

Fluorescence lifetime measurement of $Ru(bpy)_3(PF_6)_2$ in air-equilibrated acetonitrile was performed using a Mini-tau time-correlated single-photon counting system (Edinburgh Instruments). Excitation was provided by a pulsed 405 nm diode laser. Sample was prepared in a standard 1 cm^2 quartz cuvette with a concentration of 0.067 mM. The emission decay was analyzed by fitting data to a single-exponential model using Origin2023b software. The resulting fluorescence lifetime was determined to be 160 ns. Full decay profile and fitting details are provided in **Figure S6**.

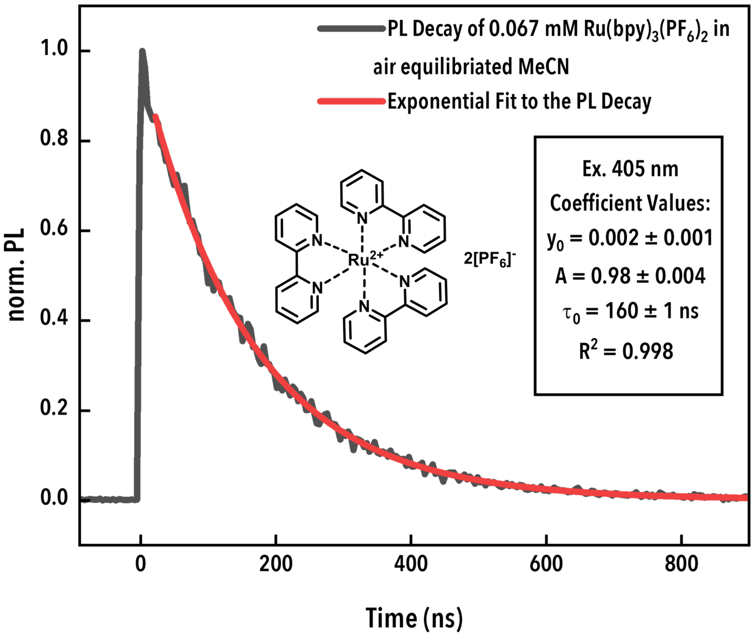

Figure S6. Time-resolved photoluminescence (PL) decay of $Ru(bpy)_3(PF_6)_2$ in air-equilibrated acetonitrile (black line). The red line represents the single-exponential fit to the decay data.

4.3. Manual Stern–Volmer analysis

All quenching experiments were carried out in acetonitrile under air-equilibrated conditions. A fixed concentration of $Ru(bpy)_3(PF_6)_2$ (0.167 mM) was used, and steady-state fluorescence emission spectra were recorded following incremental additions of various quenchers (details

provided in **Table S4**). Samples were excited at 450 nm, and emission was monitored at the $Ru(bpy)_3(PF_6)_2$ maximum (~610 nm) using a Cary Eclipse fluorescence spectrophotometer (Agilent Technologies). Solvent blank subtraction was applied to all spectra to correct for background signals. Measurements were performed at room temperature using a standard 1 cm^2 quartz cuvette.

4.4. Automated Stern–Volmer analysis

All quenching experiments were carried out in acetonitrile under air-equilibrated conditions. A fixed concentration of $Ru(bpy)_3(PF_6)_2$ (5.0 mM) was used, and steady-state fluorescence emission spectra were recorded for each sample following the procedure outlined below. Samples were excited at 450 nm using a light-emitting diode (LED) light source (ThorLabs), and emission was monitored at the $Ru(bpy)_3(PF_6)_2$ maximum (~610 nm) using a QE Pro spectrometer (Ocean Optics). Light and dark references for absorbance calculation were performed on the system solvent (acetonitrile) acting as a solvent blank subtraction for the sample (in acetonitrile). Measurements were performed at room temperature in the microfluidic flow cell.

All photoluminescence measurements in this study (and the quantum yield study, see Section 6) were acquired using an Ocean Optics QE Pro spectrometer equipped with a back-thinned charge-coupled device detector. The detector exhibits a broad and relatively smooth quantum efficiency across the visible region, which encompasses the emission bands of all fluorophores studied here. Under these conditions, uncorrected PL spectra reliably capture relative spectral features and intensity trends when measurements are performed under consistent optical geometry and acquisition settings. Wavelength-dependent detector response corrections were therefore not applied in this work, as the PL data are used comparatively rather than as absolute spectral radiance measurements. Such corrections would be required for absolute intensity calibration, cross-platform spectral comparison, or measurements extending into regions of rapidly varying detector sensitivity (e.g., deep-UV or near-IR).

The following protocol describes the liquid handler automation of the Stern–Volmer analysis. Flow rates are 1 mL/min unless otherwise stated; the washing sub-protocol is subsequently detailed.

Stern–Volmer Automated Protocol:

i. The user is prompted for a priming volume at run-time (or may hard-code a value prior to running). Typical values include 200 µL (upon immediate restart) and 1400 µL (to clear bubbles in the line at startup).
ii. In partitions of at most 400 µL, the liquid handler withdraws from the reservoir, then dispenses through the needle to waste at 20 mL/min (except the last partition, which uses 1 mL/min).
iii. The liquid handler aspirates a 20 µL air gap.
[
iv. The liquid handler aspirates 10 µL from the Catalyst vial.
v. It aspirates 40 µL from the <u>Diluent</u> vial then takes a 10 µL air gap.
vi. It aspirates and dispenses 150 µL of air at 4 mL/min three times to mix the droplet.
vii. It then slowly aspirates 165.5 µL (this value is automatically calculated based on the size of the droplet and air gaps and on the measured volumes of the system lines) of air to center the droplet in the flow cell.

viii. The spectrometer then collects 5 PL spectra, integrating for 2 seconds each, resting 0.1 seconds between scans—the average is then reported.

ix. The liquid handler then dispenses everything to waste.

x. It flushes the needle with 200 µL of acetonitrile, washes the outside of the needle twice, then flushes the needle twice with 200 µL of acetonitrile (using a 20 mL/min flow rate).
]

xi. The same steps (**iv–x**) are repeated using <u>Quencher</u> instead of <u>Diluent</u>.

xii. The following steps (**xiii–xx**) are repeated varying the volumes of Diluent and Quencher in a random order (<u>*X*</u>: 10.0, 16.67, 23.33, and 30; performed in a randomized order each time):
[

xiii. The liquid handler samples 10 µL from the Catalyst vial.

xiv. It samples <u>*X*</u> µL from the Quencher vial.

xv. It samples <u>40-*X*</u> µL from the Diluent vial then takes a 10-µL airgap

xvi. It aspirates and dispenses 150 µL of air at 4 mL/min three times to mix the droplet.

xvii. It then slowly aspirates 170.5 µL of air to center the droplet in the flow cell.

xviii. The spectrometer then collects 5 PL spectra, integrating for 2 seconds each, resting 0.1 seconds between them—the average is then reported.

xix. The liquid handler then dispenses everything to waste.

xx. It flushes the needle with 200 µL of acetonitrile, washes the outside of the needle twice, then flushes the needle twice with 200 µL of acetonitrile (using a 20 mL/min flow rate).
]

xxi. Preliminary data processing is performed. If the R^2 value is less than 0.97 or if the y-intercept is not in the range 0.9–1.1, then the no-quencher sample (**iv-x**, using <u>Diluent</u>) and the "most surprising" (ignoring the no-quencher sample) are repeated—see "Self-checking" below.

xxii. It flushes the needle thrice with 200 µL of acetonitrile and returns the needle to the home position.

External washing protocol:

i. The liquid handler aspirates a 10-µL air gap.
[

ii. It submerges the needle in a wash bath (water)

iii. It moves the needle laterally to contact the rim of the bath

iv. It drags the needle up the wall, then returns the needle to a position above the center of the bath.
]

v. The same steps (**ii–iv**) are repeated a specified number of times (typically twice).

vi. It dispenses 10 µL (the air gap) over waste.

Self-checking:

Under the assumptions of the Stern–Volmer model for quenching, the relationship between I_0/I and quencher concentration should be linear with a y-intercept at 1. Given this supposition, the platform automatically checks the Pearson's R^2 value (to ensure data is a line) and the y-intercept (to ensure the physical assumptions of the model hold) for each catalyst-quencher K_{SV} value. The

threshold of 0.97 for the R^2 value was chosen based on similar R^2 values reported in the literature, and the 10% error for the y-intercept was chosen arbitrarily.

The slope (K_{SV}) and intercept are determined by simple linear regression (see simple_linear_regression.py in the associated code repository). During this calculation an estimate of the uncertainty in the predicted response for a new datum (not the uncertainty in the mean response) can be calculated based on the previous n observations:

$$\hat{s}_{\hat{y}(x_t)} = \sqrt{\left(\frac{\sum_i(\hat{y}(x_i) - y(x_i))^2}{n-2}\right)\left(1 + \frac{1}{n} + \frac{(x_t-\bar{x})^2}{\sum_i(x_i^2)}\right)} \tag{3}$$

where x_t is a new quencher concentration and $\hat{y}(x)$ is the I_0/I value estimated at x by linear regression fit to all data except (x_t, $y(x_t)$). All sums are over the n data which do not include (x_t, $y(x_t)$).

The surprise, z, of a new datum is proportional to

$$z(x_t) \propto \frac{|y(x_t)-\hat{y}(x_t)|}{\hat{s}_{\hat{y}(x_t)}} \tag{4}$$

Complete statistical analysis would then require this be scaled by some factor depending on the assumption of the underlying distribution of the population and noise (such as a Student's t-value); however, as the checking protocol only considers the rank order of surprises, the factor can be ignored.

4.5. Calculation of quenching constants

Stern–Volmer quenching constants (K_{SV}) were determined using the fluorescence intensity method. Photoluminescence (PL) intensities in the presence (I) and absence (I_0) of quencher were obtained from steady-state emission spectra. A Stern–Volmer plot was constructed by plotting the ratio I_0/I on the y-axis against the quencher concentration [Q] on the x-axis. Data was fit to the linear Stern–Volmer equation:

$$\frac{I_0}{I} = 1 + K_{SV}[Q] \tag{5}$$

The slope of the linear fit provided the Stern–Volmer constant K_{SV} for each quencher.
The bimolecular quenching rate constant k_q was calculated using:

$$k_q = \frac{K_{SV}}{\tau_0} \tag{6}$$

where τ_0 is the PL lifetime of $Ru(bpy)_3(PF_6)_2$ in the absence of quencher, determined to be 160 ns under air-equilibrated conditions in acetonitrile.
The associated error in k_q was estimated using standard error propagation:

$$\Delta k_q = k_q\sqrt{\left(\frac{\Delta K_{SV}}{K_{SV}}\right)^2 + \left(\frac{\Delta \tau_0}{\tau_0}\right)^2} \tag{7}$$

Where ΔK_{SV} is the standard error of the slope from the Stern–Volmer plot, and $\Delta\tau_0$ is the standard error from the exponential fit of the measured fluorescence decay. All reported average k_q values include these propagated uncertainties. All data analysis and plotting were performed using Origin 2023b. Experimental data are reported in **Figure S7** and in **Table S4** for the manual study and **Table S5** for the automated study.

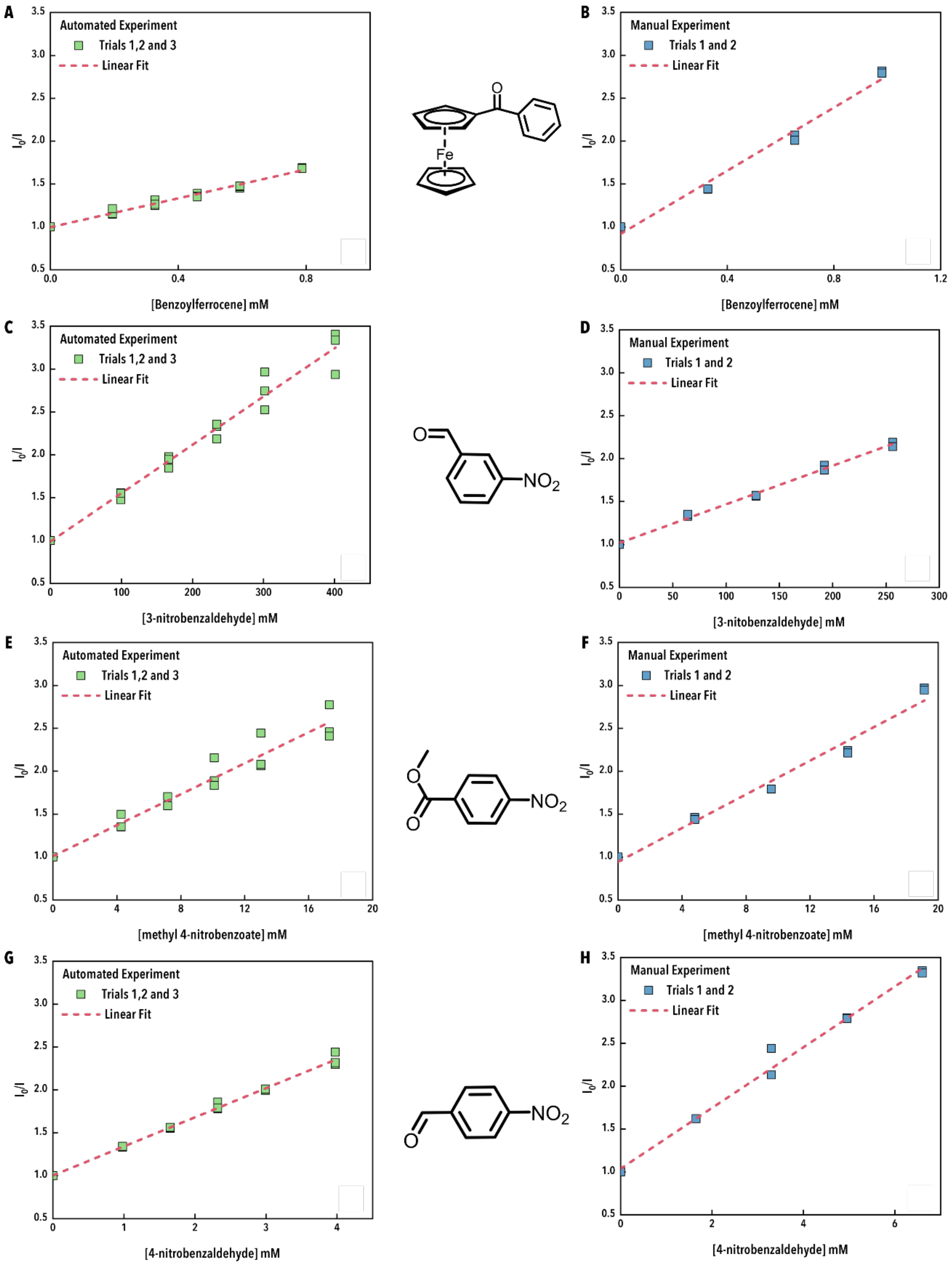

A
Automated Experiment
Trials 1,2 and 3
Linear Fit
I0/I
[Benzoylferrocene] mM
B
Manual Experiment
Trials 1 and 2
Linear Fit
I0/I
[Benzoylferrocene] mM
Fe
O
C
Automated Experiment
Trials 1,2 and 3
Linear Fit
I0/I
[3-nitrobenzaldehyde] mM
D
Manual Experiment
Trials 1 and 2
Linear Fit
I0/I
[3-nitobenzaldehyde] mM
NO2
E
Automated Experiment
Trials 1,2 and 3
Linear Fit
I0/I
[methyl 4-nitrobenzoate] mM
F
Manual Experiment
Trials 1 and 2
Linear Fit
I0/I
[methyl 4-nitrobenzoate] mM
G
Automated Experiment
Trials 1,2 and 3
Linear Fit
I0/I
[4-nitrobenzaldehyde] mM
H
Manual Experiment
Trials 1 and 2
Linear Fit
I0/I
[4-nitrobenzaldehyde] mM

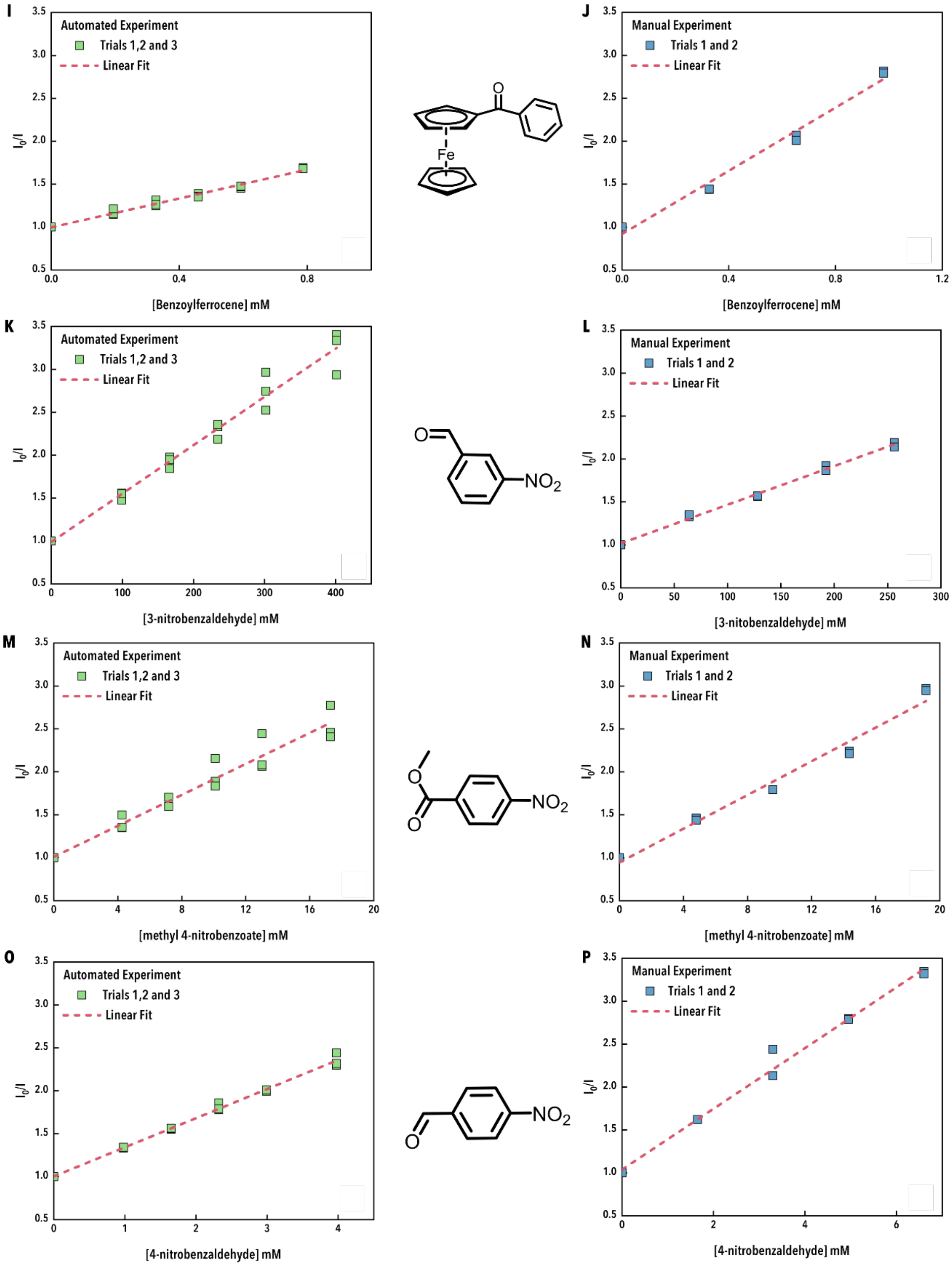
I
Automated Experiment
Trials 1,2 and 3
Linear Fit
I_0/I
[Benzoylferrocene] mM
Fe
O
J
Manual Experiment
Trials 1 and 2
Linear Fit
I_0/I
[Benzoylferrocene] mM
K
Automated Experiment
Trials 1,2 and 3
Linear Fit
I_0/I
[3-nitrobenzaldehyde] mM
O
NO_2
L
Manual Experiment
Trials 1 and 2
Linear Fit
I_0/I
[3-nitobenzaldehyde] mM
M
Automated Experiment
Trials 1,2 and 3
Linear Fit
I_0/I
[methyl 4-nitrobenzoate] mM
O
O
NO_2
N
Manual Experiment
Trials 1 and 2
Linear Fit
I_0/I
[methyl 4-nitrobenzoate] mM
O
Automated Experiment
Trials 1,2 and 3
Linear Fit
I_0/I
[4-nitrobenzaldehyde] mM
O
NO_2
P
Manual Experiment
Trials 1 and 2
Linear Fit
I_0/I
[4-nitrobenzaldehyde] mM

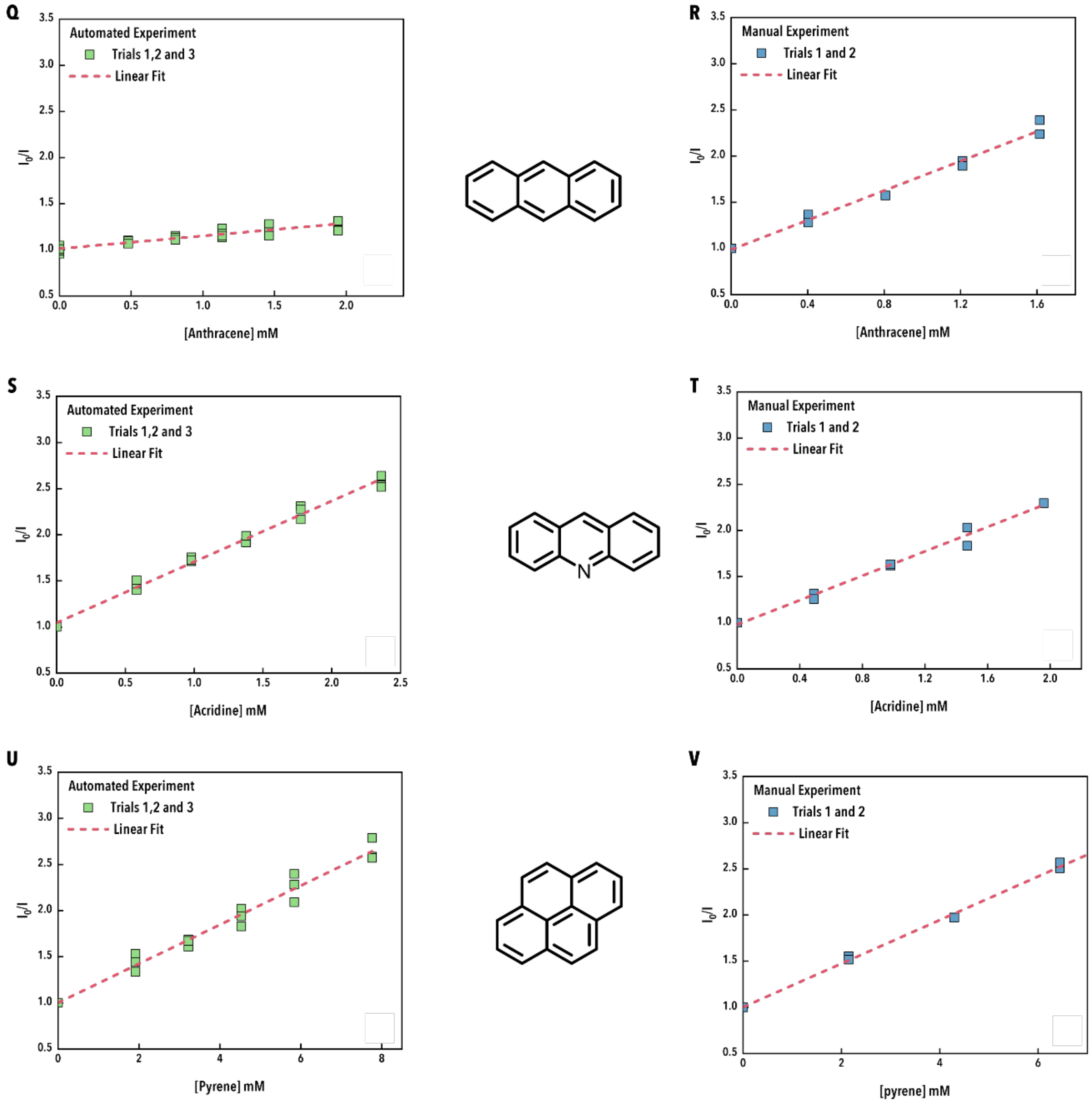

Figure S7. Automated (green, left) and manual (blue, right) Stern–Volmer plots for photoluminescence quenching of $Ru(bpy)_3(PF_6)_2$ by various quenchers in acetonitrile under air-equilibrated conditions at room temperature: (**A**, **B**) ferrocene, (**C**, **D**) decamethylferrocene, (**E**, **F**) 1,1'-dimethylferrocene, (**G**, **H**) acetylferrocene, (**I**, **J**) benzoylferrocene, (**K**, **L**) 3-nitrobenzaldehyde, (**M**, **N**) methyl 4-nitrobenzoate, (**O**, **P**) 4-nitrobenzaldehyde, (**Q**, **R**) anthracene, (**S**, **T**) acridine, and (**U**, **V**) pyrene.

Table S4. (Manual) Data summary for the manual experiments depicted in **Figure S7**. Stern–Volmer analysis of $Ru(bpy)_3(PF_6)_2$ quenching in air-equilibrated acetonitrile at room temperature. Duplicate experiments are shown separately.

Quencher	[Q] (mM)	I_0/I Trial 1	I_0/I Trial 2	Slope K_{SV} (M^{-1})	Intercept	R^2	k_q (M^{-1} s^{-1})
Ferrocene	0.000	1.000	1.000	1437 ±20	0.98 ±0.02	0.998	9.1 $\times 10^9$ ±0.1 $\times 10^9$
	0.333	1.440	1.445				
	0.667	1.931	1.905				
	1.000	2.406	2.349				
	1.333	2.947	2.908				
Decamethyl-ferrocene	0.000	1.000	1.000	2300 ±60	0.98 ±0.02	0.994	1.4 $\times 10^{10}$ ±0.04 $\times 10^{10}$
	0.130	1.329	1.232				
	0.261	1.584	1.526				
	0.391	1.869	1.861				
	0.521	2.157	2.137				
	0.652	2.552	–				
1,1-Dimethyl-ferrocene	0.000	1.000	1.000	1900 ±20	1.00 ±0.05	0.930	1.2 $\times 10^{10}$ ±0.01 $\times 10^{10}$
	0.122	1.157	1.236				
	0.243	1.412	1.575				
	0.365	1.554	1.821				
	0.487	1.799	2.018				
Acetyl-ferrocene	0.000	1.000	1.000	1340 ±70	0.97 ±0.06	0.977	8.4 $\times 10^9$ ±0.4 $\times 10^9$
	0.332	1.308	1.374				
	0.663	1.786	2.059				
	0.995	2.136	2.324				
	1.327	2.757	2.801				
Benzoyl-ferrocene	0.000	1.000	1.000	1840 ±90	0.92 ±0.06	0.966	1.3 $\times 10^{10}$ 0.06 $\times 10^{10}$
	0.327	1.438	1.444				
	0.654	2.072	2.009				
	0.980	2.818	2.793				
	1.307	3.918	–				
3-Nitrobenz-aldehyde	0.000	1.000	1.000	5.0 ±0.1	1.02 ±0.02	0.995	2.80 $\times 10^7$ ±0.06 $\times 10^7$
	64.044	1.330	1.352				
	128.088	1.563	1.573				
	192.132	1.922	1.866				
	256.176	2.192	2.141				
Methyl 4-nitro-benzoate	0.000	1.000	1.000	98 ±5	0.95 ±0.06	0.979	6.1 $\times 10^8$ ±0.3 $\times 10^8$
	4.786	1.460	1.436				
	9.571	1.794	1.792				
	14.357	2.239	2.213				
	19.142	2.971	2.946				

(**Table S4** continued; Manual)

Quencher	[Q] (mM)	I_0/I Trial 1	I_0/I Trial 2	Slope K_{SV} (M^{-1})	Intercept	R^2	k_q (M^{-1} s^{-1})
4-Nitrobenz-aldehyde	0.000	1.000	1.000	350 ±10	1.04 ±0.05	0.990	2.20×10^9 $\pm 0.06 \times 10^9$
	1.650	1.621	1.622				
	3.300	2.132	2.440				
	4.950	2.797	2.788				
	6.600	3.346	3.318				
Anthracene	0.000	1.000	1.000	800 ±30	0.99 ±0.03	0.988	5.0×10^9 $\pm 0.2 \times 10^9$
	0.403	1.366	1.281				
	0.807	–	1.573				
	1.210	1.948	1.893				
	1.614	2.238	2.389				
Acridine	0.000	1.000	1.000	660 ±20	0.98 ±0.03	0.989	4.1×10^9 $\pm 0.1 \times 10^9$
	0.490	1.314	1.257				
	0.980	1.619	1.634				
	1.469	2.031	1.834				
	1.959	2.296	2.296				
Pyrene	0.000	1.000	1.000	236 ±4	1.00 ±0.02	0.998	1.50×10^9 $\pm 0.03 \times 10^9$
	2.147	1.555	1.516				
	4.294	1.974	1.971				
	6.442	2.507	2.569				
	8.589	3.035	–				

Table S5. (Automated) Data summary for the automated experiments depicted in **Figure S7**. Stern–Volmer analysis of $Ru(bpy)_3(PF_6)_2$ quenching in air-equilibrated acetonitrile at room temperature. Triplicate experiments are shown separately. Automatically repeated measurements are shown on separate lines.

Quencher	[Q] (mM)	I_0/I			Slope K_{SV} (M^{-1})	Intercept	R^2	k_q (M^{-1} s^{-1})
		Trial 1	Trial 2	Trial 3				
Ferrocene	0.000	1.000	1.000	0.999<br>1.001	1.27 ±0.05	1.06 ±0.03	0.988	7.9 ×10^9 ±0.3 ×10^9
	0.297	1.450	1.387	1.520				
	0.500	1.746	1.637	1.811				
	0.703	2.019	1.895	2.121<br>2.132				
	0.906	2.251	2.145	2.221				
	1.204	2.559	2.441	2.591				
Decamethyl-ferrocene	0.000	1.000	1.000	1.000	2.37 ±0.08	1.00 ±0.04	0.991	1.50 ×10^{10} ±0.05 ×10^{10}
	0.197	1.455	1.513	1.498				
	0.332	1.710	1.876	1.761				
	0.467	2.005	2.182	2.069				
	0.602	2.265	2.520	2.359				
	0.801	2.769	3.087	2.941				
1,1'-dimethyl-ferrocene	0.000	1.000	1.000	1.000	1.57 ±0.07	1.02 ±0.05	0.984	9.8 ×10^9 ±0.4 ×10^9
	0.302	1.525	1.425	1.656				
	0.509	1.779	1.723	1.862				
	0.716	2.069	2.034	2.216				
	0.922	2.391	2.339	2.799				
	1.226	2.927	2.783	3.049				
Acetylferrocene	0.000	1.000	1.000	1.000	1.29 ±0.04	1.00 ±0.03	0.991	8.1 ×10^9 ±0.3 ×10^9
	0.295	1.345	1.407	1.399				
	0.498	1.564	1.651	1.649				
	0.700	1.782	1.941	2.016				
	0.902	2.115	2.258	2.196				
	1.199	2.502	2.410	2.680				
Benzoyl-ferrocene	0.000	1.000	1.000	1.000	0.84 ±0.02	1.00 ±0.02	0.993	5.3 ×10^9 ±0.1 ×10^9
	0.194	1.149	1.155	1.213				
	0.327	1.250	1.315	1.262				
	0.459	1.390	1.357	1.352				
	0.592	1.454	1.470	1.477				
	0.787	1.692	1.687	1.682				
3-nitro-benzaldehyde	0.000	1.000	1.000	1.000	0.0060 ±0.0002	0.99 ±0.06	0.986	3.5 ×10^7 ±0.1 ×10^7
	98.895	1.479	1.558	1.555				
	166.518	1.845	1.980	1.948				
	234.131	2.186	2.331	2.359				
	301.754	2.525	2.745	2.968				
	401.148	2.938	3.406	3.337				

(**Table S5** continued; Automated)

Quencher	[Q] (mM)	I_0/I Trial 1	Trial 2	Trial 3	Slope K_{SV} (M^{-1})	Intercept	R^2	k_q (M^{-1} s^{-1})
Methyl-4-nitrobenzoate	0.000	1.000	1.000	1.000	0.090 ±0.005	1.01 ±0.05	0.974	5.6×10^8 $\pm 0.3 \times 10^8$
	4.263	1.349	1.354	1.499				
	7.178	1.596	1.598	1.703				
	10.093	1.889	1.833	2.156				
	13.008	2.064	2.080	2.444				
	17.293	2.459	2.408	2.774				
4-nitro-benzaldehyde	0.000	1.000	1.000	1.000	0.339 ±0.006	1.00 ±0.01	0.997	2.10×10^9 $\pm 0.04 \times 10^9$
	0.980	1.334	1.335	1.343				
	1.650	1.554	1.559	1.565				
	2.320	1.861	1.780	1.792				
	2.990	1.997	1.996	2.011				
	3.975	2.441	2.300	2.320				
Anthracene	0.000	1.000	0.958<br>1.046	0.992<br>1.008	0.14 ±0.01	1.01 ±0.01	0.926	8.6×10^8 $\pm 0.6 \times 10^8$
	0.479	1.101	1.094	1.006				
	0.807	1.154	1.131	1.109				
	1.135	1.190	1.231<br>1.174	1.135<br>1.153				
	1.462	1.281	1.191	1.153				
	1.944	1.306	1.313	1.209				
Acridine	0.000	1.000	1.000	1.000	0.66 ±0.02	1.05 ±0.02	0.995	4.1×10^9 $\pm 0.1 \times 10^9$
	0.582	1.403	1.469	1.509				
	0.980	1.712	1.754	1.725				
	1.377	1.918	1.917	1.989				
	1.775	2.166	2.311	2.276				
	2.360	2.640	2.537	2.519				
Pyrene	0.000	1.000	1.000	1.000	0.212 ±0.008	1.00 ±0.04	0.988	1.30×10^9 $\pm 0.05 \times 10^9$
	1.913	1.441	1.336	1.534				
	3.221	1.687	1.611	1.671				
	4.529	2.020	1.829	1.939				
	5.837	2.282	2.092	2.398				
	7.759	2.787	2.585	2.570				

5. Beer–Lambert study

5.1. Materials and chemicals

ZnTPP and ferrocene were purchased from Sigma-Aldrich. Ferrocene was sublimed in bulk prior to use; all other chemicals were used as received. Zinc(II) phthalocyanine (ZnPc) was obtained from Thermo Fisher Scientific. Rhodamine B was purchased from Acros Organics. Perylene was obtained from Alfa Aesar. Tris(2,2′-bipyridine)ruthenium(II) hexafluorophosphate ($Ru(bpy)_3(PF_6)_2$) was synthesized in-house following a previously reported literature procedure[6]. Spectroscopic grade solvents were purchased as follows: toluene from J.T. Baker, acetonitrile from Macron Fine Chemicals, pyridine from Alfa Aesar, methanol from Supelco (Uvasol®), and cyclohexane from Thermo Fisher Scientific.

5.2. Manual Beer–Lambert study

Stock solutions of each chromophore with known concentration were prepared and serially diluted to obtain multiple solutions of varying concentration. To ensure adherence to Beer–Lambert Law's linear range, sample concentrations were diluted such that absorbance did not exceed 1.0 OD. All samples were measured in standard 1 cm^2 quartz cuvettes at room temperature using air-equilibrated solutions. Absorbance spectra were recorded on a Cary 60 Bio UV–vis spectrophotometer (Agilent Technologies) with baseline and solvent blank corrections applied to all measurements.

5.3. Automated Beer–Lambert study

The following protocol describes the liquid handler automation of the Beer–Lambert analysis. Flow rates are 1 mL/min unless otherwise stated; the external washing protocol is the same as it was in the SV analysis. Absorbance measurements are performed on samples in a manner similar to the Stern–Volmer protocol and use the average of 30 scans integrating over 40 ms each, spaced 0.1 seconds apart. The light and dark references for the absorbance scan are relative to the system fluid (acetonitrile). A measurement of the sample solvent is used to correct for the difference in solvent.

Beer–Lambert Analysis Protocol:

i. The user is prompted for a priming volume at run-time (or may hard-code a value prior to running). Typical values include 200 µL (immediate restart) and 1400 µL (bubbles in the line).
ii. In partitions of at most 400 µL, the liquid handler withdraws from the reservoir, then dispenses through the needle to waste at 20 mL/min (except the last partition, which uses 1 mL/min).
iii. The liquid handler aspirates a 20-µL air gap.
iv. 50 µL of the Diluent vial is sampled and its absorbance is measured to be used as a background.
v. 50 µL of the Source vial is sampled and its absorbance is measured.
vi. If the absorbance is greater than 110 mOD, the Working vial is prepared with a combination of Source vial and Diluent vial aliquots such that the optical density should be 100 mOD (see *Preparing a diluted Working vial* below); otherwise, 250 µL of the Source vial is transferred to the Working vial.
vii. The absorbance of the Working vial's contents is measured using an 83.33 µL (250/3 µL) sample.

[
viii. The liquid handler expels the needle's contents to waste.
ix. The liquid handler transfers 83.33 µL of solvent from the Diluent vial to the Working vial.
x. The liquid handler mixes the Working vial by aspirating and dispensing 175 µL seven times.
xi. The absorbance of the Working vial's contents is measured using an 83.33-µL sample.
]
xii. The previous steps (**viii–xi**) are repeated until there are six diluted sample absorbance spectra and one undiluted sample absorbance spectrum.
xiii. The liquid handler then dispenses everything in the needle to waste.
xiv. It flushes the needle with 200 µL of acetonitrile, washes the outside of the needle twice, then flushes the needle twice with 200 µL of acetonitrile (using a 20 mL/min flow rate).
xv. For multiple analytes, steps **iv–xiv** can be repeated for each analyte.
xvi. The liquid handler flushes the needle thrice with 200 µL of acetonitrile and returns the needle to the home position.

Preparing a diluted Working vial:

i. Using the observed absorbance of the Source vial's sample and assuming a linear response between this point and the origin, the relative amount of Source and Diluent is determined to achieve an optical density of 100 mOD.
ii. This volume is scaled so that the smallest volume is equal to the minimal pipettable volume—10 µL.
iii. If the total volume would be greater than 1 mL, the procedure aborts.
iv. If either aliquot (Source or Diluent) would be greater than 900 µL, the procedure aborts.
v. If the total volume would be less than 250 µL, the volumes are scaled up so that the total volume is 250 µL.
vi. The needle is washed.
vii. The larger of the two aliquots is transferred to the Working vial first.
viii. The needle is washed.
ix. The remaining aliquot is transferred to the Working vial.
x. The mixing displacement, v_d, is determined based on the Working vial's liquid volume, v_w, (a value between 250 and 1000 µL) such that

$$v_d = \min\left(425, \quad 175 + \tfrac{3}{4}(v_w - 250)\right). \tag{8}$$

a. It is possible for v_d to be greater than v_w, this will result in air being aspirated and a portion of the Working vial's contents to be left behind. This, however, is per cycle, as the aspirated contents are returned to the vial before the next mixing iteration and so is not considered an issue.

xi. The number of mixing iterations, n_m, is determined based on the v_d and v_w such that

$$n_m = 3 + \left\lfloor 0.67 + 3\frac{v_w}{v_d} \right\rfloor. \tag{9}$$

xii. The contents of the Working vial are mixed with the needle fixed at the bottom of the vial.
xiii. If the total liquid volume of the vial is greater than 250 μL, the liquid handler transfer the excess volume to waste (*e.g.*, if v_w is 400 μL, then 150 μL is discarded)
xiv. The needle is washed.

5.4. Calculation of molar extinction coefficients

The molar extinction coefficient, ε, was determined by applying the Beer–Lambert Law:

$$A = \varepsilon \cdot C \cdot l \tag{10}$$

where A is the absorbance at the chromophore's absorption maximum (λ_{max}), C is the concentration in molarity (M), and l is the optical pathlength (cm).

For the manual study, absorbance values at λ_{max} were plotted against concentration, and the slope of the linear regression corresponded directly to the molar extinction coefficient ε (M^{-1} cm^{-1}), since $l = 1$ cm.

For the automated study, the molar extinction coefficient was calculated by dividing the slope of the absorbance vs. concentration linear fit by the optical pathlength of the FEP tubing, which was experimentally determined to be 0.1 cm:

$$\varepsilon = \frac{slope}{l} \tag{11}$$

The uncertainty in ε was calculated using error propagation, considering the standard error in the slope (Δ_{slope}) and the uncertainty in the optical pathlength (σ_l):

$$\Delta\varepsilon = \varepsilon \times \sqrt{\left(\frac{\Delta slope}{slope}\right)^2 + \left(\frac{\sigma_l}{l}\right)^2} \tag{12}$$

All data analysis and plotting were performed using Origin 2023b. Experimental data are reported in **Figure S8** and **Figure S9** and in **Table S6** for the manual study and **Table S7** for the automated study.

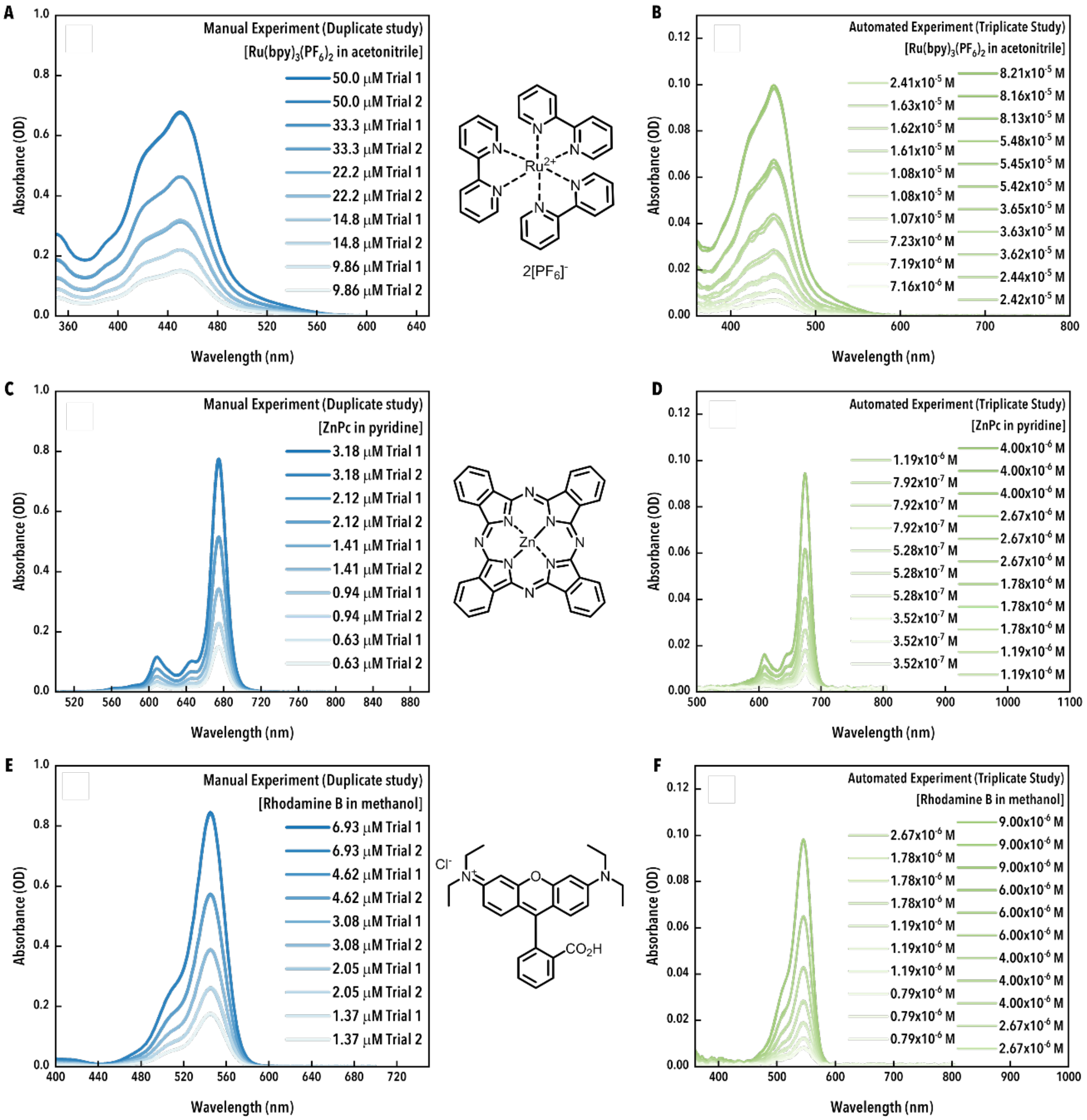
A
Manual Experiment (Duplicate study)
[Ru(bpy)3(PF6)2 in acetonitrile]
50.0 μM Trial 1
50.0 μM Trial 2
33.3 μM Trial 1
33.3 μM Trial 2
22.2 μM Trial 1
22.2 μM Trial 2
14.8 μM Trial 1
14.8 μM Trial 2
9.86 μM Trial 1
9.86 μM Trial 2
Absorbance (OD)
Wavelength (nm)
Ru2+
2[PF6]-
B
Automated Experiment (Triplicate Study)
[Ru(bpy)3(PF6)2 in acetonitrile]
2.41x10-5 M
1.63x10-5 M
1.62x10-5 M
1.61x10-5 M
1.08x10-5 M
1.08x10-5 M
1.07x10-5 M
7.23x10-6 M
7.19x10-6 M
7.16x10-6 M
8.21x10-5 M
8.16x10-5 M
8.13x10-5 M
5.48x10-5 M
5.45x10-5 M
5.42x10-5 M
3.65x10-5 M
3.63x10-5 M
3.62x10-5 M
2.44x10-5 M
2.42x10-5 M
Absorbance (OD)
Wavelength (nm)
C
Manual Experiment (Duplicate study)
[ZnPc in pyridine]
3.18 μM Trial 1
3.18 μM Trial 2
2.12 μM Trial 1
2.12 μM Trial 2
1.41 μM Trial 1
1.41 μM Trial 2
0.94 μM Trial 1
0.94 μM Trial 2
0.63 μM Trial 1
0.63 μM Trial 2
Absorbance (OD)
Wavelength (nm)
Zn
D
Automated Experiment (Triplicate Study)
[ZnPc in pyridine]
1.19x10-6 M
7.92x10-7 M
7.92x10-7 M
7.92x10-7 M
5.28x10-7 M
5.28x10-7 M
5.28x10-7 M
3.52x10-7 M
3.52x10-7 M
3.52x10-7 M
4.00x10-6 M
4.00x10-6 M
4.00x10-6 M
2.67x10-6 M
2.67x10-6 M
2.67x10-6 M
1.78x10-6 M
1.78x10-6 M
1.78x10-6 M
1.19x10-6 M
1.19x10-6 M
Absorbance (OD)
Wavelength (nm)
E
Manual Experiment (Duplicate study)
[Rhodamine B in methanol]
6.93 μM Trial 1
6.93 μM Trial 2
4.62 μM Trial 1
4.62 μM Trial 2
3.08 μM Trial 1
3.08 μM Trial 2
2.05 μM Trial 1
2.05 μM Trial 2
1.37 μM Trial 1
1.37 μM Trial 2
Absorbance (OD)
Wavelength (nm)
Cl-
CO2H
F
Automated Experiment (Triplicate Study)
[Rhodamine B in methanol]
2.67x10-6 M
1.78x10-6 M
1.78x10-6 M
1.78x10-6 M
1.19x10-6 M
1.19x10-6 M
1.19x10-6 M
0.79x10-6 M
0.79x10-6 M
0.79x10-6 M
9.00x10-6 M
9.00x10-6 M
9.00x10-6 M
6.00x10-6 M
6.00x10-6 M
6.00x10-6 M
4.00x10-6 M
4.00x10-6 M
4.00x10-6 M
2.67x10-6 M
2.67x10-6 M
Absorbance (OD)
Wavelength (nm)

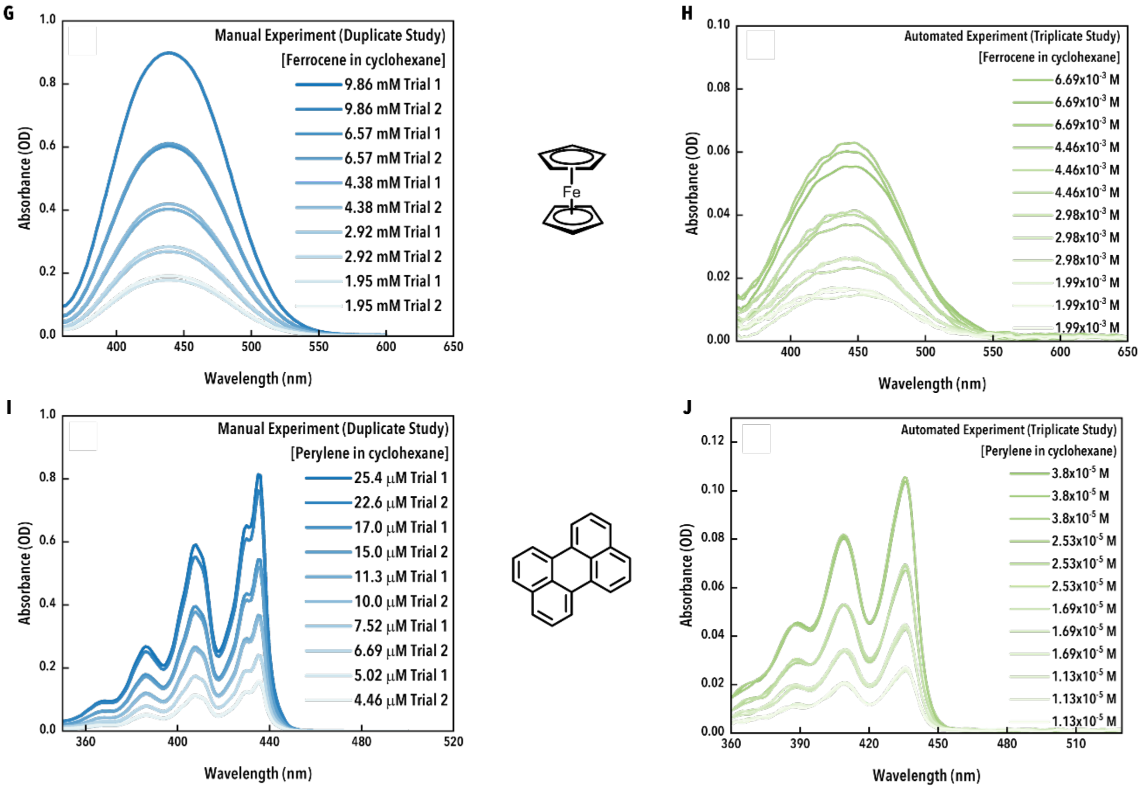

Figure S8. Manual (left, blue) and automated (right, green) absorbance spectra at multiple concentrations. (**A**, **B**) $Ru(bpy)_3(PF_6)_2$ in acetonitrile, (**C**, **D**) zinc(II) phthalocyanine (ZnPc) in pyridine, (**E**, **F**) rhodamine B in methanol, (**G**, **H**) ferrocene in cyclohexane, and (**I**, **J**) perylene in cyclohexane.

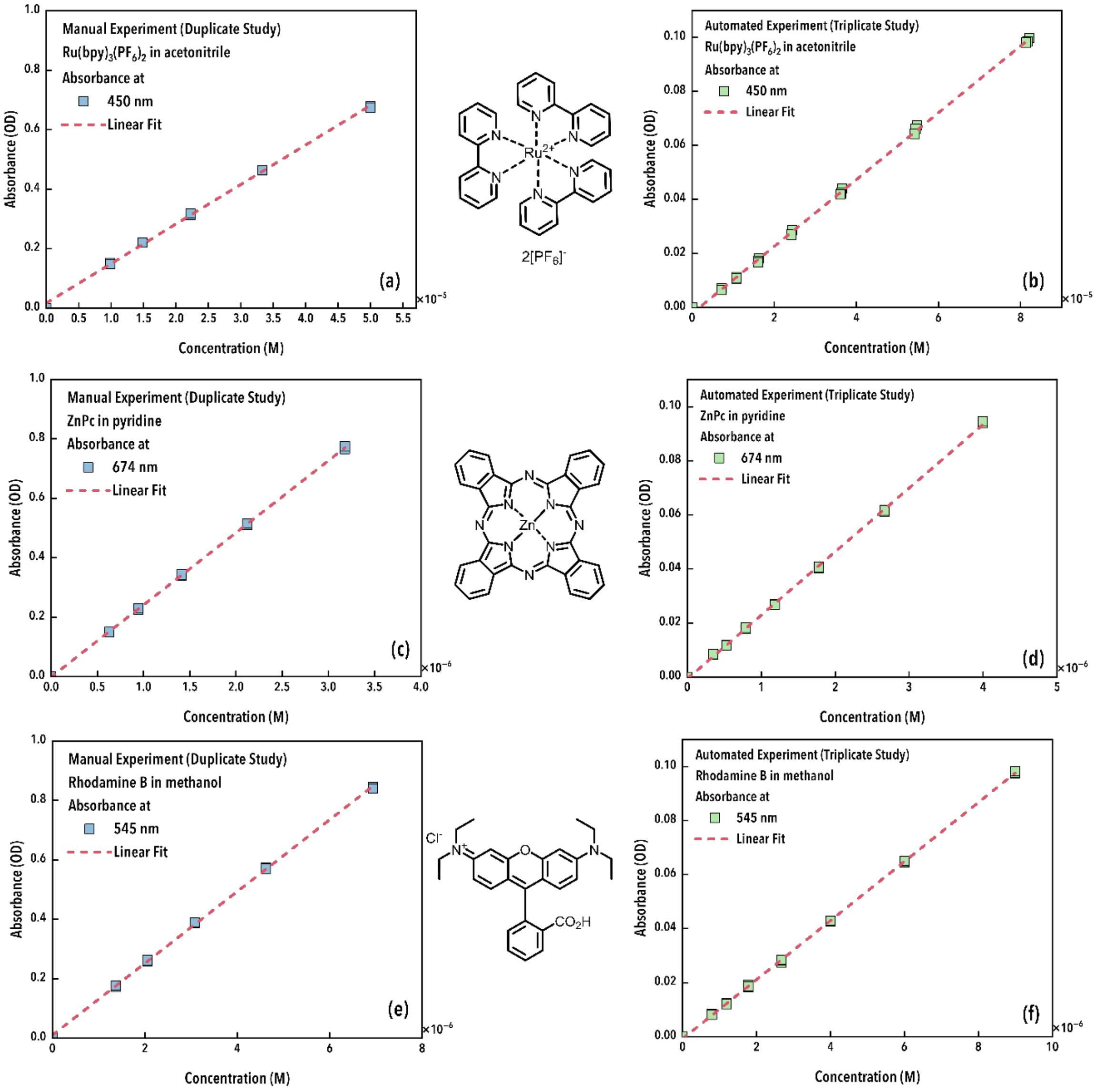
Manual Experiment (Duplicate Study)
Ru(bpy)3(PF6)2 in acetonitrile
Absorbance at
450 nm
Linear Fit
(a)
Automated Experiment (Triplicate Study)
Ru(bpy)3(PF6)2 in acetonitrile
Absorbance at
450 nm
Linear Fit
(b)
2[PF6]-
Manual Experiment (Duplicate Study)
ZnPc in pyridine
Absorbance at
674 nm
Linear Fit
(c)
Automated Experiment (Triplicate Study)
ZnPc in pyridine
Absorbance at
674 nm
Linear Fit
(d)
Manual Experiment (Duplicate Study)
Rhodamine B in methanol
Absorbance at
545 nm
Linear Fit
(e)
Automated Experiment (Triplicate Study)
Rhodamine B in methanol
Absorbance at
545 nm
Linear Fit
(f)
Absorbance (OD)
Concentration (M)

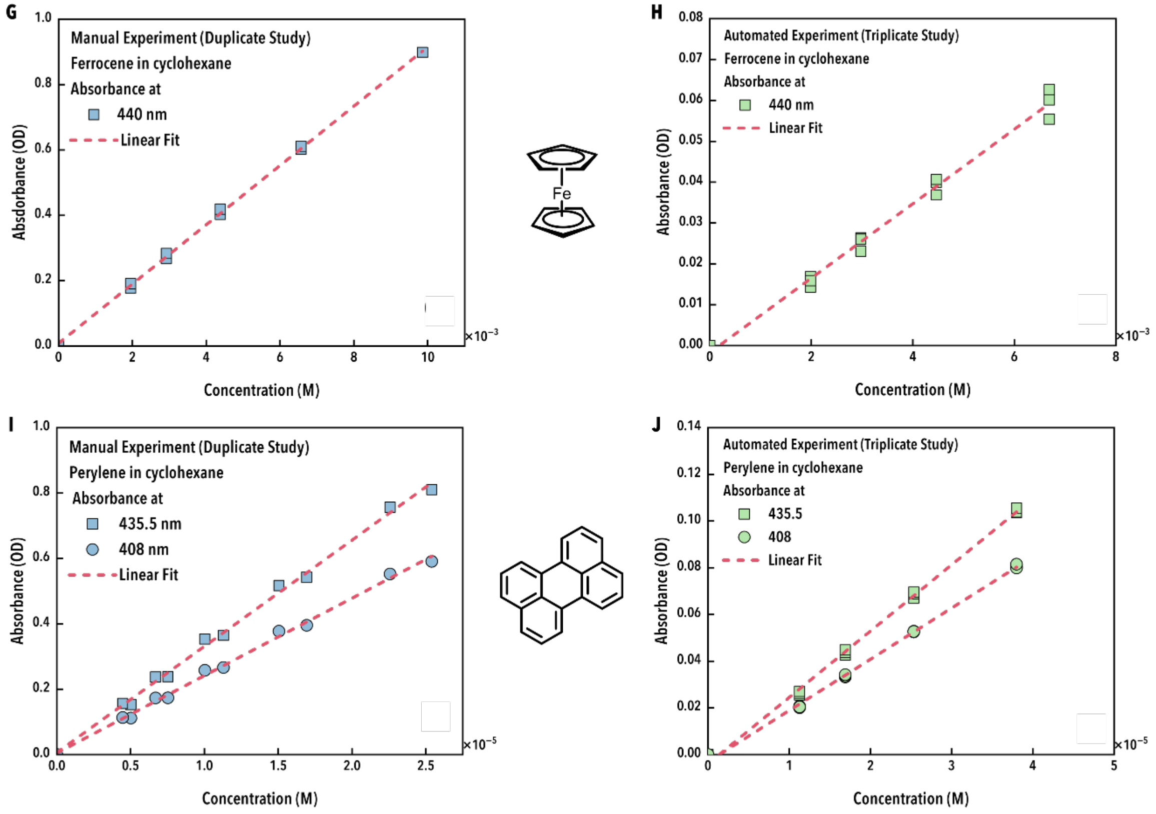

Figure S9. Manual (left, blue) and automated (right, green) absorbance at λ_{max} plotted against concentration. (**A**, **B**) $Ru(bpy)_3(PF_6)_2$ in acetonitrile, (**C**, **D**) zinc(II) phthalocyanine (ZnPc) in pyridine, (**E**, **F**) rhodamine B in methanol, (**G**, **H**) ferrocene in cyclohexane, and (**I**, **J**) perylene in cyclohexane. Linear regression fits are shown for each data set, with slopes used to calculate the molar extinction coefficients.

Table S6. (Manual) Absorbance values at λ_{max}, concentrations, average slopes, coefficients of determination (R^2), and molar extinction coefficients (ε, M^{-1} cm^{-1}) with uncertainties from duplicate manual studies of $Ru(bpy)_3(PF_6)_2$, zinc phthalocyanine (ZnPc), rhodamine B, ferrocene, and perylene in various solvents. Regression is provided a point at the origin (0 absorbance at 0 concentration) instead of fixing the y-intercept of the regression. Literature ε values are included for comparison.

	Trial	Concentration (M)	Absorbance (OD)	Av. Slope (M^{-1})	R^2	ε (M^{-1} cm^{-1})	Lit. ε (M^{-1} cm^{-1})
$Ru(bpy)_3(PF_6)_2$ in acetonitrile	1	5.00×10^{-5}	0.678	13,300 (450nm)	0.999	13,300 ±145	13,000 (452nm) Ref.[7]
		3.33×10^{-5}	0.463				
		2.22×10^{-5}	0.313				
		1.48×10^{-5}	0.220				
		9.88×10^{-6}	0.152				
	2	5.00×10^{-5}	0.675				
		3.33×10^{-5}	0.464				
		2.22×10^{-5}	0.319				
		1.48×10^{-5}	0.220				
		9.88×10^{-6}	0.148				
ZnPc in pyridine	1	3.18×10^{-6}	0.766	243,000 (674nm)	1.000	243,000 ±984	277,000 (674nm) Ref.[7]
		2.12×10^{-6}	0.510				
		1.41×10^{-6}	0.341				
		9.42×10^{-7}	0.226				
		6.28×10^{-7}	0.147				
	2	3.18×10^{-6}	0.775				
		2.12×10^{-6}	0.516				
		1.41×10^{-6}	0.344				
		9.42×10^{-7}	0.229				
		6.28×10^{-7}	0.150				

(**Table S6** continued; Manual)

	Trial	Concentration	Absorbance	Av. Slope	R^2	ε	Lit. ε
Rhodamine B in methanol	1	6.93×10^{-6}	0.845	121,000 (545nm)	1.000	121,000 ±795	107,500 (546nm) Ref.[7]
		4.62×10^{-6}	0.574				
		3.08×10^{-6}	0.387				
		2.05×10^{-6}	0.259				
		1.37×10^{-6}	0.173				
	2	6.93×10^{-6}	0.840				
		4.62×10^{-6}	0.570				
		3.08×10^{-6}	0.389				
		2.05×10^{-6}	0.262				
		1.37×10^{-6}	0.177				
Ferrocene in cyclohexane	1	9.86×10^{-3}	0.898	91 (440nm)	1.000	91 ±0.78	96 (442nm) Ref.[7]
		6.57×10^{-3}	0.611				
		4.38×10^{-3}	0.419				
		2.92×10^{-3}	0.283				
		1.95×10^{-3}	0.191				
	2	9.86×10^{-3}	0.898				
		6.57×10^{-3}	0.611				
		4.38×10^{-3}	0.419				
		2.92×10^{-3}	0.283				
		1.95×10^{-3}	0.191				
Perylene in cyclohexane[a]	1	2.54×10^{-5}	0.811	32,500 (435.5nm)	0.998	32,500 ±673	32,000 (435.5nm) Ref.[7]
		1.69×10^{-5}	0.543				
		1.13×10^{-5}	0.365				
		7.53×10^{-6}	0.238				
		5.02×10^{-6}	0.153				
	2	2.26×10^{-5}	0.757				
		1.51×10^{-5}	0.517				
		1.00×10^{-5}	0.353				
		6.69×10^{-6}	0.237				
		4.46×10^{-6}	0.156				
Perylene in cyclohexane[a]	1	2.54×10^{-5}	0.591	23,700 (408nm)	0.998	23,700 ±500	23,500 (408nm) Ref.[7]
		1.69×10^{-5}	0.395				
		1.13×10^{-5}	0.266				
		7.53×10^{-6}	0.174				
		5.02×10^{-6}	0.111				
	2	2.26×10^{-5}	0.552				
		1.51×10^{-5}	0.377				
		1.00×10^{-5}	0.258				
		6.69×10^{-6}	0.173				
		4.46×10^{-6}	0.113				

[a] Both perylene in cyclohexane entries (per trial) are from the same experiment.

Table S7. (Automated) Absorbance values at λ_{max}, concentrations, average slopes, coefficients of determination (R^2), and molar extinction coefficients (ε, M^{-1} cm^{-1}) with uncertainties from triplicated automated studies of $Ru(bpy)_3(PF_6)_2$, zinc phthalocyanine (ZnPc), rhodamine B, ferrocene, and perylene in various solvents. Regression is provided a point at the origin (0 absorbance at 0 concentration) instead of fixing the y-intercept of the regression. Literature ε values are included for comparison

	Trial	Concentration (M)	Absorbance (OD)	Av. Slope (M^{-1})	R^2	ε (M^{-1} cm^{-1})	Lit. ε (M^{-1} cm^{-1})
$Ru(bpy)_3(PF_6)_2$ in acetonitrile	1	8.21×10^{-5}	0.100	1,238 (450 nm)	1.000	12,400 ±500	13,000 Ref.[7]
		5.48×10^{-5}	0.067				
		3.65×10^{-5}	0.044				
		2.44×10^{-5}	0.029				
		1.63×10^{-5}	0.018				
		1.08×10^{-5}	0.011				
		7.23×10^{-6}	0.007				
	2	8.16×10^{-5}	0.098				
		5.45×10^{-5}	0.066				
		3.63×10^{-5}	0.042				
		2.42×10^{-5}	0.027				
		1.62×10^{-5}	0.017				
		1.08×10^{-5}	0.011				
		7.19×10^{-5}	0.007				
	3	8.13×10^{-5}	0.098				
		5.42×10^{-5}	0.064				
		3.62×10^{-5}	0.042				
		2.41×10^{-5}	0.027				
		1.61×10^{-5}	0.017				
		1.07×10^{-5}	0.011				
		7.16×10^{-6}	0.006				

(**Table S7** continued; Automated)

	Trial	Concentration (M)	Absorbance (OD)	Av. Slope (M^{-1})	R^2	*ε* (M^{-1} cm^{-1})	Lit. *ε* (M^{-1} cm^{-1})
ZnPc in pyridine	1	4.00×10^{-6}	0.094	23,517 (674 nm)	1.000	235,000 ±9460	277,000 Ref.[7]
		2.67×10^{-6}	0.061				
		1.78×10^{-6}	0.040				
		1.19×10^{-6}	0.0268				
		7.92×10^{-7}	0.018				
		5.28×10^{-7}	0.012				
		3.52×10^{-7}	0.008				
	2	4.00×10^{-6}	0.094				
		2.67×10^{-6}	0.062				
		1.78×10^{-6}	0.041				
		1.19×10^{-6}	0.027				
		7.92×10^{-7}	0.018				
		5.28×10^{-7}	0.012				
		3.52×10^{-7}	0.008				
	3	4.00×10^{-6}	0.094				
		2.67×10^{-6}	0.062				
		1.78×10^{-6}	0.041				
		1.19×10^{-6}	0.027				
		7.92×10^{-7}	0.018				
		5.28×10^{-7}	0.012				
		3.52×10^{-7}	0.008				

(**Table S7** continued; Automated)

	Trial	Concentration (M)	Absorbance (OD)	Av. Slope (M^{-1})	R^2	ε (M^{-1} cm^{-1})	Lit. ε (M^{-1} cm^{-1})
Rhodamine B in methanol	1	9.00×10^{-6}	0.098	10,929 (545 nm)	1.000	109,000 ±4380	107,500 Ref.[7]
		6.00×10^{-6}	0.065				
		4.00×10^{-6}	0.043				
		2.67×10^{-6}	0.027				
		1.78×10^{-6}	0.018				
		1.19×10^{-6}	0.012				
		7.93×10^{-7}	0.008				
	2	9.00×10^{-6}	0.098				
		6.00×10^{-6}	0.065				
		4.00×10^{-6}	0.043				
		2.67×10^{-6}	0.028				
		1.78×10^{-6}	0.019				
		1.19×10^{-6}	0.012				
		7.93×10^{-7}	0.008				
	3	9.00×10^{-6}	0.098				
		6.00×10^{-6}	0.065				
		4.00×10^{-6}	0.043				
		2.67×10^{-6}	0.028				
		1.78×10^{-6}	0.019				
		1.19×10^{-6}	0.012				
		7.93×10^{-7}	0.008				

(**Table S7** continued; Automated)

	Trial	Concentration (M)	Absorbance (OD)	Av. Slope (M^{-1})	R^2	ε (M^{-1} cm^{-1})	Lit. ε (M^{-1} cm^{-1})
Ferrocene in cyclohexane	1	6.69×10^{-3}	0.055	9.1 (440 nm)	0.994	91 ±5	96 Ref.[7]
		4.46×10^{-3}	0.037				
		2.98×10^{-3}	0.023				
		1.99×10^{-3}	0.014				
	2	6.69×10^{-3}	0.060				
		4.46×10^{-3}	0.040				
		2.98×10^{-3}	0.026				
		1.99×10^{-3}	0.017				
	3	6.69×10^{-3}	0.063				
		4.46×10^{-3}	0.041				
		2.98×10^{-3}	0.026				
		1.99×10^{-3}	0.016				
Perylene in cyclohexane [a]	1	3.80×10^{-5}	0.104	2,840 (435.5 nm)	0.999	28,400 ±1220	32,000 Ref.[7]
		2.53×10^{-5}	0.067				
		1.69×10^{-5}	0.043				
		1.13×10^{-5}	0.026				
	2	3.80×10^{-5}	0.104				
		2.53×10^{-5}	0.069				
		1.69×10^{-5}	0.044				
		1.13×10^{-5}	0.026				
	3	3.80×10^{-5}	0.106				
		2.53×10^{-5}	0.070				
		1.69×10^{-5}	0.045				
		1.13×10^{-5}	0.027				
Perylene in cyclohexane [a]	1	3.80×10^{-5}	0.081	2,193 (408 nm)	0.999	21,900 ±928	23,500 Ref.[7]
		2.53×10^{-5}	0.053				
		1.69×10^{-5}	0.033				
		1.13×10^{-5}	0.021				
	2	3.80×10^{-5}	0.080				
		2.53×10^{-5}	0.053				
		1.69×10^{-5}	0.034				
		1.13×10^{-5}	0.020				
	3	3.80×10^{-5}	0.082				
		2.53×10^{-5}	0.053				
		1.69×10^{-5}	0.034				
		1.13×10^{-5}	0.020				

[a] Both perylene in cyclohexane entries are from the same experiment.

5.5. In-vial *vs* in-needle

While it is possible to perform the Beer–Lambert extinction assay's serial dilution in the liquid handler's tubing (reducing material consumption about 5-fold and time per data point about 2-fold), the protocol comes with a maintenance challenge.

Chronologically before the full Beer–Lambert campaign, a test was performed comparing $Ru(bpy)_3(PF_6)_2$ in acetonitrile (the same as the system fluid) and Rhodamine B in methanol using an in-vial and in-needle approach. The in-needle procedure is almost the same as the in-vial procedure: The in-vial approach discards a third of the needle's contents, replaces the volume with diluent, then mixes within the needle using aspiration–dispense cycles. Using the average of three replicates (all $R^2 > 0.998$), the measured extinction coefficients of $Ru(bpy)_3(PF_6)_2$ and Rhodamine B differ by 2.7 and 7.9%, respectively, between their in-vial and in-needle methods. When repeating the in-needle assay a month later, the droplets could be observed breaking apart in the system line and the quality of the linear regressions was reduced.

The authors hypothesize that gradual degradation of the tubing with prolonged exposure to acetonitrile can change the wettability of the tubing and cause droplets of sample and system fluid to be left behind (resulting in accelerated dilution). If the Beer–Lambert extinction coefficient study were performed in this way, the tubing through the flow cell would need to be periodically replaced or as droplet residue formation becomes observable. This renders this segment of tubing a consumable item and necessitates the continual re-administration of an effective-pathlength study (as the inner diameter of the replacement tubing is variable). Note that this change in wettability is less of an issue for the Stern–Volmer and relative PLQY experiments. In the former, each droplet is prepared separately and so this error does not compound and is consistent between all samples. In the latter, the same droplet is used for the absorbance and PL measurements, self-correcting for any aberrations in the absolute concentration.

6. Relative photoluminescence quantum yield study

6.1. Materials and chemicals

9,10-Diphenylanthracene (DPA) and perylene were purchased from Alfa Aesar. Anthracene was obtained from Sigma-Aldrich. 2,2'-(1,4-Phenylene)bis(5-phenyl-1,3-oxazole) (POPOP) was purchased from J.T. Baker, and platinum(II) octaethylporphyrin (PtOEP) was obtained from Frontier Specialty Chemicals. Tris(2,2′-bipyridine)ruthenium(II) hexafluorophosphate ($Ru(bpy)_3(PF_6)_2$) was synthesized in-house according to a previously reported literature procedure[6]. Spectroscopic grade solvents were purchased as follows: toluene from J.T. Baker, acetonitrile from Macron Fine Chemicals, and cyclohexane from Thermo Fisher Scientific. All commercially obtained chemicals were used as received.

6.2. Manual photophysical methods

All samples and reference solutions were prepared in a standard 1 cm^2 quartz cuvette and equilibrated with air prior to measurement. Absorption and PL spectra were recorded at room temperature and atmospheric pressure. Serial dilutions were prepared to obtain multiple concentrations with absorbance values ranging from 0.001 to 0.08 OD at the excitation wavelength (365 nm) to minimize inner filter effects and ensure linearity of the PL response. Absorption spectra were recorded using a Cary 60 Bio UV-vis spectrophotometer (Agilent Technologies) with baseline and solvent blank corrections applied. PL spectra were collected on a Cary Eclipse Fluorimeter (Agilent Technologies), with solvent blank subtraction used to remove any background signals. All spectral measurements for samples and reference were conducted under identical instrument settings and conditions.

6.3. Automated photophysical methods

The automated procedure for the relative PLQY measurements was identical to that of the automated Beer–Lambert measurements with the exception of a PL measurement. The PL measurement is performed on the same droplet as the absorbance measurement (after the absorbance measurement) and uses the average of 5 scans integrating over 2 seconds each, spaced 0.1 seconds apart. Excitation is at 365 nm using an LED (ThorLabs).

6.4. Calculation of photoluminescence quantum yields

PLQYs were determined using the relative method, employing DPA in cyclohexane ($\Phi = 0.93$[8]) as the reference standard. The integrated PL intensity of the emission bands for each sample was plotted against the corresponding absorption correction factor, $(1 - 10^{-A})$, where A is the absorbance at the excitation wavelength. Linear regression was applied to each dataset, and the slope was used to calculate the PLQY according to the equation:

$$\Phi_{sample} = \Phi_{reference} \times \left(\frac{slope_{sample}}{slope_{reference}}\right) \times \left(\frac{n_{D,sample}^2}{n_{D,reference}^2}\right) \tag{13}$$

where Φ_{sample} and $\Phi_{reference}$ are the quantum yields of the sample and reference, respectively; $slope_{sample}$ and $slope_{reference}$ are the slopes of the integrated PL intensity versus $(1 - 10^{-A})$ plots; and $n_{D,sample}$ and $n_{D,reference}$ are the refractive indices of the solvents used (cyclohexane for reference—DPA—, and the corresponding solvent for the sample).

Uncertainty in the PLQY ($\Delta\Phi_{sample}$) was calculated by propagating the uncertainties from the linear regression slopes and the reported uncertainty in the reference quantum yield. The

uncertainty in the refractive indices was assumed to be negligible. The combined uncertainty was calculated using the following formula:

$$\Delta\Phi_{sample} = \Phi_{sample} \times \sqrt{\left(\frac{\Delta\Phi_{reference}}{\Phi_{reference}}\right)^2 + \left(\frac{\Delta slope_{sample}}{slope_{sample}}\right)^2 + \left(\frac{\Delta slope_{reference}}{slope_{reference}}\right)^2} \quad (14)$$

where $\Delta\Phi_{reference}$ is the uncertainty in the literature value of the reference quantum yield, and $\Delta slope_{sample}$ and $\Delta slope_{reference}$ are the standard errors of the slopes obtained from the linear regression.

All data analysis, spectral fitting, and graphical representation were performed using OriginPro 2023b. The results for the five fluorophores studied using both manual experimentation (conducted in duplicate) and the automated workflow (conducted in triplicate) are summarized in **Figure S10** and **Figure S11** and in **Table S8**–**Table S11**.

For the automated study, samples exhibiting an absorbance below 0.002 OD at 365 nm were excluded from PL analysis, as their emission spectra lacked sufficient signal-to-noise ratio, resulting in a loss of spectral integrity. The only exception to this threshold was POPOP, which maintained well-resolved spectral features down to an optical density (OD) of 0.001 due to its highly emissive nature. Conversely, for strongly luminescent fluorophores such as DPA, perylene, and POPOP, PL spectra that saturated the detector (*i.e.*, emission intensities exceeding 200,000 PL units) were also discarded. These spectra were excluded from PLQY calculations to ensure measurements remained within the detector's linear dynamic range.

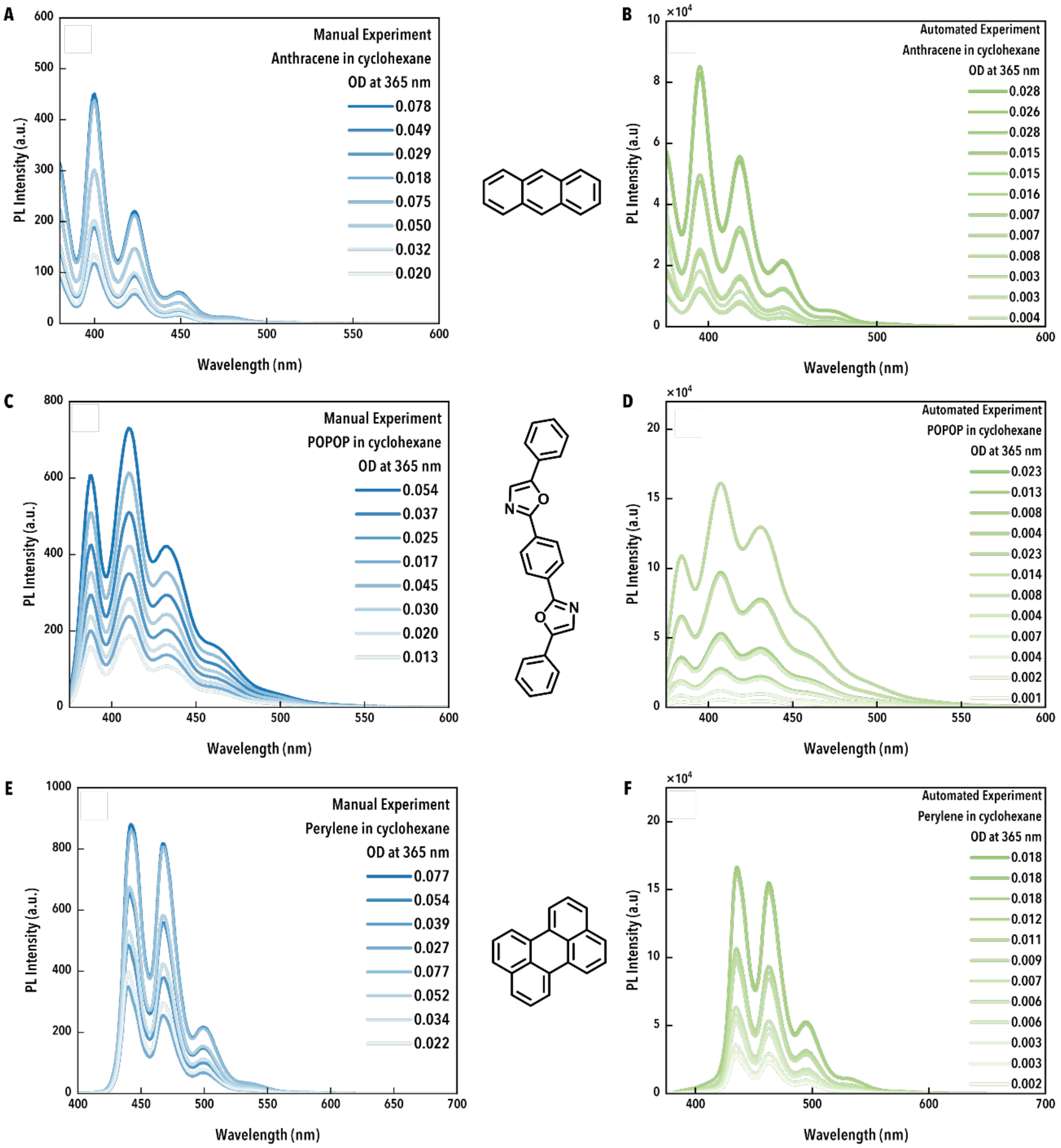
A
Manual Experiment
Anthracene in cyclohexane
OD at 365 nm
0.078
0.049
0.029
0.018
0.075
0.050
0.032
0.020
PL Intensity (a.u.)
Wavelength (nm)
B
×10^4
Automated Experiment
Anthracene in cyclohexane
OD at 365 nm
0.028
0.026
0.028
0.015
0.015
0.016
0.007
0.007
0.008
0.003
0.003
0.004
PL Intensity (a.u)
Wavelength (nm)
C
Manual Experiment
POPOP in cyclohexane
OD at 365 nm
0.054
0.037
0.025
0.017
0.045
0.030
0.020
0.013
PL Intensity (a.u.)
Wavelength (nm)
N
O
D
×10^4
Automated Experiment
POPOP in cyclohexane
OD at 365 nm
0.023
0.013
0.008
0.004
0.023
0.014
0.008
0.004
0.007
0.004
0.002
0.001
PL Intensity (a.u.)
Wavelength (nm)
E
Manual Experiment
Perylene in cyclohexane
OD at 365 nm
0.077
0.054
0.039
0.027
0.077
0.052
0.034
0.022
PL Intensity (a.u.)
Wavelength (nm)
F
×10^4
Automated Experiment
Perylene in cyclohexane
OD at 365 nm
0.018
0.018
0.018
0.012
0.011
0.009
0.007
0.006
0.006
0.003
0.003
0.002
PL Intensity (a.u)
Wavelength (nm)

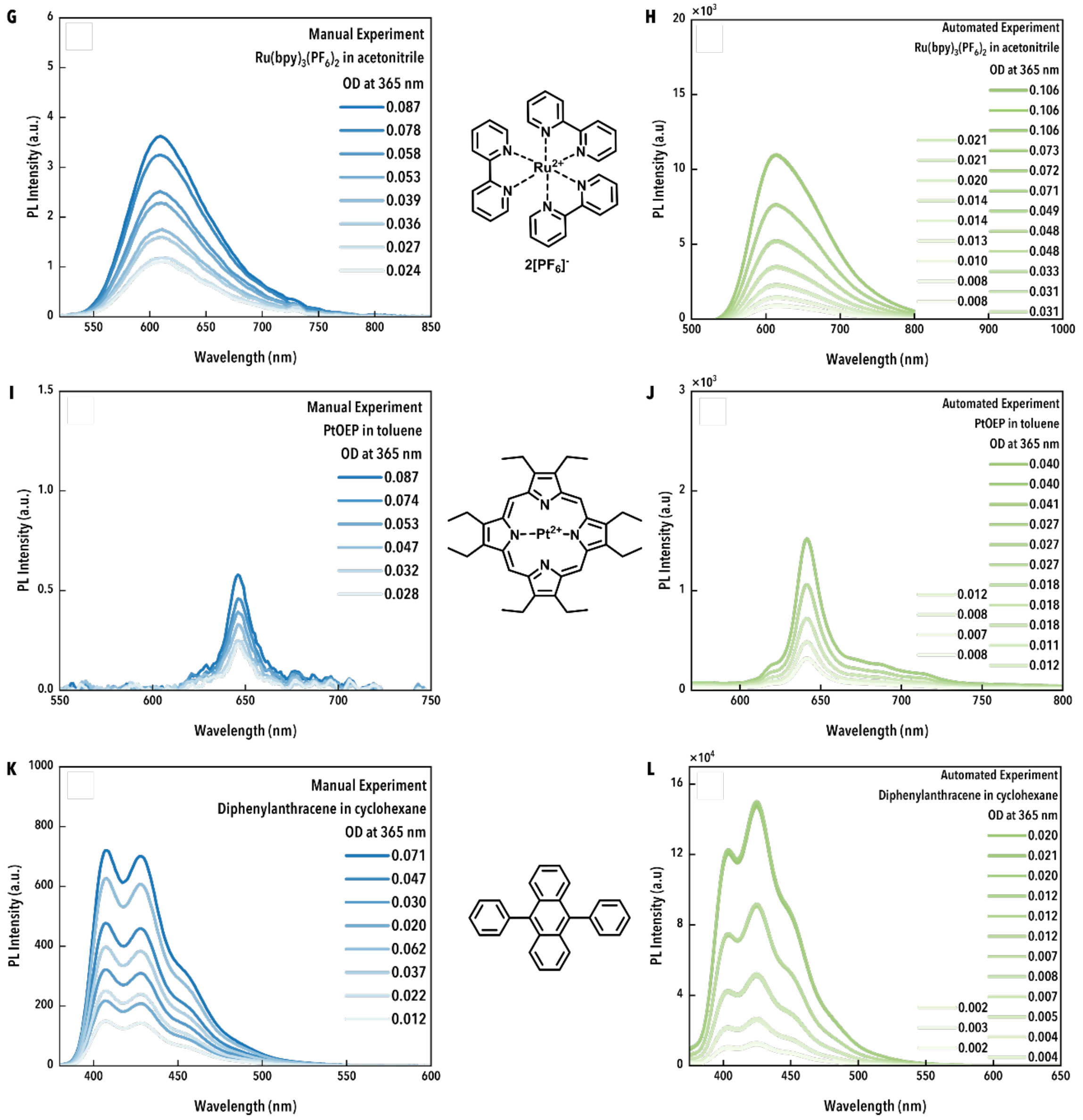

Figure S10. Manual (left, blue) and automated (right, green) room-temperature photoluminescence (PL) spectra as a function of wavelength for serial dilutions of six fluorophores, measured in air-equilibrated solutions as part of the photoluminescence quantum yield (PLQY) study. All samples were photoexcited at 365 nm. (**A**, **B**) Anthracene in cyclohexane, (**C**, **D**) 2,2'-(1,4-Phenylene)bis(5-phenyl-1,3-oxazole) (POPOP) in cyclohexane, (**E**, **F**) Perylene in cyclohexane, (**G**, **H**) Ru(bpy)$_3$(PF$_6$)$_2$ in acetonitrile, (**I**, **J**) Platinum(II) octaethylporphyrin (PtOEP) in toluene, and (**K**, **L**) 9,10-diphenylanthracene (DPA) in cyclohexane. DPA was used as the PL reference standard for PLQY determination.

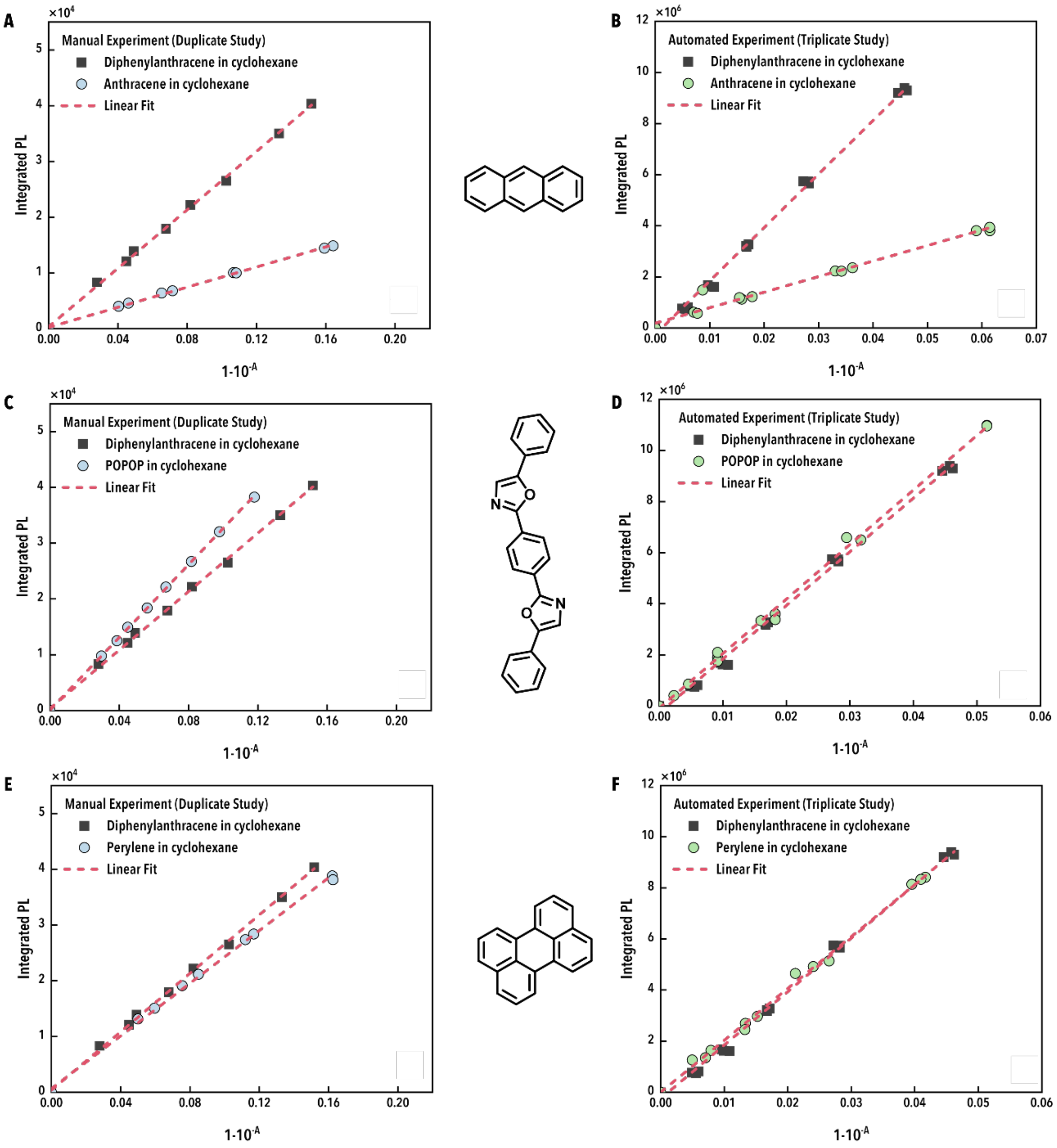
A
×10^4
Manual Experiment (Duplicate Study)
Diphenylanthracene in cyclohexane
Anthracene in cyclohexane
Linear Fit
Integrated PL
1-10^-A
B
×10^6
Automated Experiment (Triplicate Study)
Diphenylanthracene in cyclohexane
Anthracene in cyclohexane
Linear Fit
Integrated PL
1-10^-A
C
×10^4
Manual Experiment (Duplicate Study)
Diphenylanthracene in cyclohexane
POPOP in cyclohexane
Linear Fit
Integrated PL
1-10^-A
N
O
D
×10^6
Automated Experiment (Triplicate Study)
Diphenylanthracene in cyclohexane
POPOP in cyclohexane
Linear Fit
Integrated PL
1-10^-A
E
×10^4
Manual Experiment (Duplicate Study)
Diphenylanthracene in cyclohexane
Perylene in cyclohexane
Linear Fit
Integrated PL
1-10^-A
F
×10^6
Automated Experiment (Triplicate Study)
Diphenylanthracene in cyclohexane
Perylene in cyclohexane
Linear Fit
Integrated PL
1-10^-A

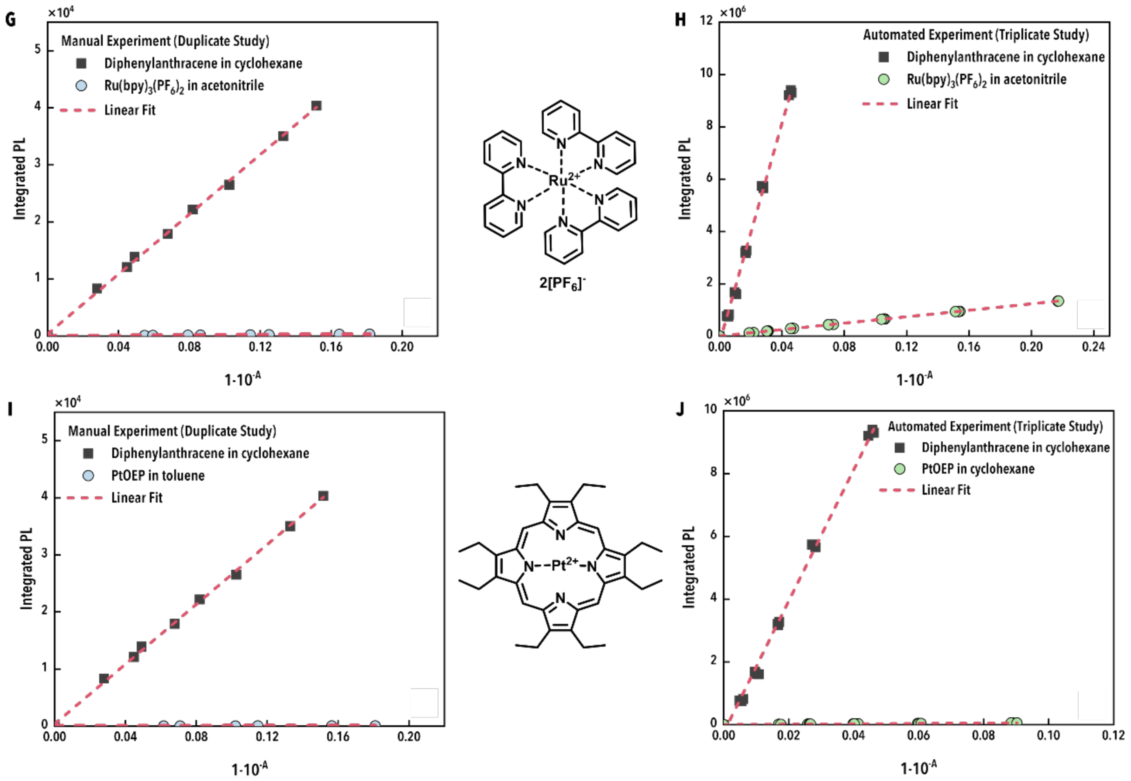

Figure S11. Manual (left, blue) and automated (right, green) linear fits of integrated photoluminescence (PL) intensity vs. absorption correction factor ($1 - 10^{-A}$) for serial dilutions of fluorophores, measured in air-equilibrated solutions as part of the photoluminescence quantum yield (PLQY) study. DPA in cyclohexane was used as the PL reference standard in all cases. (**A**, **B**) Anthracene in cyclohexane, (**C**, **D**) 2,2'-(1,4-Phenylene)bis(5-phenyl-1,3-oxazole) (POPOP) in cyclohexane, (**E**, **F**) Perylene in cyclohexane, (**G**, **H**) $Ru(bpy)_3(PF_6)_2$ in acetonitrile, and (**I**, **J**) Platinum(II) octaethylporphyrin (PtOEP) in toluene. All samples were photoexcited at 365 nm. Slopes from linear regression were used for relative PLQY determination.

Table S8. (Manual) Absorption correction factors ($1 - 10^{-A}$), integrated photoluminescence (PL) intensities, slopes, and coefficients of determination (R^2) from linear fits of serial dilutions measured in air-equilibrated solutions during duplicate trials of the manual photoluminescence quantum yield (PLQY) study. Regression is provided a point at the origin instead of fixing the y-intercept of the regression. 9,10-Diphenylanthracene (DPA) in cyclohexane was the PL reference standard.

Fluorophore	Trial	$1–10^{-A}$	Integrated PL	Slope	R^2
DPA in cyclohexane (Reference)	1	0.152	40365	261,925	0.999
		0.102	26505		
		0.068	17919		
		0.045	12094		
	2	0.133	35004		
		0.082	22181		
		0.049	13917		
		0.028	8324		
Anthracene in cyclohexane	1	0.164	14867	90,153	0.999
		0.107	10063		
		0.065	6394		
		0.040	4012		
	2	0.159	14430		
		0.108	10011		
		0.071	6802		
		0.046	4579		
POPOP in cyclohexane	1	0.118	38265	326,037	1.000
		0.082	26706		
		0.056	18384		
		0.039	12497		
	2	0.098	32058		
		0.067	22134		
		0.045	14930		
		0.030	9778		

(**Table S8** continued; Manual)

Fluorophore	Trial	1–10^{-A}	Integrated PL	Slope	R^2
Perylene in cyclohexane	1	0.162	38808	236,190	0.999
		0.112	27362		
		0.075	19115		
		0.050	13145		
	2	0.163	38116		
		0.117	28380		
		0.085	21162		
		0.060	15062		
Rubpy in acetonitrile	1	0.165	299	1,816	1.000
		0.114	210		
		0.079	146		
		0.055	101		
	2	0.182	329		
		0.125	231		
		0.086	162		
		0.059	110		
PtOEP in toluene	1	0.156	11	75	0.995
		0.102	8		
		0.062	5		
	2	0.181	14		
		0.115	10		
		0.071	6		

Table S9. (Manual) Manual photoluminescence quantum yields (PLQYs) for various fluorophores measured in air-equilibrated solutions at 365 nm excitation, along with the corresponding solvent refractive indices (n_D). PLQYs were calculated using **Eq. 13**, and their associated uncertainties were determined by error propagation using **Eq. 14**. Literature values are provided for comparison. 9,10-Diphenylanthracene (DPA) in cyclohexane was used as the photoluminescence reference standard.

Fluorophore	Solvent	n_D [a]	PLQY (Manual)	PLQY (Literature)	
DPA	Cyclohexane	1.426	Reference	0.93 ±0.03	Ref.[9]
Anthracene			0.30 ±0.01	0.28 ±0.02	Ref.[10]
POPOP			1.00 ±0.04	0.98 ±0.03	Ref.[8]
Perylene			0.84 ±0.03	0.94 ±0.05	Ref.[11,12]
$Ru(bpy)_3(PF_6)_2$	Acetonitrile	1.344	0.0100 ±0.0002	0.018 ±0.002	Ref.[10]
PtOEP	Toluene	1.497	0.00030 ±0.00002	<0.001	Ref.[13]

[a] Refractive indices provided by Ref.[7]

Table S10. (Automated) Absorption correction factors ($1 - 10^{-A}$), integrated photoluminescence (PL) intensities, slopes, and coefficients of determination (R^2) from linear fits of serial dilutions measured in air-equilibrated solutions during triplicate trials of the automated photoluminescence quantum yield (PLQY) study. Regression provides a point at the origin instead of fixing the y-intercept of the regression. 9,10-Diphenylanthracene (DPA) in cyclohexane served as the PL reference standard.

Fluorophore	Trial	$1–10^{-A}$	Integrated PL	Slope	R^2
DPA in cyclohexane (Reference)	1	0.045	9205284	2.10×10^8	0.998
		0.028	5656264		
		0.017	3174826		
		0.011	1606358		
		0.006	749776		
	2	0.046	9301932		
		0.028	5736352		
		0.017	3270133		
		0.01	1681604		
		0.006	817872		
	3	0.046	9399242		
		0.027	5739375		
		0.017	3209990		
		0.01	1620429		
		0.005	762955		

(**Table S10** continued; Automated)

Fluorophore	Trial	$1–10^{–A}$	Integrated PL	Slope	R^2
Anthracene in cyclohexane	1	0.061	3808150	6.08×10^7	0.986
		0.033	2231212		
		0.016	1132413		
		0.007	616736		
	2	0.059	3812095		
		0.034	2224225		
		0.015	1189889		
		0.008	575781		
	3	0.061	3938744		
		0.036	2356042		
		0.018	1227042		
		0.009	1486224		
POPOP in cyclohexane	1	0.052	1.1E+07	2.13×10^8	0.999
		0.031	6590634		
		0.017	3604181		
		0.01	1896243		
	2	0.015	3338622		
		0.01	1756367		
		0.004	857054		
		0.003	407332		
	3	0.051	1.1E+07		
		0.031	6501270		
		0.018	3379190		
		0.009	2095077		

(**Table S10** continued; Automated)

Fluorophore	Trial	$1–10^{–A}$	Integrated PL	Slope	R^2
Perylene in cyclohexane	1	0.042	8414168	2.03×10^8	0.999
		0.027	5139367		
		0.015	2967487		
		0.008	1639489		
	2	0.041	8331604		
		0.024	4916904		
		0.013	2698216		
		0.007	1348436		
	3	0.04	8137134		
		0.021	4649747		
		0.013	2449594		
		0.005	1262245		
$Ru(bpy)_3(PF_6)_2$ in acetonitrile	1	0.217	1335736	6.16×10^6	1
		0.154	943400		
		0.106	653637		
		0.073	444614		
		0.047	298785		
		0.031	198231		
		0.022	130913		
	2	0.217	1344205		
		0.153	942462		
		0.105	648119		
		0.07	437046		
		0.048	293223		
		0.031	192265		
		0.019	122948		
	3	0.217	1339872		
		0.151	937092		
		0.104	643119		
		0.07	433417		
		0.046	287537		
		0.03	187918		
		0.019	121610		

(**Table S10** continued; Automated)

Fluorophore	Trial	$1{-}10^{-A}$	Integrated PL	Slope	R^2
PtOEP in toluene	1	0.089	54495	6.08 ×10^5	0.998
		0.06	39016		
		0.04	27268		
		0.026	18609		
		0.018	12577		
	2	0.088	54756		
		0.06	39116		
		0.041	27284		
		0.026	18796		
		0.017	12800		
	3	0.09	54643		
		0.061	39084		
		0.04	27163		
		0.027	18575		
		0.018	12513		

Table S11. (Automated) Automated photoluminescence quantum yields (PLQYs) for various fluorophores measured in air-equilibrated solutions at 365 nm excitation, along with the corresponding solvent refractive indices (n_D). PLQYs were calculated using **Eq. 12**, and their associated uncertainties were determined by error propagation using **Eq. 13**. Literature values are provided for comparison. 9,10-Diphenylanthracene (DPA) in cyclohexane was used as the photoluminescence reference standard.

Fluorophore	Solvent	n_D [a]	PLQY (Automated)	PLQY (Literature)	
DPA	Cyclohexane	1.426	Reference	0.93 ±0.03	Ref.[9]
Anthracene			0.27 ±0.02	0.28 ±0.02	Ref.[10]
POPOP			0.95 ±0.04	0.98 ±0.03	Ref.[8]
Perylene			0.90 ±0.03	0.94 0.05	Ref.[11,12]
$Ru(bpy)_3(PF_6)_2$	Acetonitrile	1.344	0.020 ±0.001	0.018 ±0.002	Ref.[10]
PtOEP	Toluene	1.497	0.0030 ±0.0001	<0.001	Ref.[13]

[a] Refractive indices provided by Ref.[7]

7. Comments on automated data processing

For these studies, final data processing was performed manually using Origin 2023b software. During Roblonski platform operation (e.g., to determine the appropriate dilution of stock solutions in Beer–Lambert and relative PLQY studies), data processing was performed using Python scripts. In general, it is challenging to construct a fully generalizable automated spectral data processing protocol that satisfies the level of scrutiny required by the spectroscopy field. To avoid constraining users of this toolkit to a single data-processing workflow, advanced automated data processing was not included in this work.

7.1. Estimations of experimental uncertainty in the Stern–Volmer studies

In the reported automated Stern–Volmer study, the data were generated by performing replicates. In each replicate, the platform could automatically repeat experiments deemed surprising to improve experimental reliability. This results in replicates with unequal quantities of data. Given that Stern–Volmer quenching constants are invariant to minor perturbations of catalyst concentration and dependent solely on the slope of the data (provided the estimated y-intercept is 1) and given how systematic uncertainty is introduced in the preparation of stock solutions, and aleatoric uncertainties are introduced with pipetting, mixing, and spectroscopic measurement, the question of how to process the data to arrive at a quenching constant that is accurate and whose estimated uncertainty accurately reflects these uncertainties is unclear. For the data reported, we chose to process each replicate's data independently (each replicate uses its own no-quencher intensity; when multiple no-quencher intensities are measured within a replicate, the average between the repeated measurements is used for all data in that replicate), then fit a line to all data—across replicates and repeated data—simultaneously.

As seen in **Figure S7**, while replicate studies align almost exactly for 4-nitrobenzaldehyde and pyrene, some exhibit vertical shifts in the data—identical quenching constant—(ferrocene, benzoylferrocene, acridine) and others have a distribution of quenching constants (decamethylferrocene, 1,1'-dimethylferrocene, acetylferrocene, 3-nitrobenzaldehyde, methyl 4-nitrobenzoate, anthracene). The distillation of these replicates into a single quenching constant could be achieved rationally using multiple procedures. Below, we discuss the consequences of these approaches.

Pre-averaging approaches. At each quencher concentration (abscissa), the intensities (I) or inverse intensities (I_0/I) can be averaged to calculate the ordinate. Raw averages obliviate the uncertainties introduced by stock solution preparation and the spectrometer; however, they ensure that the data is equally distributed across the quencher concentration, which is optimal for an unbiased linear estimator. It is possible to restore some knowledge of the uncertainties by using the variance of the averaged data points to inform regression weighting (such as inverse estimated sample variance weighting); however, this approach does not generalize to the case of a single replicate with repeated measurements.

Aggregative approaches. All data (replicate and repeated measurements) can be fit together as a single regression. The value of I_0 can be the quencher-free intensity for each replicate or the average of the quencher-free intensities across each replicate and repeated measurement. When fitting all replicates at once, vertical shifts between replicates will inform the fit's uncertainty without affecting the observed slope. Given that the quenching constant is purely the slope (conditioned that the intercept is 1), this approach may deflate the reported R^2 as some of the

unexplained variation does not matter for determining a Stern–Volmer quenching constant. When generalizing to the case of a single replicate with repeated measurements, this approach may bias the linear fit (unevenly spaced abscissa values); there is not a strong argument for either equally weighting all points and letting the estimator be biased or artificially half-weighting repeated measurements.

Post-averaging approaches. Each replicate may be fit independently, then the slopes averaged together (using their inverse-square uncertainties as weights) so that the regression and replicate sources of uncertainty are retained. This approach, however, does not have a straightforward method for using the quality of the y-intercept for each regression to inform the determination of the quenching coefficient.

When the number of data points is held constant (for example, by averaging repeated measurements within a replicate), fitting all data together and fitting the averaged values produce the exact estimates for the slope and intercept, but the averaged data yields smaller estimates of the uncertainty in the slope and intercept.

In comparison, for the data collected in this study, the ultimate value for the quenching constant is largely unaffected by the approach. Still, the uncertainty is more noticeably affected as might be expected (an example is shown in **Table S12**).

Table S12. Comparison of differences in quenching constants and estimated uncertainty depending on the data processing: Fitting independent replicates together (no averaging repeated measurements, each replicate using its own I_0) *vs.* Fitting I_0/I averaged across replicates and repeated measurements.

**Compound**	**Percentage Difference [a] in k_q (%)**	**Percentage Difference [a] in estimated uncertainty of k_q (%)**
Ferrocene	1.6	–18
Decamethylferrocene	0	57
1,1’-dimethylferrocene	0	67
Acetylferrocene	0	86
Benzoylferrocene	0	–29
3-nitrobenzaldehyde	–6.9	100
Methyl-4-nitrobenzoate	0	100
4-nitrobenzaldehyde	0	22
Anthracene	0	0
Acridine	0	0
Pyrene	–0.5	40

[a] (100%) × (independent – averaged) / (average)

8. References

(1) Motz, R. N.; Sun, A. C.; Lehnherr, D.; Ruccolo, S. High-Throughput Determination of Stern–Volmer Quenching Constants for Common Photocatalysts and Quenchers. *ACS Org. Inorg. Au* **2023**, *3* (5), 266–273. https://doi.org/10.1021/acsorginorgau.3c00019.

(2) Kuijpers, K. P. L.; Bottecchia, C.; Cambié, D.; Drummen, K.; König, N. J.; Noël, T. A Fully Automated Continuous-Flow Platform for Fluorescence Quenching Studies and Stern–Volmer Analysis. *Angew. Chem. Int. Ed.* **2018**, *57* (35), 11278–11282. https://doi.org/10.1002/anie.201805632.

(3) Desilets, D. J.; Kissinger, P. T.; Lytle, F. E. Improved Method for Determination of Stern-Volmer Quenching Constants. *Anal. Chem.* **1987**, *59* (8), 1244–1246. https://doi.org/10.1021/ac00135a040.

(4) Plutschack, M. B.; Pieber, B.; Gilmore, K.; Seeberger, P. H. The Hitchhiker's Guide to Flow Chemistry. *Chem. Rev.* **2017**, *117* (18), 11796–11893. https://doi.org/10.1021/acs.chemrev.7b00183.

(5) Mbanjwa, M. B.; Harding, K.; Gledhill, I. M. A. Numerical Modelling of Mixing in a Microfluidic Droplet Using a Two-Phase Moving Frame of Reference Approach. *Micromachines* **2022**, *13* (5), 708. https://doi.org/10.3390/mi13050708.

(6) Palmer, R. A.; Piper, T. S. 2,2'-Bipyridine Complexes. I. Polarized Crystal Spectra of Tris (2,2'-Bipyridine)Copper(II), -Nickel(II), -Cobalt(II), -Iron(II), and -Ruthenium(II). *Inorg. Chem.* **1966**, *5* (5), 864–878. https://doi.org/10.1021/ic50039a034.

(7) Gandolfi, M. M., Alberto Credi, Luca Prodi, M. Teresa. *Handbook of Photochemistry*, 3rd ed.; CRC Press: Boca Raton, 2006. https://doi.org/10.1201/9781420015195.

(8) Mardelli, M.; Olmsted, J. Calorimetric Determination of the 9,10-Diphenyl-Anthracene Fluorescence Quantum Yield. *J. Photochem.* **1977**, *7* (4), 277–285. https://doi.org/10.1016/0047-2670(77)85005-3.

(9) Meech, S. R.; Phillips, D. Photophysics of Some Common Fluorescence Standards. *J. Photochem.* **1983**, *23* (2), 193–217. https://doi.org/10.1016/0047-2670(83)80061-6.

(10) Suzuki, K.; Kobayashi, A.; Kaneko, S.; Takehira, K.; Yoshihara, T.; Ishida, H.; Shiina, Y.; Oishi, S.; Tobita, S. Reevaluation of Absolute Luminescence Quantum Yields of Standard Solutions Using a Spectrometer with an Integrating Sphere and a Back-Thinned CCD Detector. *Phys. Chem. Chem. Phys.* **2009**, *11* (42), 9850–9860. https://doi.org/10.1039/B912178A.

(11) *Handbook of Florescence Spectra of Aromatic Molecules*; 2012.

(12) Joung, J. F.; Han, M.; Jeong, M.; Park, S. Experimental Database of Optical Properties of Organic Compounds. *Sci. Data* **2020**, *7* (1), 295. https://doi.org/10.1038/s41597-020-00634-8.

(13) Amao, Y.; Asai, K.; Miyashita, T.; Okura, I. Photophysical and Photochemical Properties of Optical Oxygen Pressure Sensor of Platinum Porphyrin–Isobutylmethacrylate–Trifluoroethylmethacrylate Copolymer Film. *Polym. J.* **1999**, *31* (12), 1267–1269. https://doi.org/10.1295/polymj.31.1267.
